# Carbohydrate-active enzymes from a core root mycobiota member enable infection of multiple plant hosts

**DOI:** 10.1038/s41564-026-02492-3

**Published:** 2026-10-01

**Authors:** Ram-Sevak Raja-Kumar, Fantin Mesny, Arpan Kumar Basak, Jacy Newfeld, Guillaume Chesneau, Frederickson Entila, Tak Lee, Linda Rigerte, Stephanie Carvajal Acevedo, Bruno Hüttel, Pedro W. Crous, Jose G. Maciá-Vicente, Helen Stewart, Matthew Ryan, Ahmad M. Fakhoury, Soledad Sacristán, Meriem Aitouguinane, Isabelle Batisson, Stefano Dumontet, Wade H. Elmer, Jana Henzelyová, Joanna S. Kruszewska, Jessica M. Nelson, Cara M. Santelli, Markus Pauly, Antonio Molina, Kei Hiruma, Stéphane Hacquard

**Affiliations:** 1 Max Planck Institute for Plant Breeding Research, Cologne, Germany; 2 Malopolska Centre of Biotechnology, Jagiellonian University, Krakow, Poland; 3 Department of Life Sciences, Multidisciplinary Sciences, Graduate School of Arts and Sciences, The University of Tokyo, Tokyo, Japan; 4 Max Planck Genome Centre Cologne, Max Planck Institute for Plant Breeding Research, Cologne, Germany; 5 Westerdijk Fungal Biodiversity Institute, Utrecht, the Netherlands; 6 Microbiology, Department of Biology, Faculty of Science, Utrecht University, Utrecht, the Netherlands; 7 Department of Genetics, Biochemistry and Microbiology, Forestry and Agricultural Biotechnology Institute (FABI), University of Pretoria, Pretoria, South Africa; 8 Marine Sciences and Applied Biology, Multidisciplinary Institute for Environmental Studies ‘Ramón Margalef’, University of Alicante, Alicante, Spain; 9 CABI, Silwood Park, Ascot, UK; 10 School of Agricultural Sciences, Southern Illinois University, Carbondale, IL USA; 11 Centro de Biotecnología y Genómica de Plantas, Universidad Politécnica de Madrid (UPM)-Instituto Nacional de Investigación y Tecnología Agraria y Alimentaria (INIA/CSIC), Campus Montegancedo-UPM, Madrid, Spain; 12 Departamento de Biotecnología-Biología Vegetal, Escuela Técnica Superior de Ingeniería Agronómica, Alimentaria y de Biosistemas, Universidad Politécnica de Madrid, Madrid, Spain; 13 Laboratoire Microorganismes: Génome et Environnement, Université Clermont-Auvergne, CNRS, Clermont-Ferrand, France; 14 Department of Sciences and Technologies, University of Napoli ‘Parthenope’, Centro Direzionale, Napoli, Italy; 15 Department of Plant Pathology and Ecology, The Connecticut Agricultural Experiment Station, New Haven, CT USA; 16 Department of Genetics, Institute of Biology and Ecology, Faculty of Science, Pavol Jozef Šafárik University in Košice, Košice, Slovakia; 17 Institute of Biochemistry and Biophysics, Polish Academy of Sciences, Warsaw, Poland; 18 Natural History Museum, University of Oslo, Oslo, Norway; 19 Department of Earth and Environmental Sciences, University of Minnesota Twin Cities, Minneapolis, MN USA; 20 Institute of Plant Cell Biology and Biotechnology, Heinrich Heine University Düsseldorf, Düsseldorf, Germany; 21 Cluster of Excellence on Plant Sciences (CEPLAS), Max Planck Institute for Plant Breeding Research, Cologne, Germany

**Keywords:** Fungal genetics, Pathogens, Plant symbiosis

## Abstract

The root microbiome includes fungal pathogens capable of colonizing multiple plant hosts, yet the underlying genetic determinants remain unknown. Here we report that *Plectosphaerella cucumerina* is a core member of the *Arabidopsis thaliana* root microbiota, which displays pathogenic potential across multiple hosts. Using a collection of 72 *Plectosphaerella* isolates and whole-genome sequencing, we observed subtle phenotypic and genotypic variation associated with fungal phylogeny but not host plant identity. Transcriptome profiling of a *P. cucumerina* isolate in roots of diverse plants revealed core and host-specific fungal responses, including induction of carbohydrate-active enzymes (CAZymes) involved in root cell wall deconstruction. A fungal gene encoding a candidate β-1,3-glucanase (GH64) was identified as a key genetic factor driving multihost infection. This gene is present across plant-colonizing fungi and functions as a disease determinant in both *P. cucumerina* and a *Colletotrichum* root pathogen. Our results indicate that host-induced CAZymes can couple fungal virulence with multihost compatibility.

## Abstract (web summary)

Host-induced fungal CAZymes act as disease determinants and drive multihost compatibility.

## Main

Root mycobiota members can establish beneficial relationships with plants but also conditionally cause diseases or hamper plant performance^1–3^. Several core fungal taxa that reproducibly colonize roots of healthy plants are harmful under laboratory conditions^4–6^, illustrating that the pathogenic lifestyle of several root mycobiota members is often kept at bay in nature. In *Arabidopsis thaliana*, it has been reported that the protective activity of bacterial root commensals is as important as the innate immune branch involving tryptophan-derived specialized metabolites for controlling fungi in roots^2,6–8^, thereby maintaining microbial interkingdom balance for plant health^9^.

Given that soil and root-associated fungal communities are extensively shaped by the local climatic, edaphic and host conditions^10–12^, high endemism in fungal communities has been repeatedly reported^13^. Widespread fungal root colonizers appear particularly successful due to their ability to efficiently disperse, infect multiple hosts and plastically adapt to various environmental conditions^14^. However, the underlying molecular mechanisms enabling adaptation to a broad range of hosts and environments in root mycobiota members remain unknown.

Plant-associated fungi produce remarkable cocktails of carbohydrate-active enzymes (CAZymes) that facilitate fungal entry into plant tissues, promote carbohydrate extraction from host cell walls, modulate immune responses^5,15^ and contribute to antagonistic microbe–microbe interactions^16,17^. Fungal CAZyme repertoires often explain fungal lifestyles or correlate with adaptation to specific hosts or organs^18–20^. For example, the evolution of ectomycorrhizal lifestyles from saprotrophic ancestors was accompanied by extensive losses of CAZymes involved in lignin and cellulose deconstruction^21^, whereas endophytic lifestyles often retain saprotrophic capacities^1,5,8,22^. However, the extent to which the diversity, composition and transcriptional activation of CAZyme repertoires contribute to fungal adaptation to multiple hosts in root mycobiomes remains unclear.

*Plectosphaerella cucumerina* (Ascomycota, Sordariomycetes, Glomerellales,) possesses a rich CAZyme repertoire^5,19^ that may underpin its ecological flexibility as a necrotroph, saprotroph or root endophyte^23^. Primarily a soil-borne pathogen causing damping-off and root/crown rot, it can also infect leaves of the model plant *A. thaliana*^24,25^, and is increasingly recognized as an emerging pathogen of diverse crops^26^. Key gaps include how host range and host switching evolve, and how host and environmental conditions drive shifts from asymptomatic colonization to disease.

Here we show that *P. cucumerina* is a prevalent *A. thaliana* root colonizer with broad host range, causing root disease in *A. thaliana* and tomato but not in barley. Using 72 isolates, high-quality genome assemblies, multihost infection assays and fungal transcriptomics, we identify (1) host-specific activation of cell wall-degrading enzymes, (2) a key role for a GH64 β-1,3-glucanase in infection and (3) conservation of GH64 as a root infection determinant in *Colletotrichum incanum*. Our findings show that multihost adaptation relies on expanded CAZyme repertoires and their flexible regulation in response to host cues.

## Results

### *P. cucumerina* is a core member of the *A. thaliana* root mycobiota

**Fig. 1:**
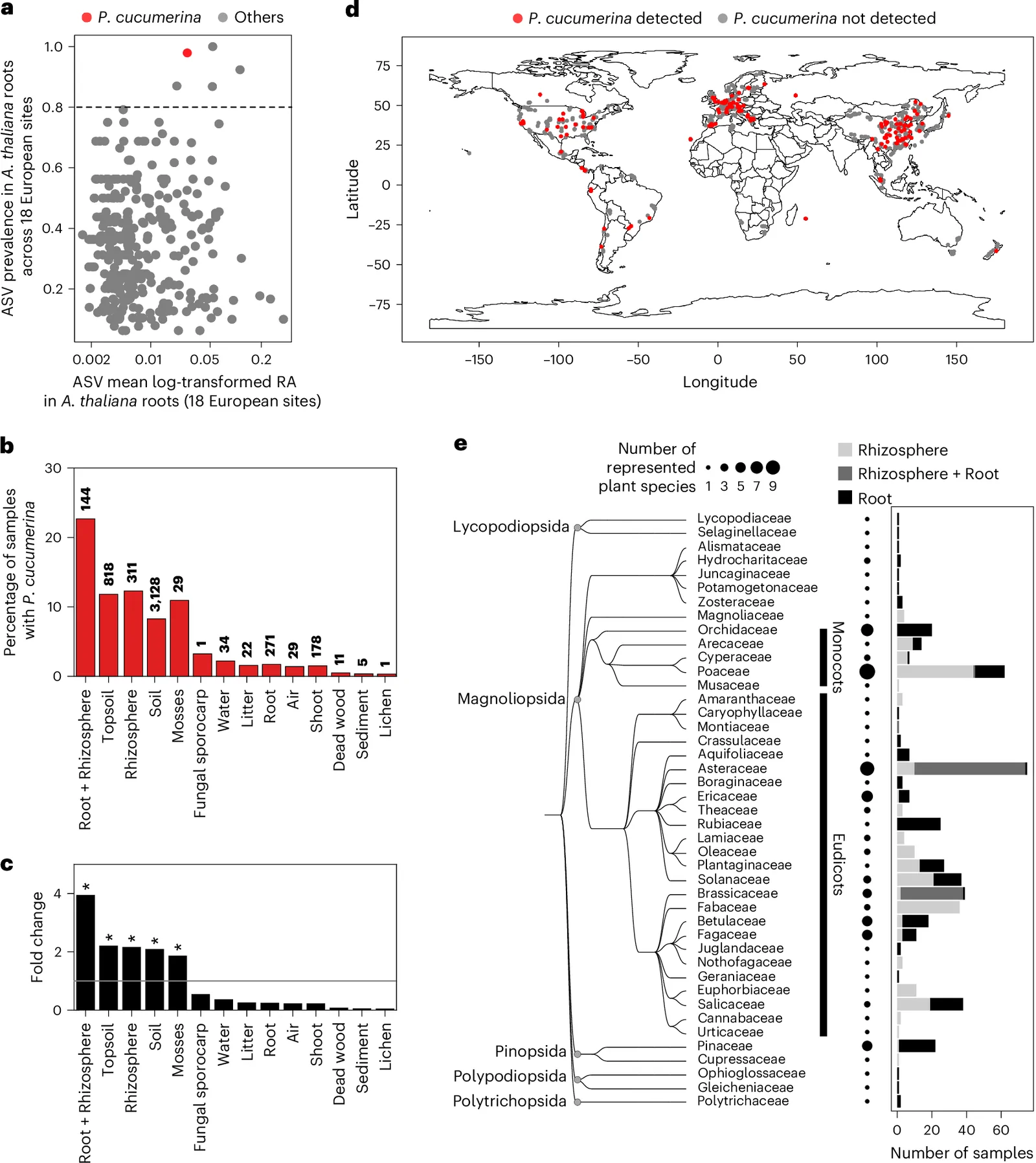
*P. cucumerina* is one of the core members of the *A. thaliana* root mycobiota and a widespread multihost root colonizer. **a**, Reanalysis of amplicon sequence variants (ASVs, that is, zero-radius OTUs (zOTUs)) sequenced from *A. thaliana* root samples (*n* = 281) from two previous studies^6,11^. zOTUs are plotted according to their prevalence and mean log-transformed relative abundance (RA) in roots across all samples. RA was calculated across samples where zOTUs were detected (minimum 0.1% per sample). Only samples having more than 1,000 reads were considered for the analysis. The 5 zOTUs above the dashed line correspond to the most widespread root-colonizing taxa. *P. cucumerina* zOTU is highlighted in red. **b**, Analysis of GlobalFungi data^31^ showing detection of *P. cucumerina* in a variety of niches (*n* = 4,982/84,844 samples). Percentage of samples in which the *P. cucumerina* species was detected are plotted for each niche, with the corresponding numbers of samples annotated on the barplot. **c**, Fold-change values corresponding to the proportion of samples from a given niche in which *P. cucumerina* was detected divided by the proportion of samples in all other niches in which *P. cucumerina* was detected. Asterisks highlight the niches in which detection of *P. cucumerina* is significantly overrepresented (pairwise one-sided Fisher’s exact tests + Benjamini–Hochberg correction; statistics, *P* values and false discovery rates (FDRs) are provided in Source Data). **d**, Geographic origin of root and rhizosphere samples of the GlobalFungi database (*n* = 19,030 samples), with samples in which the species *P. cucumerina* was detected in red (*n* = 726 samples). **e**, Cladogram presenting the phylogenetic relationships (down to the family level) of the plant hosts associated with the GlobalFungi root and rhizosphere samples in which *P. cucumerina* was detected (*n* = 726 samples). This cladogram was assembled on the basis of species names and NCBI Taxonomy data using Taxallnomy^59^. A bubble plot shows the number of species represented in each family. The stacked barplot highlights the number of samples associated with each plant host family in which *P. cucumerina* was detected, distinguishing root, rhizosphere and root + rhizosphere samples. Source data

**Extended Data Fig. 1:**
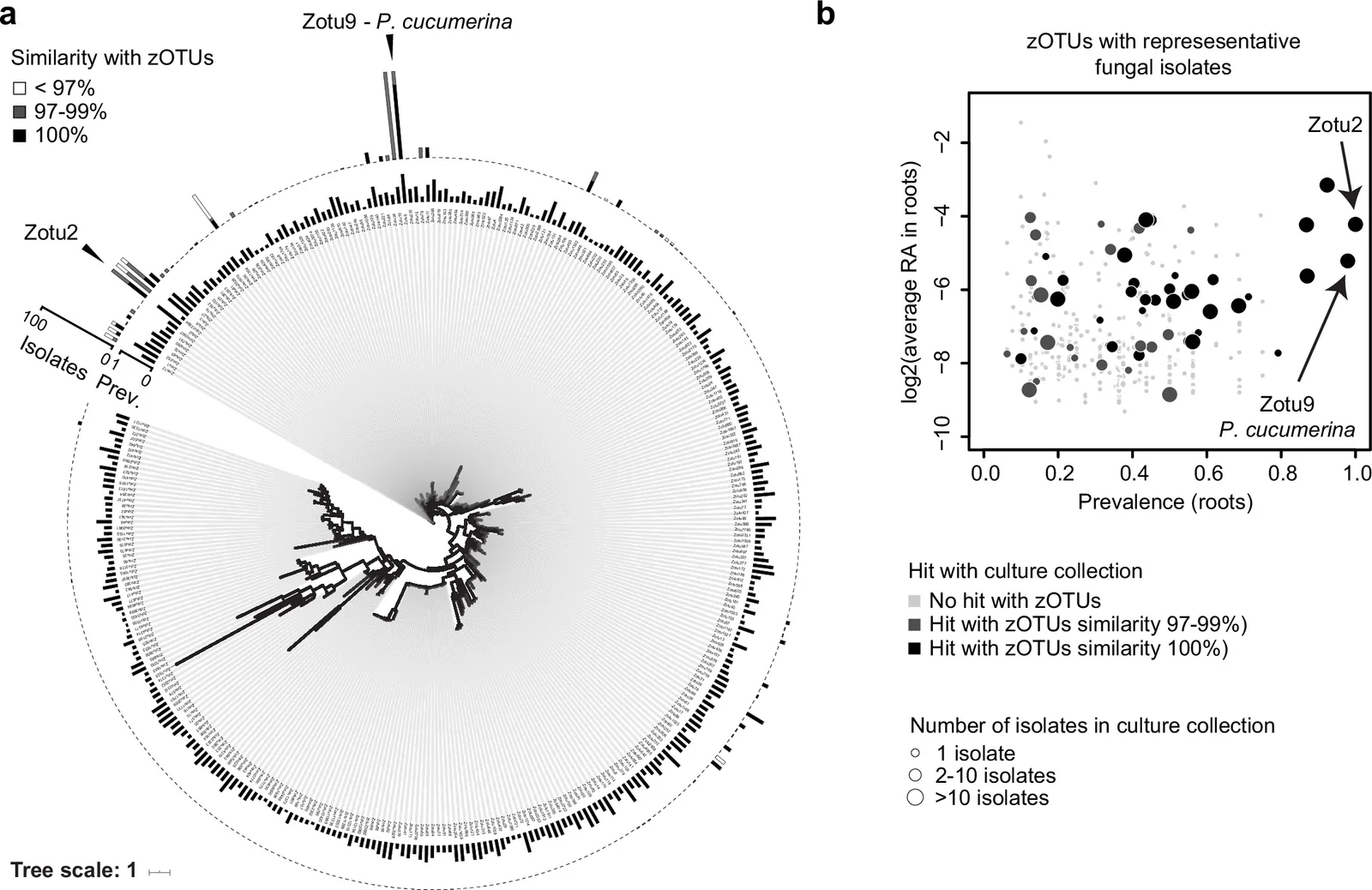
Core fungal taxa detected in Arabidopsis roots across Europe and cross-reference with fungal culture collections. **a**, Maximum likelihood tree based on ITS1 sequences of 338 zero-radius OTUs (Zotus) detected in root samples retrieved from two previous studies (n = 291 samples). This includes Arabidopsis root samples harvested from 17 European sites and from the Cologne agricultural soil. The inner circle depicts Zotus prevalence across 18 sites. The outer barplot depicts Zotus for which representative isolates match culture collection isolates (n = 488 strains, deriving from 4 sites) based on three sequence similarity thresholds ( < 97%, 97-99% and 100%). **b**, Zotus are plotted according their mean log2 RA across all root samples (n = 291 samples), relative to their prevalence across all sample sites (n = 18). RA is only calculated across samples where Zotus were present (that is, representing at least 0.1% RA per samples). Only root samples with >1,000 reads were considered for this analysis. Zotus that have hits against the ITS of cultured fungal isolates are coloured (dark grey: 97-99% sequence similarity, black: 100% sequence similarity). The size of the circle depicts the number of hits (ranging from 1 isolate to more than 10 isolates).

We hypothesized that specific root mycobiota members reproducibly colonize healthy *Arabidopsis* (Arabidopsis hereafter) roots across diverse climates and soils. We first used amplicon sequencing data from two previously published studies^6,11^ (18 sites, *n* = 291 root samples in total) and re-analysed them at amplicon sequence variant (ASV)-level resolution (that is, zero-radius operational taxonomic unit (zOTU)^27^; Methods). From the 26,976,402 sequenced reads targeting the internal transcribed spacer (ITS) fungal genomic region reads, we identified 338 zOTUs that were detected in Arabidopsis roots in at least one of the 18 sites across Europe [mean relative abundance (RA) > 0.1%; Fig. 1a and Supplementary Data 1]. Notably, we identified 5 zOTUs that were reproducibly detected in more than 80% of the 291 root samples, thereby constituting a small, yet multispecies, core Arabidopsis root mycobiota (Fig. 1a). These corresponded to zOTU2 (*Fusarium* sp.), zOTU9 (*Plectosphaerella* sp.), zOTU1 (*Dactylonectria* sp.), zOTU6 (*Fusarium* sp.) and zOTU3 (*Fusarium* sp.) that have occurrence values of 1, 0.98, 0.92, 0.87 and 0.87, respectively (Fig. 1a and Supplementary Data 1). We next focused on zOTU9 for the following three reasons: First, cross-referencing 488 ITS sequences from cultured isolates established at four of these sites (Methods and Supplementary Data 1) indicated that 18% perfectly matched zOTU9 (that is, 90 isolates, 100% sequence identity) (Extended Data Fig. 1a,b and Supplementary Data 2). Second, sequences from cultured isolates matching zOTU9 were unambiguously assigned to *P. cucumerina* on the basis of the RDP classifier^28^ (Supplementary Data 3 and Extended Data Fig. 1a,b), which represents an emergent, yet understudied, pathogen of plants, including Arabidopsis^29,30^. Last, we used *P. cucumerina* as an input species in GlobalFungi^31^ and detected this species across a variety of niches (Fig. 1b) and particularly in soil, mosses, topsoil, rhizosphere and root+rhizosphere samples (pairwise one-sided Fisher’s exact tests, Benjamini–Hochberg correction; Fig. 1c). Among the five most prevalent zOTUs, *P. cucumerina* particularly showed the strongest enrichment in ‘root+rhizosphere’ samples (Supplementary Fig. 1a), thereby confirming its presence at the soil–root interface. Root and/or rhizosphere samples in which *P. cucumerina* was detected were distributed worldwide across latitudes/longitudes and ecosystems (Fig. 1d and Supplementary Fig. 1b,c) and inspection of the corresponding plants revealed broad associations with diverse monocot and dicot hosts (Fig. 1e). The results indicate that *P. cucumerina* is a widespread root colonizer that has a broad range of hosts.

### Phylogeny, not host plant identity, explains *P. cucumerina* variation

**Fig. 2:**
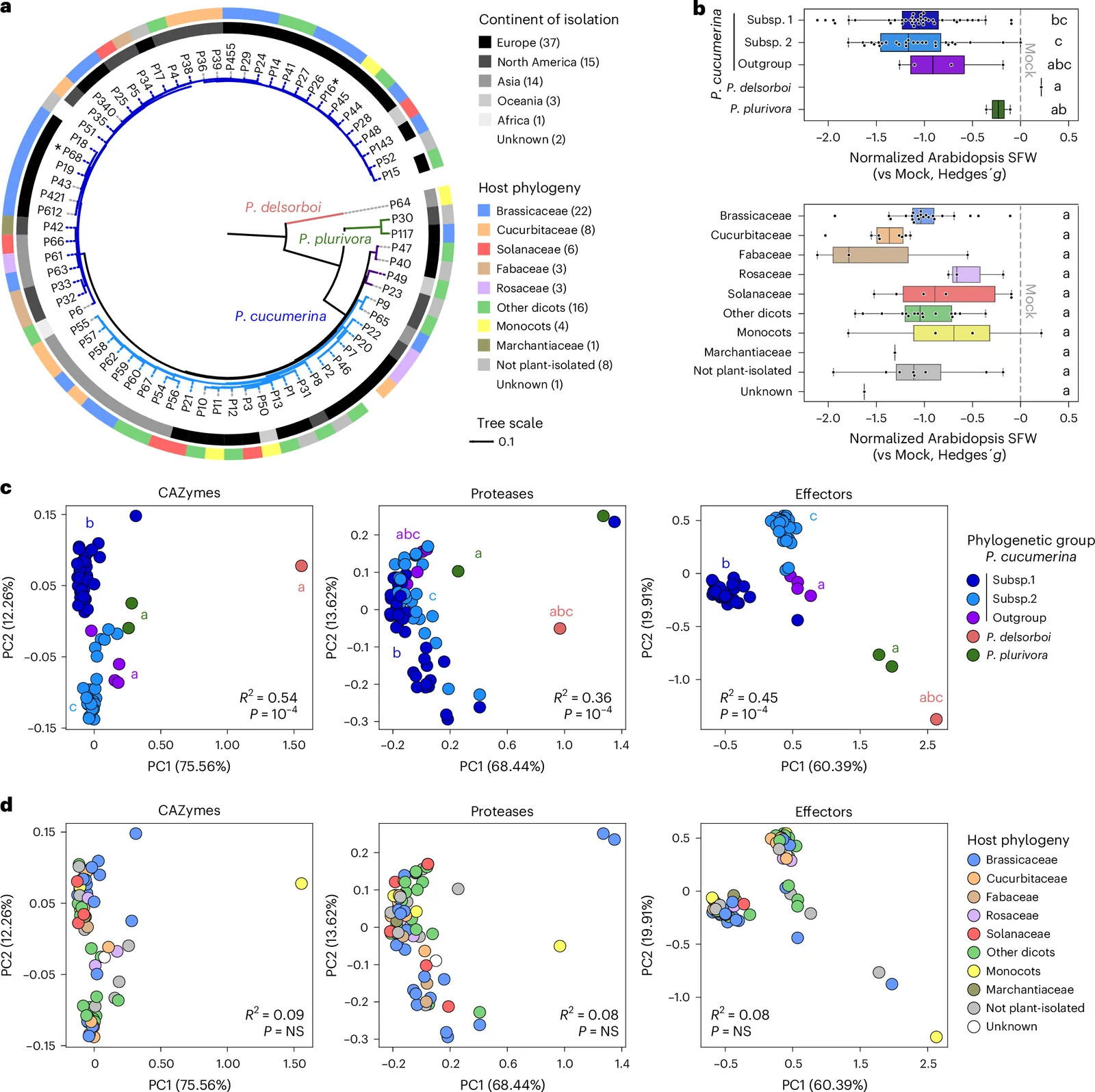
*P. cucumerina* strains isolated from phylogenetically diverse plants do not show host preference signature. **a**, Phylogeny of the 72 *Plectosphaerella* isolates, annotated with their continent of isolation (inner circle) and the phylogenetic group of their host (outer circle). The phylogenetic tree was reconstructed from the concatenated alignments of 5,466 single-copy orthologues (Methods). Two *P. cucumerina* subspecies can be distinguished and are depicted in two shades of blue whereas 4 strains do not belong to any of these subspecies and are depicted in violet (later referred to as ‘outgroup’). Isolates P68 and P16 that respectively correspond to *P. cucumerina* BMM (PcBMM, see previous work^25^) and *P. cucumerina* 0016 (see this work) are highlighted with an asterisk. **b**, Individual fungal effects (*n* = 72 strains) on Arabidopsis plants organized according to the phylogenetic group of *Plectosphaerella* strains (top), or the family names of the hosts of isolation (bottom). Standard effect sizes (Hedges’ *g*) were calculated to compare the shoot fresh weights (SFW) at 28 dpi vs mock-treated plants (agar-based gnotobiotic system, *n* = 16 plants per fungal condition). Boxplots display the median (centre line), upper and lower quartiles (bounds of box), and 1.5× interquartile range (IQR) (whiskers). Individual points outside this range correspond to outliers. Different letters indicate significant differences between categories according to two-sided Tukey HSD post hoc tests, performed after ANOVA testing (phylogenetic group: *F* = 0.49, *P* = 0.002; host family: *F* = 2.24, *P* = 0.032). **c**,**d**, PCoA (Jaccard distances calculated on family copy numbers) profiling inter-strain differences in genomic repertoires of CAZymes, proteases and candidate effectors across all *Plectosphaerella* isolates (*n* = 72 strains). The PCoA plots are colour coded according to *Plectosphaerella* phylogenetic group (**c**) or family names of the hosts of isolation (**d**). *R*^2^ and *P* resulting from PERMANOVA tests assessing the effect of these two categories on the three genomic repertoires are shown for each graph. Different letters indicate significant differences between groups based on two-sided pairwise permutation MANOVAs followed by *P*-value adjustment using the Benjamini–Hochberg method. Source data

**Extended Data Fig. 2:**
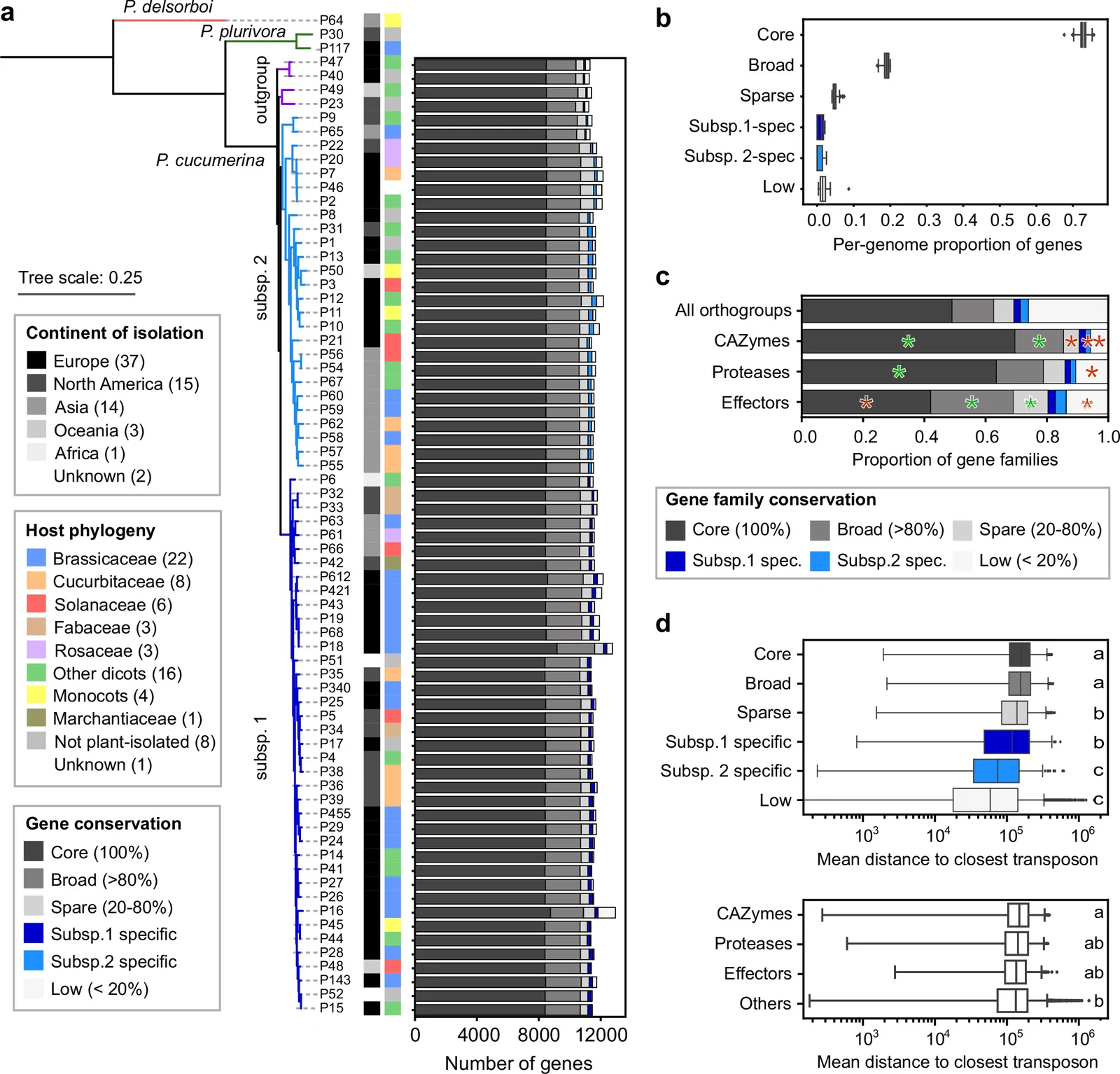
Conservation of gene families across the genomic dataset. **a**, Following orthology prediction (OrthoFinder), gene families (that is, n = 15,269 orthogroups) were analysed for their conservation across the 69 *P. cucumerina* isolates and classified as core (represented in 100% of *P. cucumerina* isolates), broadly conserved (represented in >80% of *P. cucumerina* isolates), sparsely conserved (in 20-80% of *P. cucumerina* isolates), subspecies-associated (in one subspecies’ members while absent in the other subspecies) and low-conservation (in <20% *P. cucumerina* isolates). The number of genes in each category is shown in a stacked barplot, along the *Plectosphaerella* phylogeny. **b**, Per-genome (n = 69 genomes) proportions of genes in each one of these categories are shown in a boxplot, revealing that about 75% of *P. cucumerina* gene families are ‘core’ (that is conserved across the full collection). Boxplots display the median (center line), upper and lower quartiles (bounds of box), and 1.5 × interquartile range (whiskers). Individual points outside this range correspond to outliers. **c**, Proportions of total gene families, as well as gene families encoding CAZymes, proteases and effectors, in the different intra-species conservation levels. Fisher’s exact tests comparing proportions of CAZyme, protease and effector gene families to total orthogroups were computed for each conservation level. Significant enrichments/depletions are highlighted with asterisks (green: enrichments, red: depletion). **d**, Mean distance to the closest transposon (in the same contig) were plotted for each gene family, grouped by conservation level (top; Core: n = 8195, Broad: n = 4291, Sparse: n = 1097, Subsp. 1-specific: n = 349, Subsp. 2-specific: n = 447, Low: 4291) or by gene function (bottom; CAZymes: n = 1423, Proteases: n = 454, Effectors: n = 708, Others: n = 14254). Boxplots display the median (center line), upper and lower quartiles (bounds of box), and 1.5 × interquartile range (whiskers). Individual points outside this range correspond to outliers. Letters reflect significant differences between groups according to Dunn tests, performed after Kruskal-Wallis testing (Conservation level: χ^2^ = 2409.1, *P* < 2.2 × 10^−16^; Gene function: χ^2^ = 53.49, *P* = 1.4 × 10^−11^). Source Data

**Extended Data Fig. 3:**
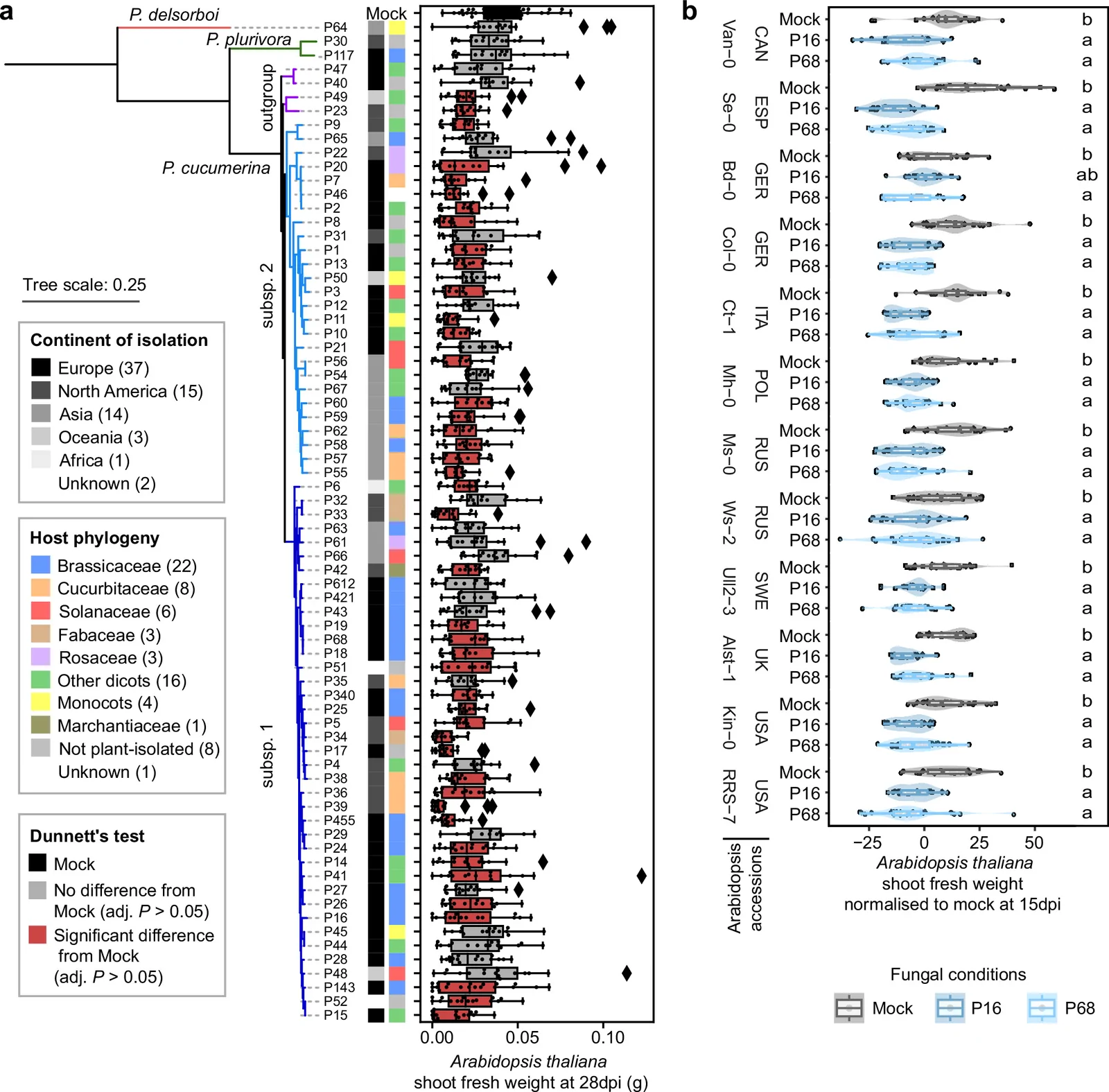
Individual fungal effects of *Plectosphaerella* isolates on *A. thaliana* performance in an agar-based gnotobiotic system. **a**, SFW of plants which roots were inoculated with 500 spores of each strain, after 28 days in culture in an agar-based gnotobiotic system (for each fungus: 4 independent biological replicates, 4 plants per replicate). These weight values reflect individual fungal effects on plants. Boxes colored in red reveal a significant difference between the fungus-inoculated and mock-treated plants, according to ANOVA (df=72, F = 4.167, *P* < 2.2 × 10^−16^) and two-sided Dunnett’s test (*P* < 0.05; individual *P* values are provided in Source Data). Boxes are represented along the strain phylogeny, reconstructed with IQ-TREE. Black diamonds represent outlier values. Boxplots display the median (center line), upper and lower quartiles (bounds of box), and 1.5 × interquartile range (whiskers). Outside this range, diamond shapes correspond to outlier data points. **b**, Box-plot/violin-plots representing the SFW of different Arabidopsis accessions 15 dpi with two Arabidopsis-derived *P. cucumerina* isolates (P16 and P68, 3 independent biological replicates and 8-10 plant replicates per condition; N ~ 62-78 per ecotype; n = 3 per treatment condition, Source Data). Conditions are represented on *y*-axes and denoted by colours (Mock-black, P16 and P68 in shades of blue) and normalised SFW (SFW_condition_ -SFW_Mock_) on x-axis. Statistical comparison within each ecotype were performed by using generalised linear model (normalised SFW ~ Treatment + Experiment) followed by Tukey’s HSD with adjusted *P* value using Benjamini-Hochberg corrections (Source Data). The letters indicate the statistical comparison between conditions. Note that isolates P68 and P16 correspond respectively to *P. cucumerina* BMM (PcBMM^30^) and *P. cucumerina* 0016 (see this work). Source Data

**Extended Data Fig. 4:**
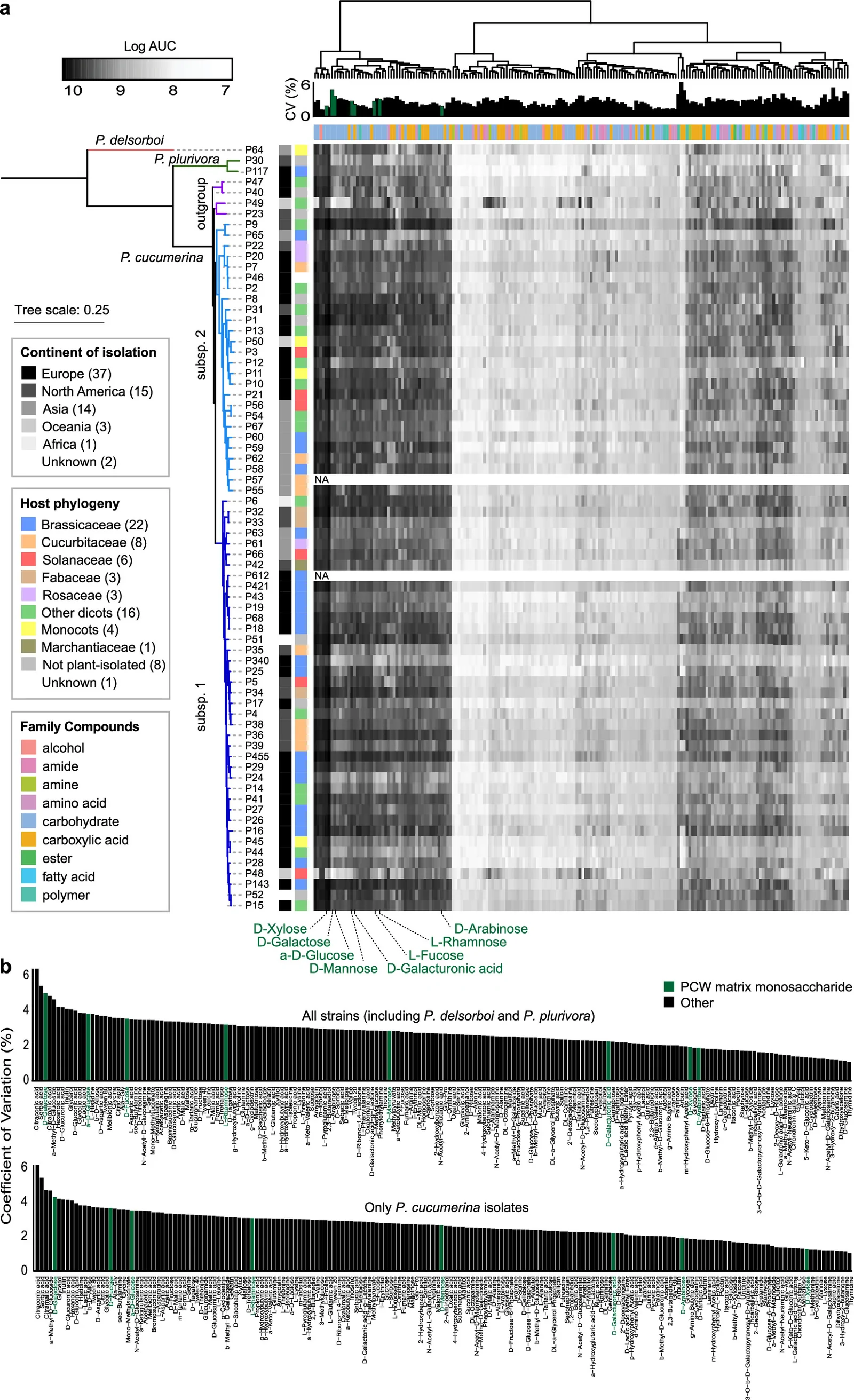
Carbon resource consumption profiles of *Plectosphaerella* isolates. **a**, Heatmap showing the respiration profiles of *Plectosphaerella* strains (n = 72, y-axis) over 5 days when grown on individual carbon sources (x-axis, n = 190) using Biolog PM1 and PM2 plates. The colour intensity represents the log-transformed area under the curve (log AUC) of respiration. Carbon sources are clustered based on their utilization patterns across all *Plectosphaerella* strains. Strains are ordered according to their phylogeny. Plant cell wall (PCW) matrix monosaccharides (Extended Data Fig. 8) are highlighted in dark green. **b**, Barplots showing the coefficient of variation (y-axis) in respiration across isolates for each carbon source (x-axis). The top panel includes all strains, while the bottom panel focuses on *P. cucumerina* isolates only. PCW matrix monosaccharides (Extended Data Fig. 8) are highlighted in dark green. Source Data

**Extended Data Fig. 5:**
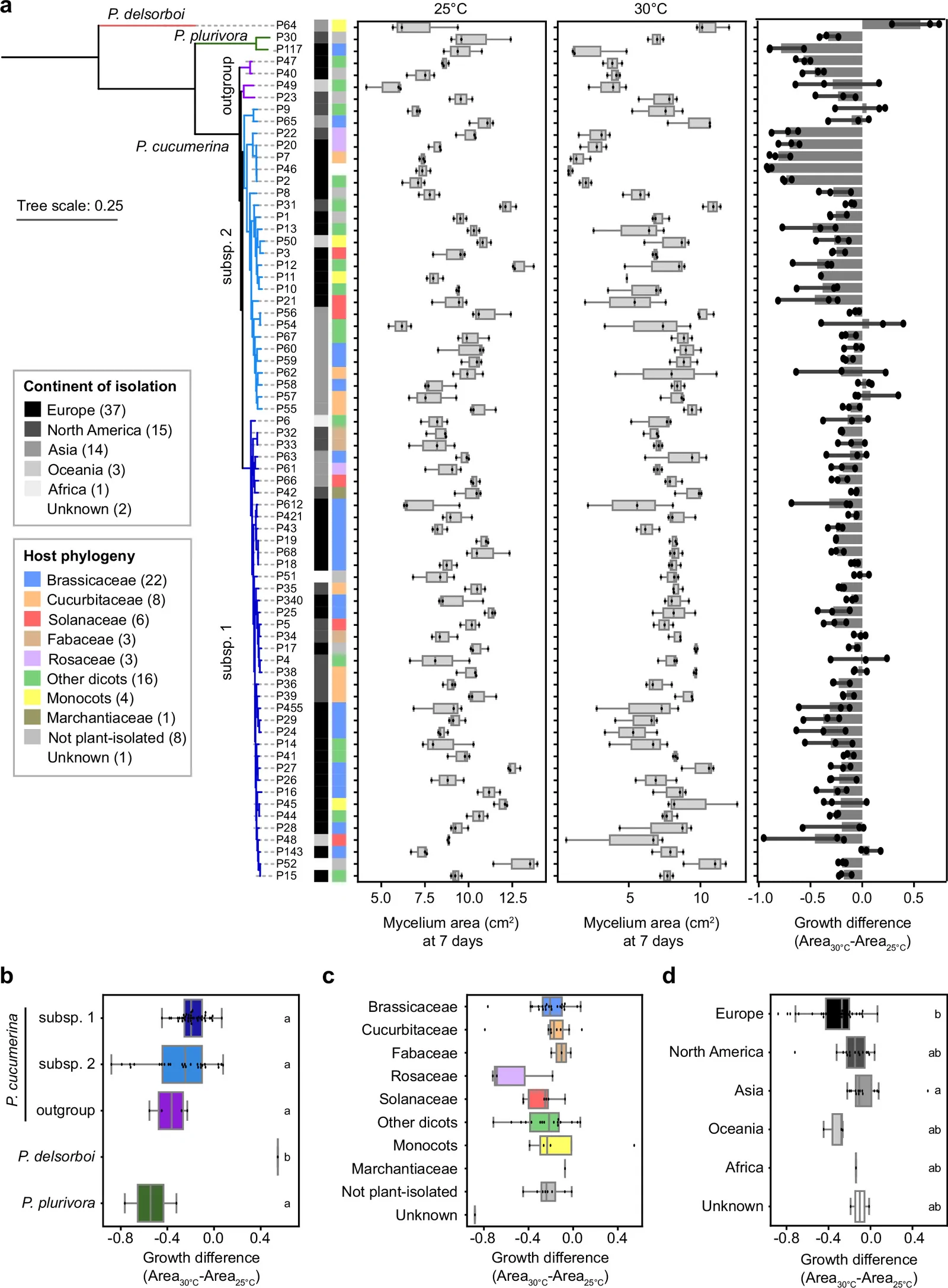
Differential growth of *Plectosphaerella* isolates at different temperatures. **a**, Along the annotated phylogeny of the *Plectosphaerella* collection, boxplots display the mycelial area of each isolate after seven days of growth on PGA medium at 25°C (left) and 30°C (center). Boxes display the median (center line), upper and lower quartiles (bounds of box), and 1.5 × interquartile range (whiskers). The accompanying barplot (right) shows the average growth difference between 30°C and 25°C for each isolate, with error bars indicating confidence intervals based on three independent biological replicates. These data reveal significant differences in temperature tolerance among the *Plectosphaerella* collection (Kruskal-Wallis test: df=71, χ^2^ = 138.91, *P* = 2.636×10^−6^). **b**, Boxplot displaying for each clade of the *Plectosphaerella* collection the per-strain mean growth difference between 30°C and 25°C (calculated over three independent biological replicates). Boxplots display the median (center line), upper and lower quartiles (bounds of box), and 1.5 × interquartile range (whiskers). Letters indicate significant differences between clades according to ANOVA test (df=4, F = 7.212, *P* = 6.84×10^−5^) and two-sided TukeyHSD test (adj.*P* < 0.05). **c**, Boxplot displaying for each host phylogenetic group in the *Plectosphaerella* collection the per-strain mean growth difference between 30°C and 25°C (calculated over three independent biological replicates). Boxplots display the median (center line), upper and lower quartiles (bounds of box), and 1.5 × interquartile range (whiskers). Individual points outside this range correspond to outliers. No significant differences among these host phylogenetic groups were identified (ANOVA test: df=9, F = 1.851, *P* = 0.0766). **d**, Boxplot displaying for each continent of isolation represented in the *Plectosphaerella* collection the per-strain mean growth difference between 30°C and 25°C (calculated over three independent biological replicates). Boxplots display the median (center line), upper and lower quartiles (bounds of box), and 1.5 × interquartile range (whiskers). Individual points outside this range correspond to outliers. Letters indicate significant differences between continents according to ANOVA test (df=5, F = 5.366, *P* = 3.37×10^−4^) and post-hoc two-sided TukeyHSD test (adj.*P* < 0.05). Source Data

We hypothesized that high genomic and phenotypic variation explains the broad fungal distribution and host range. We assembled a collection of 72 *Plectosphaerella* strains that were isolated from diverse plant families (including bryophytes^32^) and environments worldwide. Among these, 69 belong to the *P. cucumerina* species complex, and 3 belong to the closely related species *P. plurivora* (*n* = 2) and *P. delsorboi* (*n* = 1) (Supplementary Data 4 and Fig. 2a). PacBio long-read genome sequencing resulted in high-quality assemblies (sizes 35.5–40.5 Mbp, median 36.4 Mbp; N50 0.77–7.66 Mbp, median 2.69 Mbp) that contained similar sets of predicted genes (9,992–12,956 genes, median 11,581.5) (Supplementary Fig. 2a–d). Phylogeny reconstruction using 5,466 single-copy orthologues identified 2 *P. cucumerina* subspecies (subsp. 1, 39 strains; subsp. 2, 26 strains) and a *P. cucumerina* outgroup consisting of 4 strains (Fig. 2a and Supplementary Fig. 3a–d). A burst in transposon and retrotransposon elements was observed in subsp. 2 (Supplementary Fig. 4a–e) but did not associate with expansion of CAZyme, protease or effector gene repertoires (Supplementary Fig. 5). Comparative genomics across the 69 *P. cucumerina* isolates using 15,269 orthogroups identified a large core genome (>95% of genes detected in 80–100% of the strains) and a modest pan genome (<5% of the genes detected in <80% of the strains) (Extended Data Fig. 2a,b). Pan-genome-encoded genes were enriched in candidate effectors (Extended Data Fig. 2c) and more closely associated with transposons compared to core-genome-encoded genes (Extended Data Fig. 2d). Across all 72 isolates, *Plectosphaerella* phylogeny strongly structured CAZyme, protease and effector repertoires, accounting for 54%, 36% and 45% of the variance, respectively (permutational multivariate analysis of variance (PERMANOVA), *P* < 0.001, followed by pairwise permutation multivariate analysis of variance (pairwise permutation MANOVA); Fig. 2c and Supplementary Data 5). By contrast, host-of-isolation phylogeny had no detectable effect (*P* > 0.05; Fig. 2d and Supplementary Data 5a,b), indicating that host identity is not a major driver of repertoire variation in *P. cucumerina*. Similar results were observed for orthogroups and when analyses were restricted to the 69 *P. cucumerina* isolates on the basis of pairwise permutation MANOVA (Supplementary Fig. 6 and Supplementary Data 5b). We next tested whether these genetic variations associate with phenotypic differentiation for three major traits measured under artificial laboratory conditions, namely: (1) effect on *A. thaliana* development in a gnotobiotic agar-based plate system (Fig. 2b and Extended Data Fig. 3), (2) carbon resource consumption using PM1-2 Biolog Plates that contained 190 carbon sources (Extended Data Fig. 4) and (3) response to high temperature (Extended Data Fig. 5). Despite their diverse global origins, most *P. cucumerina* isolates were detrimental on Arabidopsis, irrespective of their hosts of isolation (ANOVA *P* = 2 × 10^−16^, Dunnett’s post hoc test vs mock, *P* < 0.05 for 44/69 *P. cucumerina* strains) (Fig. 2b and Extended Data Fig. 3a). Detrimental effects of two Arabidopsis-isolated strains (P16 and P68) were also retained across a panel of 12 geographically diverse Arabidopsis accessions (Extended Data Fig. 3b). All isolates also showed similar growth across 190 carbon sources (coefficient of variation <6.5%, Extended Data Fig. 4a,b) and were well adapted to efficiently use plant cell wall-derived monosaccharides as growth substrates (Extended Data Fig. 4a). They also responded negatively to elevated temperature, although effects varied by continent (Extended Data Fig. 5). Thus, the modest genetic variation among *P. cucumerina* isolates is unlikely to be the sole factor driving adaptation to the wide range of environmental and host conditions worldwide.

### Endophytic *P. cucumerina* colonization underlies disease in dicot hosts

**Fig. 3:**
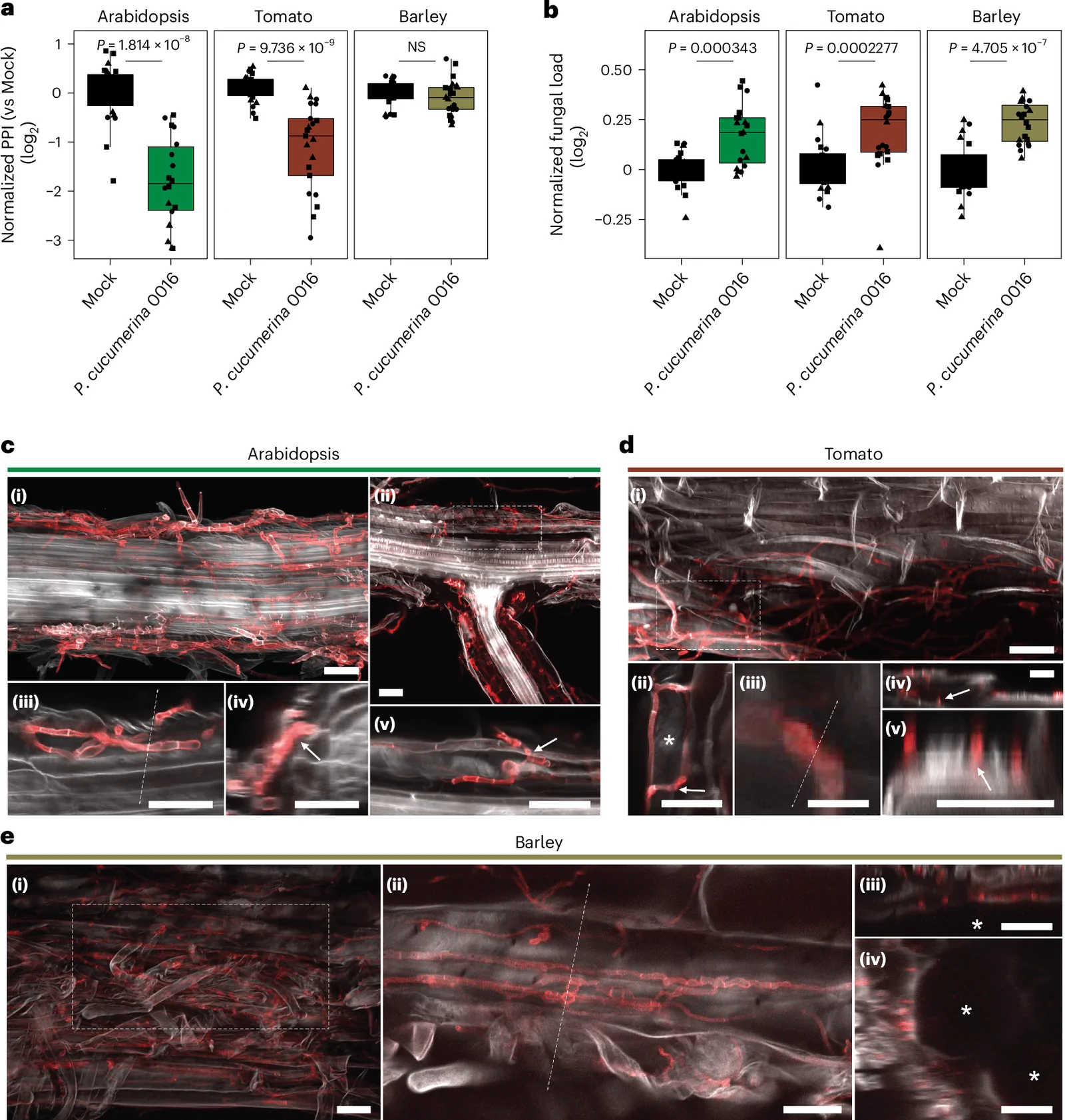
Colonization of *P. cucumerina* 0016 in Arabidopsis, tomato and barley roots and impact on host performance. **a**, PPI values of Arabidopsis, tomato and barley colonized by *P. cucumerina* 0016 were normalized to mock-treated plants and log_2_ transformed. PPI values were calculated by multiplying the survival rate by the shoot fresh weight of the plants. **b**, Relative abundance of *P. cucumerina* 0016 in the roots of Arabidopsis, tomato and barley measured by qPCR-based quantification of the fungal ITS vs plant reference gene. **a**,**b**, Pre-grown seedlings (2-week-old Arabidopsis and tomato and 1-week-old barley) were inoculated with *P. cucumerina* 0016 and harvested at 21 dpi (vermiculite-based gnotobiotic system). Three independent biological replicates are depicted (circles, triangles, squares); sample sizes (Mock/*P. Cucumerina* 0016) were: Arabidopsis *n* = 24/18, tomato *n* = 24/21, barley *n* = 24/23. Reduced sample sizes in fungal-treated groups resulted from plant mortality. Pairwise comparisons of plant performance between mock-treated and *P. cucumerina* 0016-treated plants were performed using either two-sided *t*-test or Wilcoxon rank-sum test (*P* ≤ 0.05). Boxplots display the median (centre line), lower and upper quartiles (box bounds), and the most extreme data points within 1.5× IQR (whiskers). Points beyond the whiskers are shown individually as outliers. **c**–**e**, Confocal imaging of Arabidopsis (**c**), tomato (**d**) and barley (**e**) roots colonized by *P. cucumerina* 0016 at 21 dpi. Roots were double stained with calcoflour white and wheat germ agglutinin coupled to fluorophore CF633A (WGA-CF633, Biotium) and imaged using Zeiss LSM 880 confocal microscopy. **c** (**i**), Maximum intensity projection of *Z*-stack images of an Arabidopsis primary root extensively colonized. (**ii**), Maximum intensity projection of middle section of the primary root from which a lateral root emerges. (**iii**), Enlargement of one optical section from (**i**). (**iv**), Orthogonal projection of (**iii**) showing undifferentiated hyphae penetrating peridermal cell layer (arrow). (**v**), Enlargement of one optical section shown in (**ii**) (dashed square) depicting an intracellular hypha colonizing the root cortex (arrow). **d** (**i**), Maximum intensity projection of a middle section of a primary root. (**ii**), Enlargement and *Z*-stack image of (**i**) (dashed square) showing intercellular hyphae (arrow) surrounding an epidermal cell (star). (**iii**), Enlargement of one optical section shown in (**ii**) (arrow). (**iv**,**v**), Orthogonal projection of (**ii**). Undifferentiated hyphae adhere to the epidermal cell (arrows). **e** (**i**), Maximum intensity projection of the middle section of the root. (**ii**), *Z*-stack slice of (**i**) (dashed square) showing the colonization of fungal hyphae in the epidermal layer of the barley root. (**iii**,**iv**), Orthogonal projection of (**ii**) showing hyphae adhering to epidermal cells (stars). In **c**–**e**, scale bars: 20 µm. Source data

**Extended Data Fig. 6:**
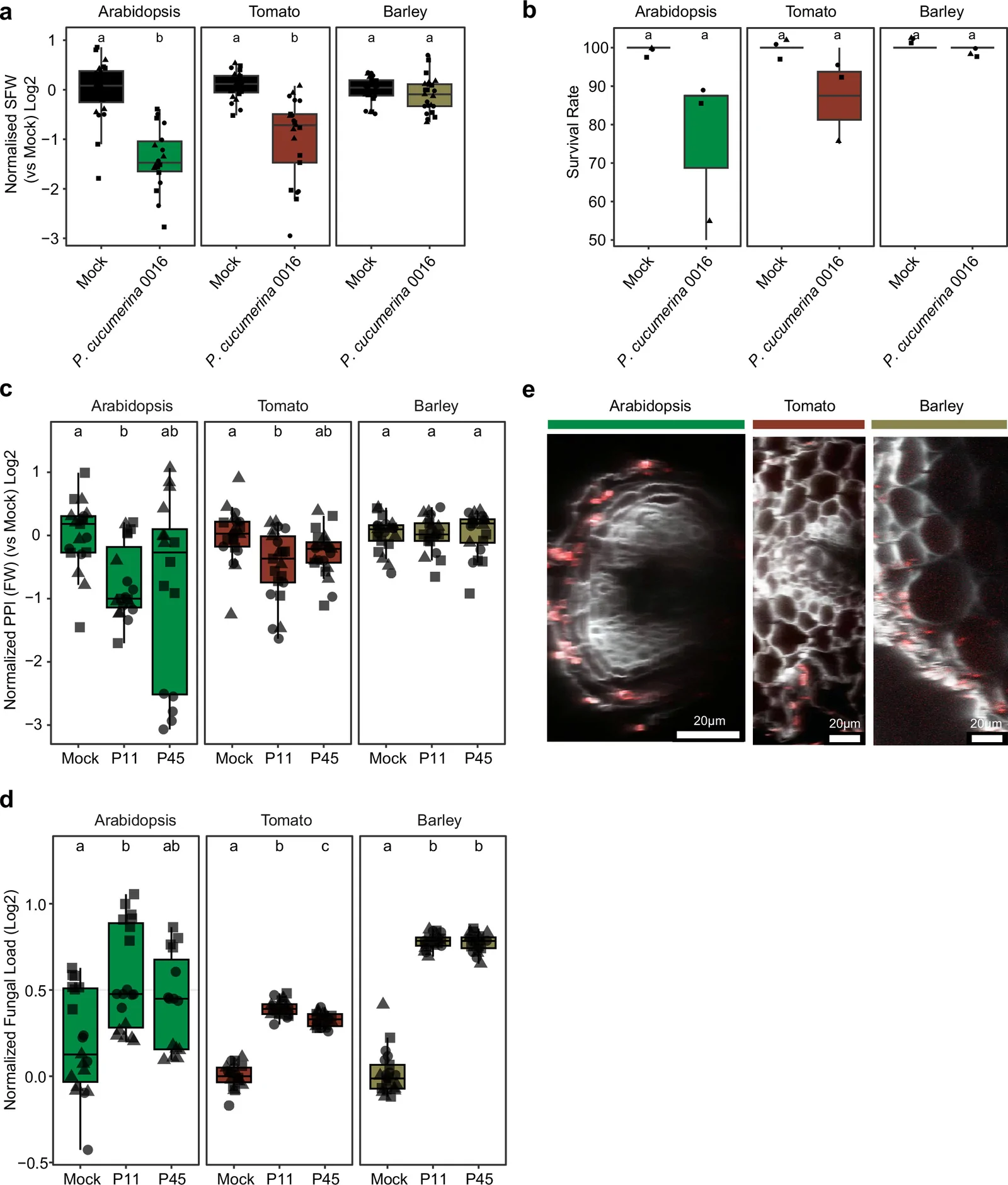
Colonization of *P. cucumerina* at the roots of Arabidopsis, Tomato and Barley and the impact of the fungus on host performance. **a**, Shoot Fresh Weight (SFW) of Arabidopsis, Tomato, and Barley colonized by *P. cucumerina* 0016 were normalized to mock-treated plants and log2 transformed. Sample sizes (Mock/*P. Cucumerina* 0016) were: Arabidopsis- n = 24/18; tomato- n = 24/21; barley- n = 24/23 with three independent biological replicates. Reduced sample sizes in fungal-treated groups resulted from plant mortality. Pairwise comparisons between mock-treated and *P. cucumerina* 0016 treated Arabidopsis, tomato, and barley plants were performed using either the two-sided t-test or Wilcoxon rank-sum test with the *P* values of 0.0001912, 3.765971e-07, 0,29 (ns)), respectively. **b**, Survival Rate of Arabidopsis, Tomato, and Barley colonized by *P. cucumerina 0016*. Pairwise comparisons between mock-treated and *P. cucumerina* 0016 treated plants were performed using either the two-sided t-test or Wilcoxon rank-sum test (*P* > 0.05). **c**, Plant Performance Indices (PPI) of Arabidopsis, tomato, and barley colonized by *P. cucumerina* (P11 and P45, both isolated from monocot hosts, see Supplementary Data 4) were normalized to mock-treated plants and log2 transformed. PPI values were calculated by multiplying the survival rate by the shoot fresh weight of the plants. **d**, RA of P11 and P45 in the roots of Arabidopsis, tomato, and barley measured by qPCR-based quantification of the fungal ITS *vs* plant *UBQ* gene. **c,d**, Multiple pairwise comparisons were performed using two-sided Kruskal-Wallis-Dunn Test, adjusted with Bonferroni correction*, P* < 0.05. Three independent biological replicates are depicted (circles, triangles, squares) and exact n values and *P* values are mentioned in the Source Data. **a-d**, Pre-grown seedlings (2-week-old Arabidopsis and Tomato and a week-old Barley) were inoculated with *P. cucumerina* and harvested 21 days post fungal inoculation. Boxplots display the median (center line), lower and upper quartiles (box bounds), and whiskers extending to the most extreme data points within 1.5 × IQR. Points beyond the whiskers are shown individually as outliers. **e**, Orthogonal projection of confocal imaging of Arabidopsis (left), Tomato (middle), Barley (right) roots colonized by *P. cucumerina* 0016 at 21 dpi. Roots were double-stained with Calcoflour white and Wheat Germ Agglutinin coupled to fluorophore CF633A (WGACF633; Biotium) and imaged using Zeiss LSM 880 confocal microscopy. Left: Fungal hyphae penetrating the cortex/periderm of Arabidopsis roots. Middle: Intercellular fungal colonization within the epidermal layer of tomato roots. Right: Fungal adherence to the epidermal cell wall of barley roots. Source Data

We next asked whether a single strain initially isolated from *A. thaliana* roots (P16, hereafter named *P. cucumerina* 0016, subspecies 1) is able to colonize/cause disease in tomato (*Solanum lycopersicum* cv. Micro-Tom) and barley (*Hordeum vulgare*). Recolonization experiments in a sterile vermiculite matrix using *P. cucumerina* 0016 and germ-free Arabidopsis, tomato and barley seedlings (Methods) revealed important effects on both shoot fresh weight (SFW) and plant survival rate 21 days post fungal inoculation (dpi) (Extended Data Fig. 6a,b). To integrate both traits, we calculated a plant performance index (PPI = survival rate × shoot fresh weight, Fig. 3a) and observed that relative to mock-inoculated controls, *P. cucumerina* 0016 significantly reduced PPI in the dicots by 19.5% in Arabidopsis and 10.7% in tomato (two-sided *t*-test or Wilcoxon rank-sum test, *P* < 0.05), but had no detectable effect on barley (*P*_adj_ > 0.05; Fig. 3a). Inspection of fungal load in roots using quantitative PCR (qPCR) indicated that the fungus was able to colonize all roots, including those of barley (two-sided *t*-test or Wilcoxon rank-sum test, *P*_adj_ < 0.05; Fig. 3b). Similar experiments conducted with two *P. cucumerina* strains isolated from monocot hosts (P11: from *Carex* sp., P45: from *Hordeum* sp.; Supplementary Data 4) confirmed these results (Extended Data Fig. 6c,d), indicating consistent effects, irrespective of whether *P. cucumerina* was originally isolated from a dicot or a monocot host. We next stained roots colonized by *P. cucumerina* 0016 using calcofluor white and WGA CF 633A for visualizing the spatial distribution of fungal hyphae during infection. Consistent with our amplicon sequencing data (Fig. 1a), numerous *P. cucumerina* 0016 extraradical hyphae were observed throughout the entire root system of Arabidopsis, from the mature peridermal region to the root tip, including lateral roots (Fig. 3c). Penetration of undifferentiated hyphae was repetitively observed into or between peridermal (Fig. 3c(i,iii,iv)) or cortical cell layers (Fig. 3c(ii,v)), thereby validating extensive endophytic propagation (Fig. 3c and Extended Data Fig. 6e). In tomato roots, epidermal walls were coated with extracellular hyphae, and endophytic colonization was primarily confined to the first epidermal cell layer (Fig. 3d and Extended Data Fig. 6e). In contrast, despite extensive fungal proliferation at the vicinity of barley root hairs, we did not observe endophytic proliferation in barley roots, suggesting that the fungus is unable to invade and/or is stopped by the barley immune system (Fig. 3e and Extended Data Fig. 6e). Taken together, our data suggest host-specific differences in fungal endophytic colonization that correlate with distinct outcomes on host performance.

### Host identity shapes *P. cucumerina* transcriptional responses in planta

**Fig. 4:**
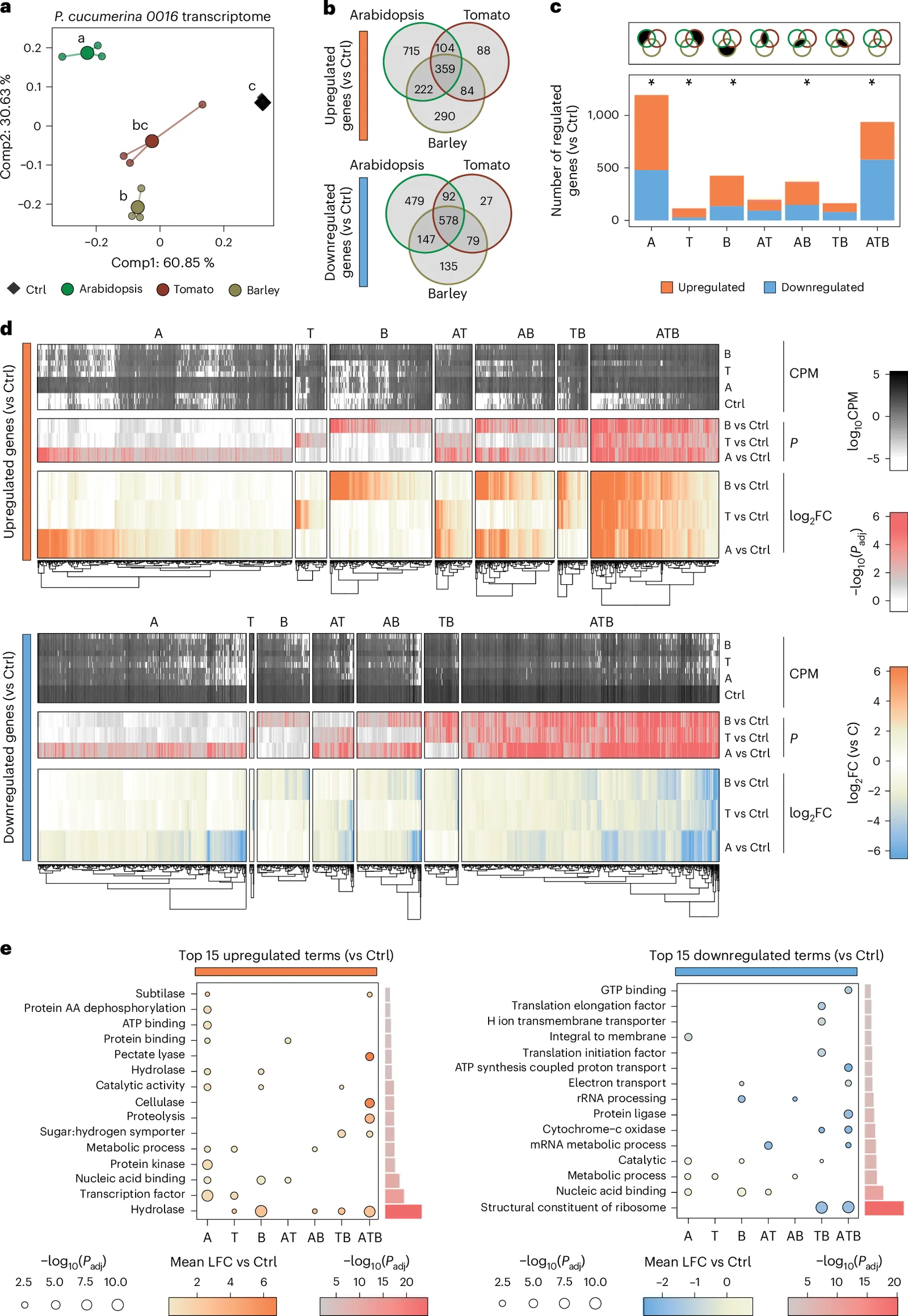
In planta transcriptome of *P.cucumerina* 0016 is modulated by host plant identity. **a**, MDS plot of Pearson distances computed on normalized read counts of differentially expressed genes (DEGs, *n* = 3,399, in planta vs Ctrl). The different plant hosts are shown in green (Arabidopsis), red (tomato) and khaki (barley). Control condition is depicted in black (Ctrl, membrane). Large data points represent the mean values of the respective datasets in MDS1 and MDS2, whereas small data points represent the 3 biological replicates. Multiple statistical comparisons across conditions were performed using Tukey HSD test (*P* ≤ 0.05). Different lowercase letters indicate statistically significant differences between groups, as represented by compact letter display (CLD). **b**, Venn diagram illustrating the number of DEGs (|log_2_FC| ≥ 1 and FDR ≤ 0.05) that are upregulated (orange) and downregulated (blue) in planta vs Ctrl condition. **c**, Stacked bar graph of fungal DEGs that are up- and downregulated in planta. The categories A, T and B denote fungal genes uniquely up- or downregulated in Arabidopsis, tomato or barley roots, respectively. AT, AB and TB denote genes commonly up- or downregulated in two hosts (Arabidopsis and tomato, Arabidopsis and barley, or tomato and barley, respectively), while ATB denotes genes consistently up- or downregulated across all hosts. Categories that are enriched in up- or downregulated DEGs in planta are marked with an asterisk (*, proportion test with Pearson’s chi-square, two-sided). **d**, Heat maps depicting DEGs that are upregulated (orange) and downregulated (blue). Bottom heat maps depict log_2_FC of DEGs that are hierarchically clustered along the *y* axis, with corresponding conditions displayed along the *x* axis. Middle heat maps depict *P*_adj_ of DEGs (Benjamini–Hochberg correction, −log_10_). Top heat maps show counts per million (CPM) of DEGs (log_10_ scale). **e**, Gene Ontology (GO) term enrichment analysis (Fisher’s exact test, alternative=‘greater’, one-sided test). Top 15 significantly enriched GO terms that are up- (left) and downregulated (right) in planta vs Ctrl. The *x* axis represents A, T, B, AT, AB, TB and ATB sectors and the *y* axis the GO terms. The colour of each sphere represents the mean log_2_FC (LFC) of DEGs, while the size of the sphere indicates the mean *P*_adj_ within each GO category for the respective conditions. The bar graph illustrates the cumulative *P*_adj_ for the respective GO. These values were computed using the hypergeometric test with Benjamini–Hochberg correction (*P* ≤ 0.05). Source data

Given the low intra-species genomic variation observed in *P. cucumerina*, we hypothesized that host-specific responses to physical and chemical cues explain its broad host range. Using the same vermiculite system described above, we sequenced the *P. cucumerina* 0016 transcriptome at 21 dpi on Arabidopsis, tomato and barley roots and compared this in planta transcriptome to that of a control fungal transcriptome obtained from inoculated polyester membranes (Ctrl, Methods). Mapping of Illumina short reads against corresponding reference genomes (Supplementary Fig. 7a,b), followed by statistical analysis of differentially expressed genes (DEGs, in planta vs Ctrl, DESeq2, generalized linear model) identified 3,399 fungal genes significantly regulated in response to the different hosts (Supplementary Data 6). Multidimensional scaling (MDS) of Pearson distances computed on normalized read counts of both expressed genes (Supplementary Fig. 8) and DEGs (Fig. 4a) identified fungal transcriptome differentiation that is primarily shaped by in planta vs Ctrl conditions (that is, *x* axis explaining 60.85% of the variation, Fig. 4a), but also to a major extent by the host plant identity (that is, *y* axis explaining 30.63% of the variation; pairwise statistical analyses: ANOVA–TukeyHSD, *P*_adj _< 0.05; Fig. 4a). Comparison of all fungal DEGs identified for each of the three comparisons (*n* = 1,862 induced, *n* = 1,537 repressed in total, in planta vs Ctrl) revealed that 19.3% and 37.6% were consistently induced and repressed, respectively, irrespective of the host plant (ATB sector, Fig. 4b,c). Consequently, most fungal DEGs responded either to two plant species (AT, AB and TB sectors, 22.0% induced, 20.7% repressed; Fig. 4b,c) or were host specific (A, T and B sectors: induced, 58.7%; repressed, 41.7%; Fig. 4b,c), thereby illustrating high transcriptome plasticity in response to different hosts (Fig. 4d). Notably, host‑specific fungal DEGs were enriched during Arabidopsis (A) infection compared with tomato (T) or barley (B) (Fig. 4c,d), mirroring successful root invasion and strong immune transcriptional responses in Arabidopsis roots (Fig. 3c and Supplementary Fig. 9). Fungal Gene Ontology (GO)-term enrichment analysis (*P*_adj_ < 0.05; Benjamini–Hochberg method) for each of the above-mentioned DEG sectors (Fig. 4c) revealed enrichment of terms related to catabolism that were exclusively observed in the upregulated gene sets (Fig. 4e and Supplementary Fig. 10a,b). These included: ‘Hydrolase’, ‘Proteolysis’, ‘Cellulase’, ‘Catalytic activity’, ‘Pectate lyase’ or ‘Subtilase’, that were often identified as enriched in both host-specific (A, T, B) and shared (ATB) fungal DEGs subsets. Taken together, our results revealed that *P. cucumerina* transcriptional reprogramming is differently tailored depending on the colonized host and that catabolism of host-derived products is probably key in driving infection across a broad range of hosts.

### In planta CAZyme induction reflects root cell wall composition

**Fig. 5:**
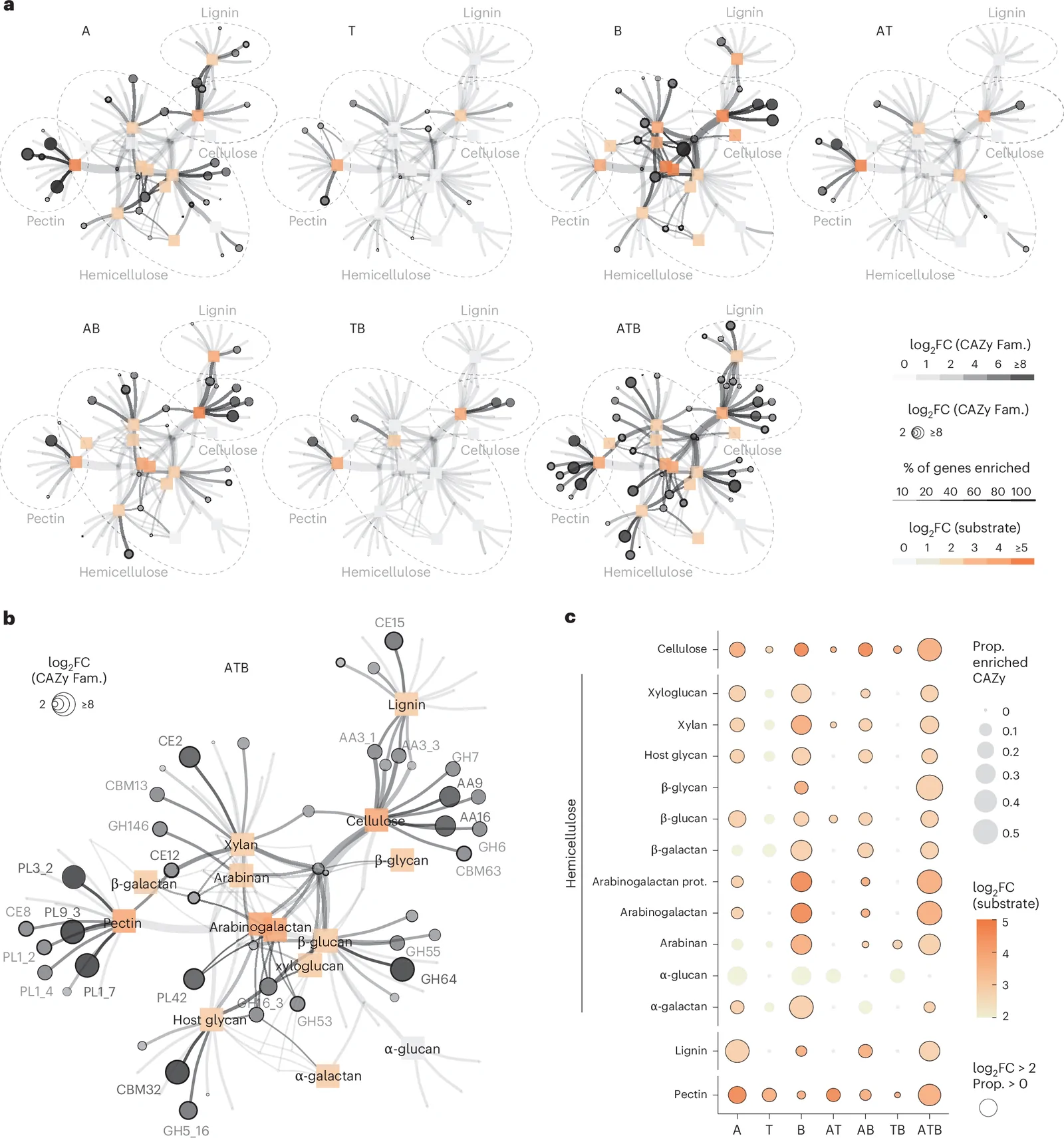
Host-specific and core sets of *P. cucumerina* 0016 CAZyme-encoding genes induced during root colonization of different hosts. **a**,**b**, Networks illustrating CAZyme families acting on plant and plant/fungal cell walls (circles) alongside their potential plant cell wall substrates (*n* = 14 substrates, squares) defined on the basis of the CAZyDB^87^ and dbCAN-sub databases^33^. The colour gradient of the nodes and edges (white to black) and the size of the circles reflect the mean log_2_FC of statistically enriched CAZyme family in planta vs Ctrl (Fisher’s exact test, alternative=‘greater’, one-sided test with Benjamini–Hochberg correction.) for each DEG subset (A, T, B, AT, AB, TB, ATB (**a**). **b**, Enlarged ATB network (see Fig. 4c). For each CAZyme family, the thickness of the circles indicates the proportion of genes induced among the DEGs across conditions. Predicted substrates targeted by CAZymes are shown using a grey-to-orange gradient that depicts the mean log_2_FC of the enriched CAZymes targeting specific substrates across conditions. Substrates represented in grey squares are classified as non-significant (with a proportion of enriched CAZymes equal to 0). **c**, Balloon plot illustrating the utilization of substrates through the differential expression of fungal CAZymes based on their log_2_FC in planta vs Ctrl. The colours within the circles (yellow-to-orange) depicts the mean log_2_FC of the CAZymes predicted to target primary cell wall substrates such as cellulose, diverse hemicellulose components, lignin or pectin (see *y* axis) for the different DEGs subsets (see *x* axis, A, T, B, AT, AB, TB and ATB). The size of the circles corresponds to the proportion of DEGs within the CAZyme family that target the different cell wall substrates (*y* axis). Circles outlined in black represent groups that show a mean log_2_FC along with a proportion of enriched CAZymes >0, and those outlined in grey represent groups where the proportion of enriched CAZymes equal to 0, as determined by Fisher’s exact test with *P*_adj_ < 0.05. Source data

**Extended Data Fig. 7:**
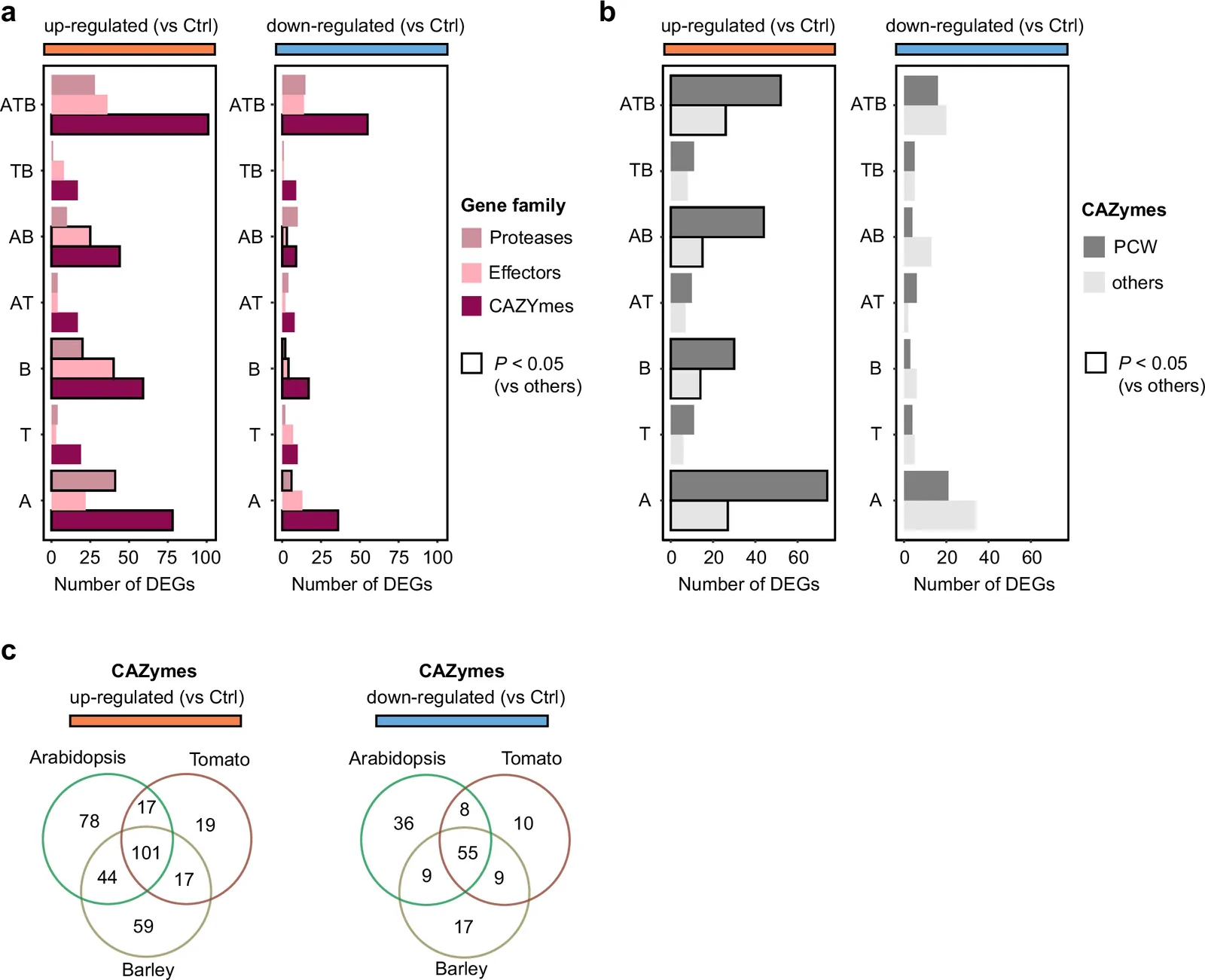
Regulation of CAZyme-encoding genes during root colonization. **a,b**, The combination of barplot illustrates the number of differentially expressed genes (DEGs, x-axes) that are either upregulated (left, denoted in orange) or downregulated (right, denoted in blue) for the shared sectors on the y-axes. Marked bars signify those sectors that are *P* ≤ 0.05 (prop.test, chi-square statistics, with alternative = ‘greater’, one-sided) indicating the ratio of gene family to be elevated among the total DEGs in the dataset. The colours of the barplot elucidate the predetermined gene families Proteases (deeppink), Effectors (lightpink) and CAZYmes (magenta) (a) and in grey plant-cell-wall components (PCW; mined ‘pectin, cellulose, lignin, arabinan, galactan, glycan, glucan, xylan, galacturon) CAZY-Substrate (dbCAN^48^) (b). **c**, Venn diagram illustrating the number of DEGs encoding CAZymes ( | log2FC | ≥1 and FDR ≤ 0.05) that are up-regulated (orange) and down-regulated (blue) *in planta*, respectively. Source Data

**Extended Data Fig. 8:**
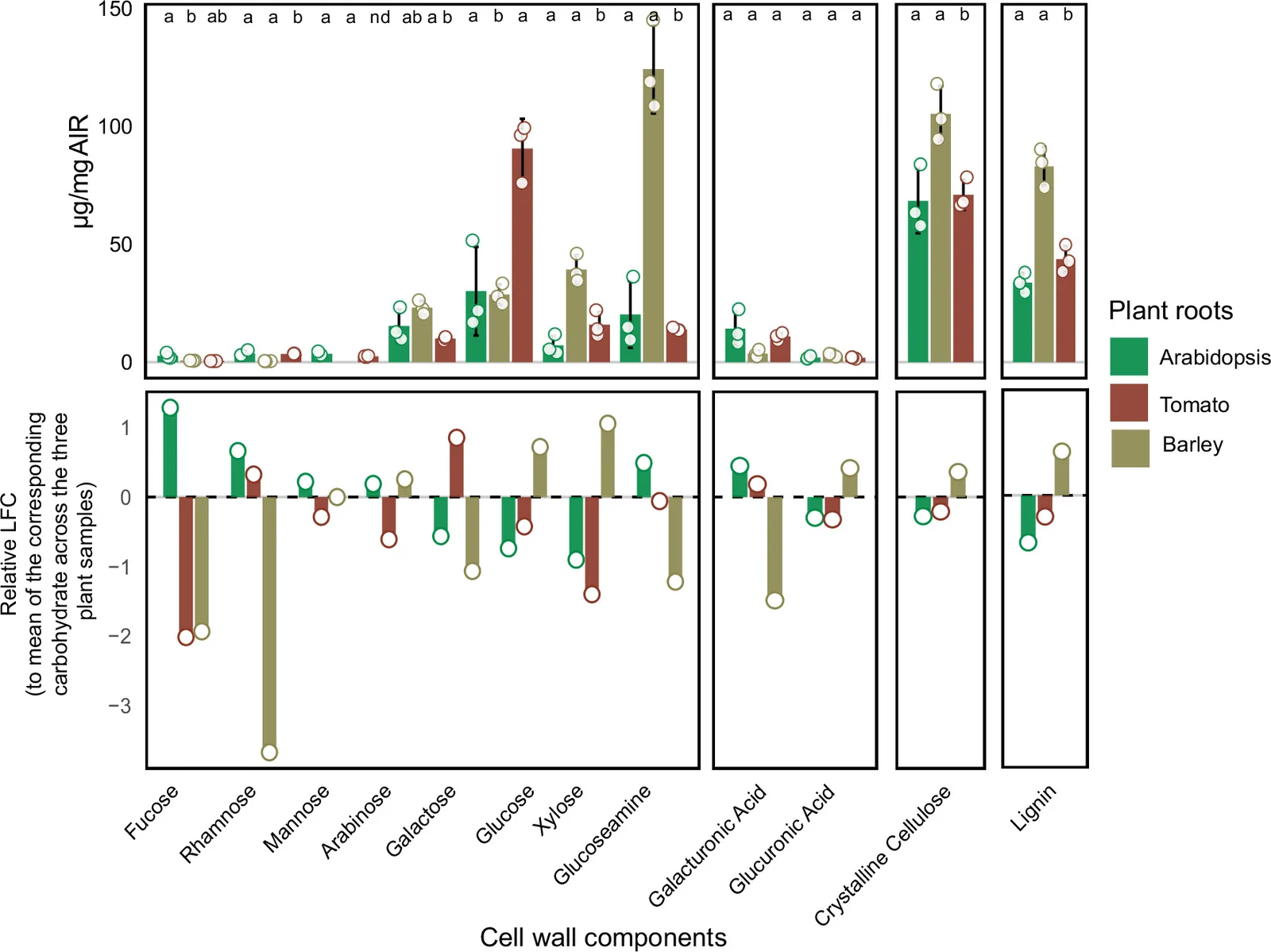
Root cell wall composition analysis of Arabidopsis, tomato, and barley. The upper barplot illustrates the concentration of wall matrix monosaccharides, including uronic acids [in µg/mg alcohol insoluble residue AIR, y axis], cellulose, and lignin detected in roots of 5-week-old *A. thaliana* (n = 36 plants) and tomato (n = 18 plants), and 4-week-old barley (n = 6 plants) with three independent biological replicates (n = 3). The cell wall components were arranged based on the different assays used and in descending order based on their abundance in Arabidopsis. Multiple pairwise comparisons between the three plant species for each cell wall component were performed using two-sided Kruskal-Wallis-Dunn Test, adjusted with Bonferroni correction (*P* ≤ 0.05). The lower barplot illustrates the relative enrichment of carbohydrates (log2FC) for each plant with respect to the mean value measured across all three plants. Data derived from three biological replicates. The exact *P*, mean, and standard deviation are provided in the Source Data. Source Data

We next hypothesized that *P. cucumerina* 0016-mediated cell wall (CW) degradation prevails during root colonization and plastically adjusts according to host CW polysaccharide composition. Consistent with our GO-term enrichment analysis (Fig. 4e), we observed that genes encoding CAZymes were disproportionally upregulated during root infection in all above-mentioned DEG sectors (A: 78/715 = 10.9%, T: 19/88 = 21.6%, B: 59/290 = 20.3%, AT: 17/104 = 16.3%, AB: 44/222 = 19.8%, TB: 17/84 = 20.2%, ATB: 101/359 = 28.1% of the DEGs; Extended Data Fig. 7a–c), thereby contrasting with the expected proportion of CAZyme genes encoded in *P. cucumerina* 0016 genome (9.9%). Next, we constructed a force-directed network map (Methods), where circular nodes represented plant CW-degrading CAZyme families (*n* = 83, Methods) connected to their predicted plant substrates (as registered in the dbCAN-sub database^33,34^) (*n* = 14, Methods). By mapping the mean log_2_FC of CAZyme families significantly overexpressed in planta (see circle size and grey gradient, log_2_FC, Fisher’s exact test, *P* < 0.05) onto the network backbone for each above-mentioned DEG sector, we predicted host CW components (square nodes, orange gradient, log_2_FC, Fisher’s exact test, *P* < 0.05) that were targeted by the fungus in response to a specific host (A, T, B; Fig. 5a), two hosts (AT, AB, TB; Fig. 5a) or all three hosts (ATB Fig. 5a,b). This revealed that diverse CAZymes were predicted to target all major CW constituents (pectic polysaccharides, cellulose, hemicellulose, lignin), irrespective of the host plant (ATB, Fig. 5a–c). These CAZymes belong to polysaccharide lyase (PL) families 1_7, 3_2, 9_3 and 42, the carbohydrate binding module family 32, the glycoside hydrolase (GH) family 64, lytic polysaccharide monooxygenases families AA9, AA16 and the carbohydrate esterase family 2 (Fig. 5b). CWs of dicotyledonous plants are more pectin rich than those of monocots, while monocot walls contain mainly structurally diverse xylans and mixed-linkage glucans as their dominant hemicelluloses^35^.This is consistent with our targeted analysis of wall materials in roots of Arabidopsis, tomato and barley, as pectic uronic acids were more prevalent in Arabidopsis and tomato, while (arabino)-xylan and matrix polysaccharide-based glucose were more prevalent in barley (Extended Data Fig. 8). Interestingly, these differences correlated with host-specific activation of fungal genes encoding pectin-degrading enzymes in the dicot hosts (A, T, AT; PL3_2, PL 1_7, PL11_2 families), or (arabino)xylan-degrading enzymes in the barley host (B; GH51_2) (Fig. 5a,c). Our results suggest that sensing and adequate transcriptional response to CW physicochemical cues contribute to plastic infection of diverse hosts in *P. cucumerina*.

### Fungal *GH64* promotes multihost infection and disease

**Fig. 6:**
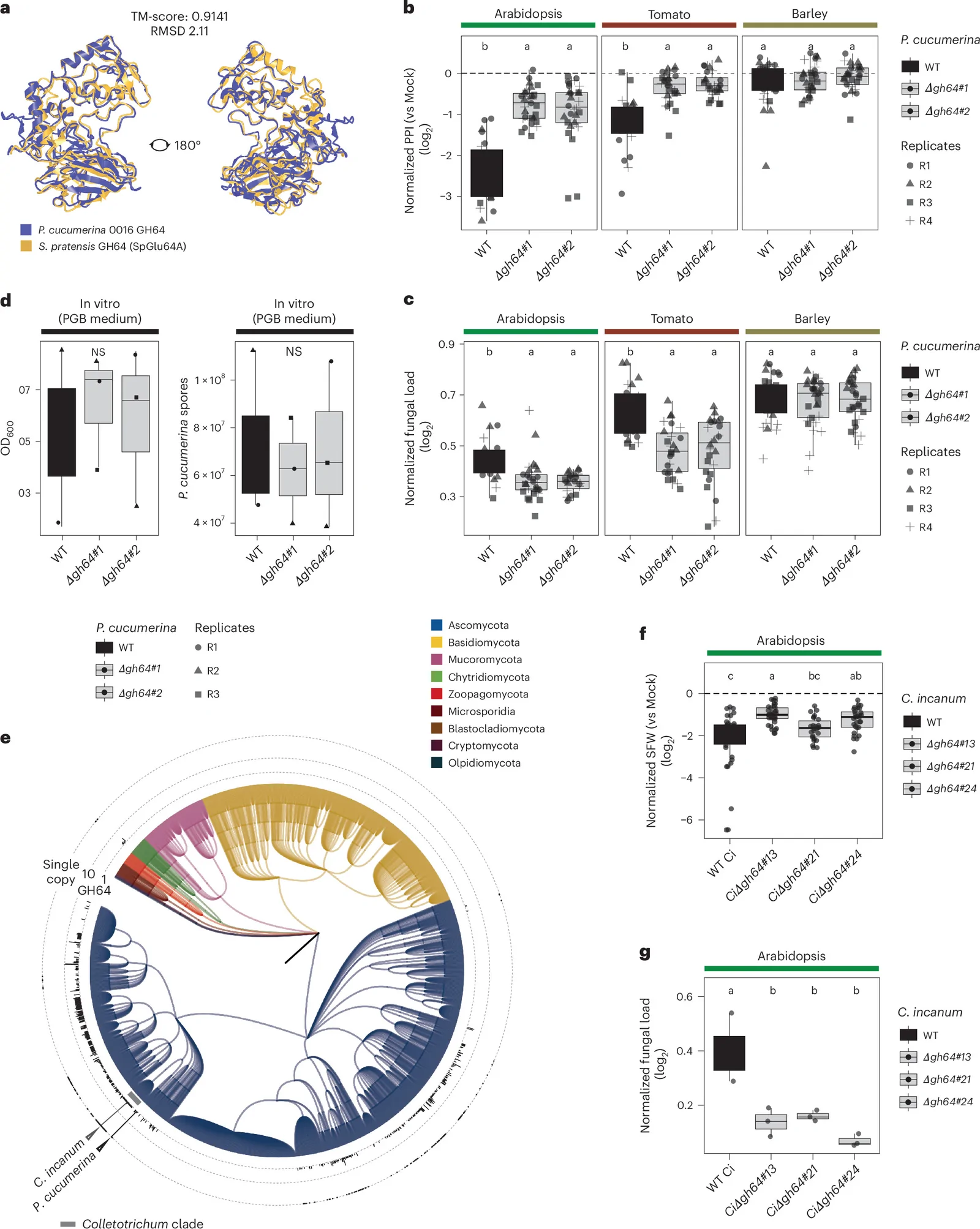
Phenotype assessment of *P. cucumerina* 0016 *and C. incanum Δgh64* mutant strains. **a**, AlphaFold3**-**predicted structure of *P. cucumerina* 0016 GH64 (in blue; predicted template modelling (pTM) = 0.92) showing high similarity to the previously resolved structure of the GH64 enzyme SpGlu64A of *Streptomyces pratensis*^37^ (in orange), according to FoldSeek search analysis^36^. RMSD, root-mean-square deviation. **b**, PPIs of Arabidopsis, tomato and barley colonized by *P. cucumerina* 0016 were normalized to mock-treated plants and log_2_ transformed. PPI values were calculated by multiplying the germination rate by the shoot fresh weight of the plants. **c**, RA of *P. cucumerina* 0016 in the roots of Arabidopsis, tomato and barley measured by qPCR-based detection of the fungal ITS vs plant *UBQ10* gene. In **b**,**c**, pre-grown seedlings (2-week-old Arabidopsis and tomato and 1-week-old barley) were inoculated with *P. cucumerina* 0016 and harvested at 21 days post fungal inoculation. Four independent biological replicates are depicted (circles, triangles, squares and plus), sample sizes (WT/*Δgh64#1*/*Δgh64#2*) were: Arabidopsis *n* = 22/32/30, tomato *n* = 23/29/28, barley *n* = 31/32/30. Multiple pairwise comparisons of plant performance between mock-treated and *P. cucumerina* 0016-treated plants were performed using two-sided Kruskal–Wallis–Dunn test, adjusted with Bonferroni correction (*P* ≤ 0.05). **d**, In vitro growth assessment of *P. cucumerina* 0016 WT and *Δgh64* mutant strains. An initial spore inoculum of 8 × 10^4^ spores per ml was used and the fungus was inoculated on PGB medium for 24 h. OD_600_ was measured after 24 h using a spectrophotometer (left) and yield of *P. cucumerina* 0016 spores were counted after 24 h using a haemocytometer (right). **e**, Phylogenetic tree constructed using 2,534 fungal genomes from the MycoCosm^62^ (JGI) with NCBI taxonomy indices. The colours of the clade indicate the fungal phylum. The barplot at the leaf nodes denotes the number of GH64 occurrences that are identified in the genomes (CAZyme-family GH64 from JGI mycocosm). The outer barplots highlight fungi that encode a single-copy *GH64*. The *Colletotrichum* clade is highlighted in grey. **f**, SFW of *A. thaliana* plants colonized by *Colletotrichum incanum* wild type (WT *Ci*) and 3 independent mutant lines (*CiΔgh64#13*, *#21*, *#24*) normalized to mock-treated plants (dashed line) and log_2_ transformed. Plants (3 independent biological replicates) were harvested at 24 dpi. Sample sizes (WT/*CiΔgh64#13*/*ΔCigh64#21*/*ΔCigh64#24*) were *n* = 38/35/26/30, respectively. Statistical significance was determined using two-way ANOVA–TukeyHSD, *P* < 0.05. **g**, Relative load of WT *Ci* and mutant strains in the roots of *A. thaliana* measured by reverse transcription–quantitative polymerase chain reaction (RT–qPCR)-based detection of *C. incanum* ACTIN transcripts normalized to Arabidopsis ACTIN transcripts at 24 dpi (*n* = 3 biological replicates per condition; individual sample values are provided in Source Data). Statistical significance was determined using two-way ANOVA–TukeyHSD, *P* < 0.05. All boxplots display the median (centre line), lower and upper quartiles (box bounds), and the most extreme data points within 1.5× IQR (whiskers). Points beyond the whiskers are shown individually as outliers. In **b**,**c**,**f**,**g**, all exact *P* values for the multiple pairwise comparisons are provided in the Source Data file and different lowercase letters indicate statistically significant differences between groups, as represented by CLD. Source data

**Extended Data Fig. 9:**
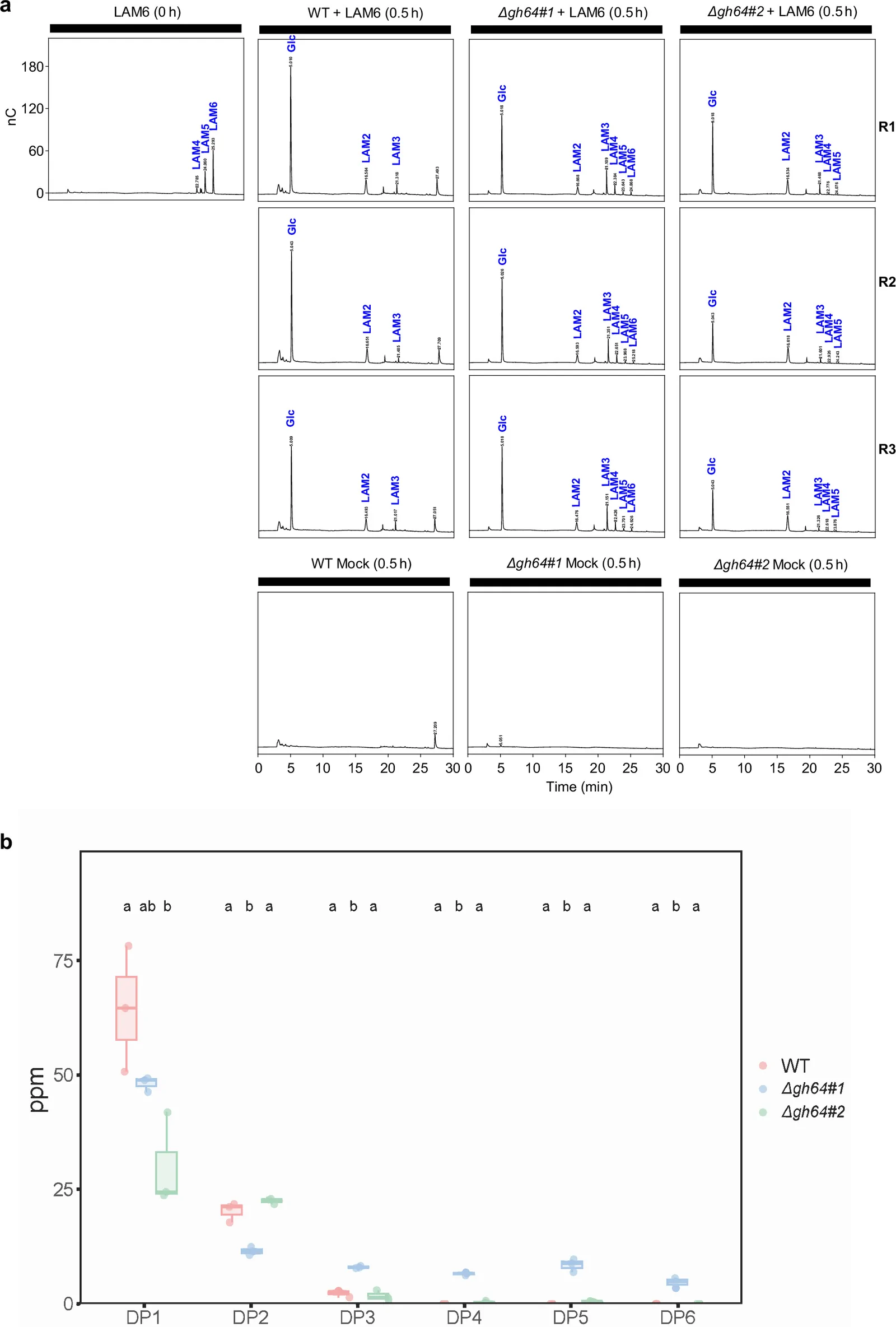
*In vitro* degradation of Laminarihexaose (LAM6) by *P. cucumerina* 0016 and *Δgh64* mutants. **a**, HPAEC-PAD chromatographic profiles of oligosaccharides released after 30 min incubation of WT, *Δgh64#1*, and *Δgh64#2* spores with laminarihexaose (LAM6) as the sole carbon source. Three biological replicates are shown for each strain. Controls include LAM6 alone (0 h prior to incubation) and mock samples (H_2_0). Indicated peaks correspond to glucose and laminari-oligosaccharides with different degree of polymerization (DP2-DP6). **b**, Quantification of released oligosaccharides expressed as ppm across degrees of polymerization (DP1-DP6). Box plots represent the distribution of three biological replicates (n = 3). The internal line within each box indicates the median (second quartile, Q2). The lower and upper limits correspond to the first (Q1, 25th percentile) and third (Q3, 75th percentile) quartiles, respectively. Whiskers extend to the most extreme observations within 1.5 × the interquartile range (IQR) of the lower and upper quartiles. Statistical significance was analysed separately for each DP (based on the distribution and homogeneity of the data) using either one-way ANOVA followed by Fisher’s LSD post-hoc tests with Benjamini–Hochberg adjustment (DP1 and DP2) or Kruskal–Wallis test with post-hoc multiple comparison with Benjamini–Hochberg adjustment (DP3–DP6). The exact global *P* values were: DP1, *P* = 0.00874; DP2, *P* = 5.8 × 10⁻^5^; DP3, *P* = 0.0237; DP4, *P* = 0.0145; DP5, *P* = 0.0145; and DP6, *P* = 0.0237. Different letters show statistically significant differences between groups after multiple-comparison correction. Source Data

**Extended Data Fig. 10:**
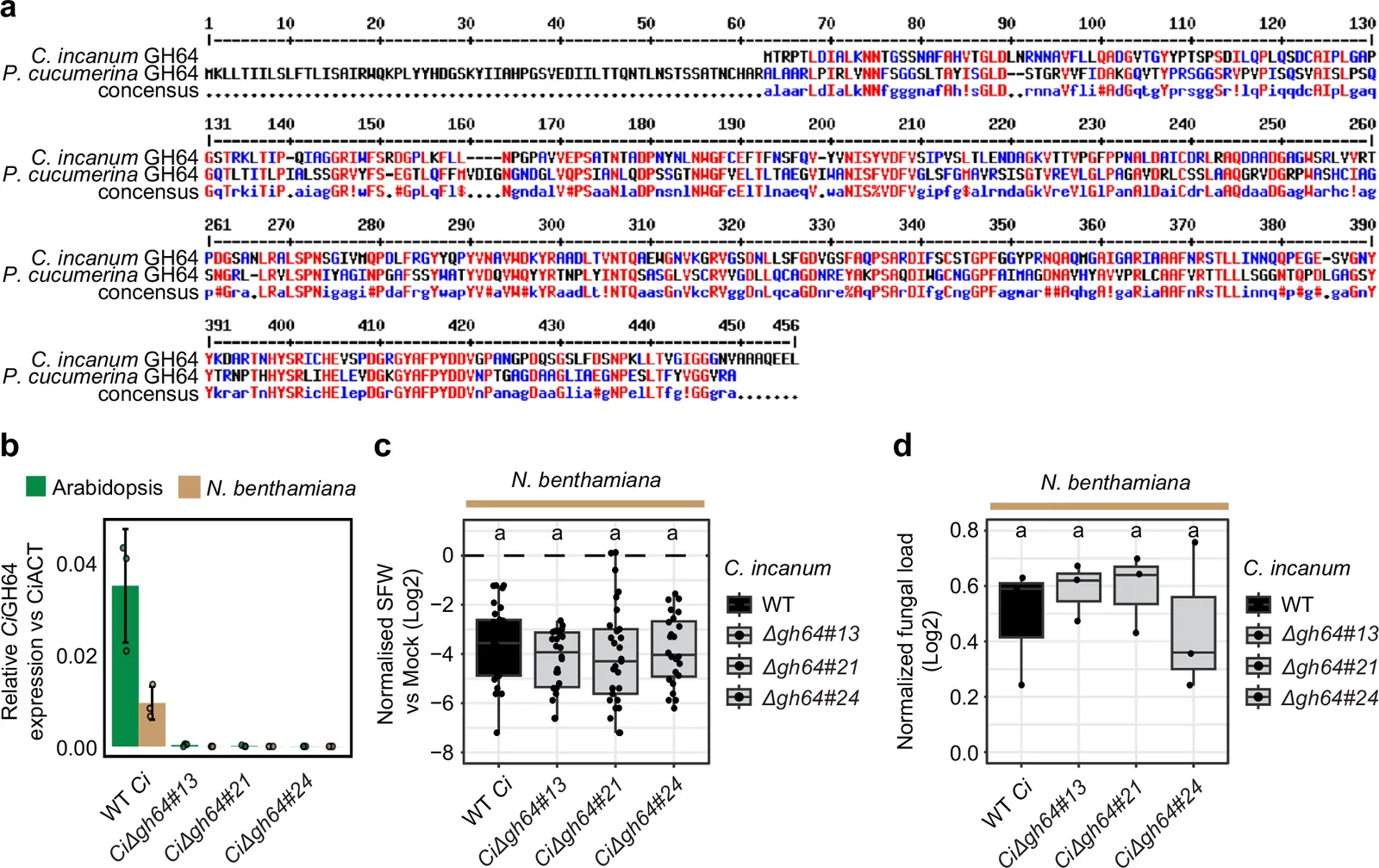
*C. incanum* GH64 sequence conservation, expression and functional relevance for *N. benthamiana* root infection. **a**, Protein sequence alignment of *P. cucumerina* 0016 GH64 (bottom) with *C. incanum* GH64 (up) obtained from Multalin^135^. Note the absence of signal peptide in *C. incanum* GH64 sequence. **b**, *GH64* transcript quantification in roots of *N. benthamiana* and Arabidopsis. The relative expression of the *C. incanum GH64* gene was measured by qRT-PCR on *N. benthamiana* or Arabidopsis roots inoculated with *C. incanum* WT (WT *Ci*) and *Δgh64* mutant lines and normalized to the expression of *C. incanum ACTIN* gene at 24 dpi. The exact n, mean, and standard deviation are provided in the Source Data. **c**, Normalised SFW of *N. benthamiana* plants inoculated with WT *Ci* or *Δgh64* mutants and normalized to mock-treated plants (24 dpi). Statistical significance was evaluated by two-sided Tukey’s post-hoc test indicated by different letters (*P* < 0.05). **d**, Relative fungal load quantification of WT and mutant lines in *N. benthamiana* roots was estimated by qRT-PCR based quantification of *C. incanum ACTIN* transcripts normalized to *N. benthamiana ACTIN* transcripts (24 dpi). Statistical significance was evaluated by two-sided Tukey’s post-hoc test indicated by different letters, *P* < 0.05. **c,d**, Boxplots display the median (center line), lower and upper quartiles (box bounds), and whiskers extending to the most extreme data points within 1.5 × IQR. Points beyond the whiskers are shown individually as outliers. **c,d**, All the experiments are performed in three biological replicates and the exact *P* and n values are provided in the Source Data. Source Data

On the basis of our network map (ATB, Fig. 5b), we identified a single-copy gene encoding a secreted GH64 enzyme (jgi|Plecuc1|282412) that displayed one of the highest log_2_FC enrichment in planta, irrespective of the host plants (Arabidopsis log_2_FC = 7.4, tomato log_2_FC = 8.3, barley log_2_FC = 8.3; Supplementary Data 6). Following AlphaFold protein structure prediction, a FoldSeek^36^ search identified strong structural similarity with the SpGlu64A enzyme secreted by *Streptomyces pratensis A* (Fig. 6a and Supplementary Fig. 11a), a β-glucanase exhibiting strict substrate specificity towards β-1,3-glucans^37^. These β-1,3-linked glucose units are constituents of plant CWs (that is, callose and grass-specific hemicellulosic mixed-linkage glucans), but mainly represent immunogenic molecules of fungal CWs^38–41^. Transfection of *P. cucumerina* 0016 protoplasts with a Cas9–single guide (sg)RNA ribonucleoprotein complex enabled precise homologous recombination-mediated insertion of a phleomycin-resistance DNA cassette into the GH64 coding sequence (Methods and Supplementary Fig. 12a–d). Whole-genome resequencing confirmed a single insertion at the intended locus and found no evidence of off-target edits (Supplementary Data 7). Using Lam6 (a β‑1,3‑glucan hexasaccharide) as the sole carbon source followed by high-performance anion-exchange chromatography with pulsed amperometric detection (HPAEC–PAD), we observed a rapid hydrolysis to glucose by the wild-type (WT) strain (within 30 min) and slower hydrolysis by both mutant strains, consistent with GH64’s contribution to β‑1,3‑glucanase activity (Extended Data Fig. 9). Next, mutant spores were inoculated on germ-free roots of Arabidopsis and tomato seedlings. Inspection of PPI at 21 dpi revealed 62–77% reduction in pathogenicity (Kruskal–Wallis–Dunn test, adjusted with Bonferroni correction*, P* < 0.0001; Fig. 6b) and 18–24% reduction in root colonization in tomato and Arabidopsis plants (Kruskal–Wallis–Dunn test, adjusted with Bonferroni correction*, P* < 0.0001; Fig. 6c) compared to the WT strain. In contrast, no significant differences were observed in the barley host (*P* > 0.05, Fig. 6b,c), or in artificial growth media (Fig. 6d and Supplementary Fig. 12e,f), indicating that the gene is a root colonization determinant of the two tested dicot hosts. WT-like disease phenotypes were also observed for another mutant (*Δpl11_2*, Supplementary Fig. 13), excluding the possibility that phleomycin resistance is influencing these phenotypes. Notably, GH64 shows high sequence conservation across the 72 *Plectosphaerella* isolates (Supplementary Fig. 11b), has orthologues in specific fungal lineages belonging to Sordariomycetes and Dothideomycetes, and is significantly enriched in genomes of plant saprotrophs, pathogens and endophytes (*n* = 2,534 fungal genomes, Fig. 6e and Supplementary Fig. 11c,d). Notably, multiple strains in the destructive fungal genus *Colletotrichum* carry a single-copy *GH64* gene (Fig. 6e and Supplementary Data 8). To probe its broader function, we used the genetically tractable pathogen *Colletotrichum incanum*, which infects Arabidopsis roots and induces GH64 expression in planta according to previous RNA-seq data^22^. Inactivation of the *C. incanum GH64* gene via homologous recombination^2,22,42^ (class Sordariomycetes, family Glomerellaceae, jgi|Colin1|11312|; Extended Data Fig. 10a) resulted in 68.5% reduction of fungal mutant load in *A. thaliana* roots and 10% increase in shoot fresh weight compared to the WT *C. incanum* strain (24 dpi, Fig. 6f,g). However, no significant differences were noted in the Solanaceae host *Nicotiana benthamiana* which is explained by a ~75% reduction in *C. incanum GH64* expression in *N. benthamiana*- versus Arabidopsis-colonized roots (Extended Data Fig. 10b–d). Taken together, our data indicate that in planta activation of this gene in evolutionarily distant fungal lineages promotes root infection and disease.

## Discussion

Our study identifies *P. cucumerina* as a prevalent root colonizer across diverse hosts and one of the few core members of the Arabidopsis root mycobiota. Given that core fungal isolates can be pathogenic in mono-association^5^ but are often restrained by bacteria^6,7,9^, *P. cucumerina*-induced disease in nature probably requires the multihost infection determinants identified here, together with mechanisms of root microbiome manipulation^43^.

The ability of *P. cucumerina* to colonize multiple hosts is explained by a remarkably large repertoire of CAZymes acting on diverse plant CW substrates. In comparison with 93 other plant-associated Sordariomycete fungi^5^, *P. cucumerina* 0016 encodes an unusually diverse repertoire of plant cell wall-degrading enzymes (PCWDEs, 12th of 94, *n* = 160, range 198–29) and displayed the highest percentage of PCWDEs within fungal CAZyme repertoires (1st of 94, 76%, range 76%–33%)^5^. Our within-species comparative genome analysis among 69 *P. cucumerina* isolates revealed little variation in gene repertoire categories, including CAZymes (range 1,212–1,397, median 1,291), which did not correlate with variation in fungal detrimental activity. Therefore, among-strain phenotypic variation in pathogenicity probably arose from differences in gene expression, rather than in gene repertoire composition^19,22,44,45^.

We also show that *P. cucumerina* differentially expresses CAZymes in response to host-derived cues, potentially enabling multihost compatibility. Root-infecting fungi colonizing both dicot and monocot hosts can respond to markedly different root CWs, similar to broad-host-range leaf pathogens^46,47^. *P. cucumerina* dispersion success in nature probably depends on its capability to infect multiple hosts at any sites to avoid a population bottleneck. The primarily asexual lifestyle of the fungus^23^, together with the low genetic and functional diversity observed across strains, is consistent with *P. cucumerina* being a generalist pathogen that achieved broad distribution via a conserved, diverse and highly tuneable repertoire of CAZyme-encoding genes.

We showed that endophytic *P. cucumerina* colonization promotes pathogenicity in the two dicot hosts and requires the candidate β-1,3-glucanase GH64. We propose that this gene drives fungal proliferation in the root endosphere and connects multihost colonization to disease. This is consistent with previous evidence linking aggressive endophytic colonization to disease^4,5^. Deletion of the orthologous gene in the root pathogen *C. incanum* also prevented root infection and disease in *A. thaliana*. However, the gene was dispensable for *N. benthamiana* root infection, consistent with its low expression in the roots of this host. Our data reveal that GH64 is a root infection determinant in at least two fungal pathogens that evolved independently in distinct families within Sordariomycetes.

β-1,3-glucans represent a diverse group of polysaccharides that are found in both plant and fungal CWs^48^. They are targeted by CW-degrading enzymes of root-colonizing fungi and subject to host immune surveillance^40^^,49^^,50^. Therefore, pathogenic fungi evolved mechanisms to overcome β-1,3-glucan-mediated immune activation. In *Magnaporthe oryzae* and a fungal insect pathogen, β-1,3-glucanases Ebg1 (ref. ^15^) and BbEng1 (ref. ^51^), respectively, hydrolyse or deplete immunogenic β-1,3-glucans to evade host immune surveillance. Therefore, suppression of β-1,3-glucan-induced immune activation by GH64 could represent one strategy deployed by *P. cucumerina* to promote endophytic infection in roots of Arabiodopsis and tomato. Furthermore, *P. cucumerina* could also deploy this GH64 enzyme in planta to break down callose, a plant CW polysaccharide composed of β-1,3-linked glucose units^52^ that accumulate at pathogen infection sites and in plasmodesmata to block pathogen invasion^53,54^. Irrespective of the mode of action, experiments with the β‑1,3‑glucan hexasaccharide LAM6 further corroborated that *P. cucumerina* is very efficient at hydrolysing it into glucose and that GH64 contributes to this activity. *GH64* and potentially other host-inducible fungal genes encoding β-1,3-glucanases therefore represent targets of choice to efficiently control destructive root pathogens that have a broad host range.

## Methods

### Reanalysis of *A. thaliana* mycobiota profiling data

Data from a European transect study profiling the root mycobiota of *A. thaliana* at 17 sites were reanalysed^11^, together with profiling data of *A. thaliana* roots grown in Cologne agricultural soil^6^. Paired sequencing reads were joined and demultiplexed using QIIME^55^ and its scripts ‘join paired reads’ and ‘split libraries fastq’. A quality-filtering step was performed with phred score of 30 or higher. For fungal reads that could not be merged, the respective forward reads were kept for further processing. Filtered and demultiplexed reads were trimmed to an equal length of 220 bp using USEARCH with the option ‘-fastx truncate’^56^. These reads were dereplicated and forwarded to the UNOISE3 pipeline^27^. zOTUs (clustering threshold at 100%, often referred to as ASVs) were determined using the USEARCH unoise3 command, where reads with sequencing errors were corrected and possible chimaeras removed, leaving a set of correct amplicon sequences. zOTU reads were checked against an ITS sequences database (full-length ITS sequences from the National Center for Biotechnology Information (NCBI)) to remove non-fungal reads. Fungal zOTUs were classified using the Warcup database^57^. To receive count tables, zOTU sequences were then mapped back against the whole read dataset at a 97% similarity cut-off (using USEARCH -usearch global).

### *P. cucumerina* niches, hosts and global soil prevalence

The GlobalFungi online database^31^ was accessed (April 2026) to identify ecological niches inhabited by the five core mycobiome zOTUs. zOTUs were associated with a single ‘species hypothesis’ (SH) by performing a GlobalFungi sequence search with their ITS1 sequence (zOTU1: SH0736260.10FU; zOTU2 and zOTU6: SH0730983.10FU; zOTU3: SH0730975.10FU; zOTU9: SH1002406.10FU). Samples including each SH were compared to total samples registered in the database. Overrepresentation of a niche in samples in which *P. cucumerina* (SH1002406.10FU) was detected was tested with a one-sided Fisher’s exact test, using the formula ‘stats.fisher_exact(alternative=’greater’)’ in Scipy^58^, followed by Benjamini–Hochberg *P*-value correction. To identify plant species colonized by *P. cucumerina*, ‘root’, ‘rhizosphere’ and ‘root+rhizosphere’ samples containing *P. cucumerina* were browsed and the ‘dominant plant species’ sample metadata were collected. These plant species were grouped by phylogenetic class, according to the NCBI Taxonomy database. The plant phylogeny (Fig. 1) was obtained using Taxallnomy^59^ based on NCBI Taxonomy data.

To analyse the prevalence of *P. cucumerina* in soils, previously curated ITS1-5.8S-ITS2 rDNA sequences of the species^23^ were downloaded and ITS1 sequences were extracted from them using ITSx^60^. These sequences were linked to ASVs from the Global Mycobiome dataset^61^ using blastn (percentage of identity=100%). These ASVs were detected in 780 of 3,194 soil samples (relative abundance >0). The latitude, longitude and biome types of sites at which ASVs were detected were then analysed. We assessed biome enrichment in soils with *P. cucumerina* by comparing observed biome counts to 99,999 random selections of 780 soils using a two-sided Fisher’s exact test, using the function ‘stats.fisher_exact(alternative=’two-sided’)’ in SciPy (1.5.3)^58^.

### Assembly of a culture collection of *Plectosphaerella* isolates

We previously isolated 7 strains of *P. cucumerina* and 1 strain of *P. plurivora* from surface-sterilized *A. thaliana* roots sampled across Europe^6^. We imported 64 additional strains from 17 collaborators or culture collections, sampled from different hosts and environments across different continents (ee Supplementary Data 4 for details).

### Recolonization experiments with *Plectosphaerella* isolates

We performed plant recolonization experiments with individual *Plectosphaerella* strains in an agar-based system. Fungi were revived from 30% glycerol stocks and cultivated for 10–14 days on oatmeal medium, prepared by crushing 5 g of oatmeal in liquid nitrogen with a mortar and pestle until obtaining a powder, which was boiled for 30 min in 200 ml of water on a heating plate at 180 °C, then supplemented with 4 g of Difco agar (214530). Sterile double-distilled water (DW) was poured on the grown mycelium, collected, filtered on Miracloth, then centrifuged at 4,000 × *g* for 10 min to concentrate spores. Spores were counted on a Malassez cell, and their concentration was adjusted to 10^7^ spores per ml in 25% glycerol. These stock solutions were flash frozen in liquid nitrogen, then kept at −80 °C. *A. thaliana* Col-0 seeds were sterilized for 15 min in 70% ethanol, then 5 min in 8% sodium hypochlorite. They were then washed six times in DW and one time in 10 mM MgCl_2_, then kept for 5 days at 4 °C in the dark. Culture medium was prepared by mixing 2.2 g Murashige and Skooge (Duchefa Biochemie, M0222) and 0.5 g MES buffer (Roth, 4256.5) powders in 1 l of double-distilled water. Then, pH was adjusted to 5.7, and 10 g of Difco agar (214530, 1% final concentration) was added. After autoclaving, 120 × 120 mm Petri dishes were each filled with 65 ml of medium. The top 2 cm of agar were cut out, and 7 seeds were deposited where the cut was made. Plates were sealed with micropore tape and their bottoms were wrapped (up to the seeds level) in paper to hide roots from the light. Plants were grown for 10 days at 21 °C (10 h with light intensity 4 at 19 °C and 14 h in the dark) in growth chambers. After 10 days, the four biggest plants in each plate were kept. A spore inoculum was made from 5 μl of defrosted spore stock solution and 995 μl of DW. This mixture was centrifuged at 9,000 *g* for 10 min. The top 950 μl were replaced with fresh DW to remove glycerol. The spore concentration was adjusted to 500 spores per 10 μl. Seedlings (10-day-old) were inoculated by pipetting down 10 μl of inoculum (500 spores) on their primary roots. Plates were then resealed and put back in growth chambers, with the settings mentioned above. After 28 more days in culture, plants were phenotyped by measuring their shoot fresh weight.

### Fungal infection assay with 12 different accessions of *A. thaliana*

*P. cucumerina* 0016 (P16) and 0068 (P68) were stored as mycelial fragments in 30% (v/v) glycerol at −80 °C. Before experiments, individual strains were cultivated on potato glucose agar (PGA, Sigma-Aldrich) plates for 2 weeks. Approximately 50 mg of mycelium was collected using sterile pipette tips and transferred into 1 ml 10 mM MgCl_2_ containing one stainless‑steel bead (Ø 2.85–3.45 mm, Carl Roth) and 10 stainless‑steel beads (Ø 0.75–1 mm, Carl Roth). Samples were homogenized using a paint shaker (SK450, Fast & Fluid Management) for 10 min. The resulting mycelial suspension was adjusted to a final concentration of 5 mg ml^−1^ in 10 mM MgCl_2_ for all experiments. Seeds of *A. thaliana* accessions Van-0, Se-0, Bd-0, Col-0, Ct-1, Mh-0, Ws-2, Ull2-3, Kin-0 and RRS-7 were surface sterilized as described above, stratified and germinated for 7 days under the growth conditions shown above. On the day of inoculation, seedlings were inoculated by applying freshly crushed mycelium (5 mg ml^−1^ in 10 mM MgCl_2_) directly onto the roots, delivering 50 µg of mycelium per plant and then incubating for 15–20 min. Control plants were treated with 10 mM MgCl_2_ alone (Mock condition). Following inoculation, seedlings were transferred to fresh half-strength Murashige and Skoog (MS) agar square plates with the top 2 cm removed, sealed and incubated in the growth chamber. After 15 dpi, SFW was determined. For each accession, SFWs were normalized to the corresponding mock-treated controls before statistical analysis.

### Biolog plate experiment

Mycelia of individual *Plectosphaerella* isolates (*n* = 72) were harvested using sterile tips and the samples were homogenized using the protocol given above. The resulting fungal suspensions were diluted to a final biomass concentration of 0.2 mg ml^−1^ in a 1:1 mixture of Biolog FF and IFO inoculating fluids (Biolog), supplemented with 1% Dye Mix A (Biolog) to enable colorimetric detection. For each isolate, 100 µl of the inoculum was loaded into Biolog PM1 and PM2A MicroPlates, which together contain 190 distinct carbon sources. Plates were incubated at 25 °C without shaking and respiration was monitored every 20 min for 5 days using the OmniLog instrument (Biolog), which records OmniLog Respiration Units (ORU). The area under the curve (AUC) of ORU values was calculated for each well over the 5-day period and log transformed (log AUC) for normalization before data visualization and statistical analysis.

### High-temperature tolerance assays

After growing the 72 *Plectosphaerella* isolates in PGA medium for 7 days, two mycelial discs (0.5 mm in diameter) were excised from the actively growing margins of each colony using the wide end of sterile pipette tips. Each disc was placed upside down onto fresh PGA plates and incubated for 7 days at either 25 °C or 30 °C. After incubation, plates were photographed and the mycelial growth area was quantified using ImageJ. The experiment was conducted with 3 biological replicates. High-temperature tolerance was assessed by calculating the difference in mycelial area between growth at 25 °C and that at 30 °C within each replicate.

### Genome sequencing

The genomes of P16 and P117 were previously sequenced with the PacBio technology and assembled^5^ (GenBank PRJNA371204 and PRJNA570880). We used the previously published genome assembly JAGPXD000000000 for P16 and an improved version of JAGSXJ000000000 for P117 (Mycocosm^62^, identifier Plecucu2). For P30, we used a genome assembled from Illumina short reads (Mycocosm, identifier Plecu1). Other genomes were newly sequenced with PacBio sequencing. Individual strains were revived on potato growth agar medium (Roth, rCP74.1) and cultured for 7 days. Six strains (P43, P143, P340, P421, P455, P612) were sequenced with PacBio CLR technology. Their genomic (g)DNA was extracted according to a previously introduced cetyltrimethylammonium bromide (CTAB) protocol^21^. RNA of these strains was sequenced with Illumina short reads to support gene prediction in the assemblies. All the other strains (*n* = 63) were sequenced with PacBio CCS technology (HiFi reads). Their gDNA was extracted with the same CTAB-based protocol^21^, without the described purification step of the DNA extracts. Native fungal DNA was found in many cases to be recalcitrant to sequencing. Therefore, PacBio barcoded ultra-low libraries were prepared according to the protocol ‘Procedure Checklist - Preparing HiFi SMRTbell Libraries from Ultra-Low DNA Input’ starting with DNA fragmentation using g-Tubes (Covaris). Typically, 5 libraries were pooled and then sequenced on a Sequel IIe SMRT cell with 8 million ZMWs with Binding kit 2.0 or Binding kit 2.2 and the Sequel II Sequencing Kit 2.0 for 30 h. If necessary, gDNA or final libraries were additionally size selected on a Blue Pippin (Sage Sciences) device to remove smaller DNA fragments. After sequencing, the HiFi data were demultiplexed with SMRTlink10.0, and adapter sequences were trimmed away with cutadapt (v.3.5)^63^.

### Genome assembly and gene annotation

Genomes were assembled and polished from PacBio reads using Flye (v.2.9-b1768)^64^ with the parameter *–*genome-size 40 m’, given the size of previously sequenced *P. cucumerina* genomes^5,19^. The parameter ‘–pacbio-hifi’ was used for genomes sequenced with PacBio CCS, and ‘–pacbio-raw’ was used for genomes sequenced with PacBio CLR. Gene prediction was carried out in all the genome assemblies except for P16 and P117, for which we used the gene predictions from the JGI annotation pipeline^62^. We used FGENESH (v.8.0.0)^65^ with similarity matrix *Torrubiella hemipterigena* and the parameters ‘-skip bad prom -skip bad term’. CAZyme-encoding genes were annotated using dbcan (v.4.0.0)^66^. Candidate effector-encoding genes were annotated by first predicting secreted proteins with SignalP (6.0)^67^, then using EffectorP (v.3.0)^68^ (in fungal mode) on predicted secreted proteins. To annotate protease-encoding genes, the annotation tool emapper (v.2.1.5)^69^ based on eggNOG orthology data^70^ was run using DIAMOND as a similarity search algorithm^71^, then we considered as proteases all the proteins linked by emapper to a PFAM identifier associated to a MEROPS family^72^. Transposons were annotated in genome assemblies using reasonaTE (v.1.0)^73^ with the parameters ‘–mode annotate -tool all’.

### Comparative genomics

OrthoFinder (v.2.5.4)^74^ (parameter -S blast) was used to predict orthology among total sets of proteins. Orthogroups were classified by conservation within *P. cucumerina* genomes: ‘core’ in all genomes, ‘broadly conserved’ in at least 75%, ‘low conservation’ in fewer than 20%, ‘subspecies-associated’ if present in over 20% of one subspecies but absent from the other and ‘sparsely distributed’ otherwise. We tested for overrepresentation of these conservation levels among orthogroups encoding CAZymes, effectors and proteases with Fisher’s exact test (function ‘stats.fisher_exact(alternative = ‘two-sided’)’ in Scipy^58^). Principal coordinates analysis (PCoA) were performed by counting the number of annotated genes per family (CAZy and MEROPS families for CAZymes and proteases, respectively, effector orthogroups and complete sets of orthogroups), then calculating pairwise Jaccard distances between strains using the R package Vegan v.2.6-4 (https://github.com/jarioksa/vegan) and its function ‘vegdist(method = ’jaccard’)’. PCoA plots were then reconstructed from these matrices using the function ‘prcomp’ in R. PERMANOVA tests were computed to test for effects of strain phylogeny and the host of isolation on gene catalogues, using the function ‘adonis2(JaccardMatrix~PhylogeneticGroup+HostOfIsolation, permutations=9999)’. Pairwise comparisons between groups were computed with the function ‘pairwise.perm.manova()’ in the R package RVAideMemoire v.0.9-83-12, with default Benjamini–Hochberg correction applied on *P* values.

### Phylogeny reconstruction

Four different methods were used to reconstruct the genome-based phylogeny of our *Plectosphaerella* collection. Two trees were based on 5,466 single-copy orthologues, identified with OrthoFinder^74^. Their sequences were aligned independently using MAFFT (v.7.407)^75^ (parameter –auto), then trimmed with Trimal (v.1.4.rev22)^76^ (default settings). The IQ-TREE (v.2.1.1)^77^ module ModelFinder^78^ identified JTT + F + I + G4 as the most suited maximum-likelihood model for these trimmed alignments. This model was used to reconstruct phylogenies with IQ-TREE and RAxML-NG (v.1.1.0)^79^, following previous alignment concatenation using AMAS^80^ in the case of RAxML-NG. In addition, we reconstructed phylogenies with two different coalescent approaches: the OrthoFinder-associated method STAG^74^ which built individual gene family trees (*n* = 13,694) then reconstructed a coalescent species, and Astral (v.5.7.1)^81^ (default parameters) which we used to reconstruct a coalescent species tree from all the single-copy orthologue gene trees generated by OrthoFinder (*n* = 5,466). The IQ-TREE-generated phylogenetic tree was used as a reference in this study and is presented on multiple figures.

### Multihost recolonization experiments with *P. cucumerina*

Dicot seeds were sterilized and stratified using the steps described above. Barley seeds were sterilized for 1 h in 6% sodium hypochlorite, followed by five washes in sterile double-distilled water. Arabidopsis and tomato seedlings were grown for 14 days and barley seedlings were grown for 7 days in ½ MS media (without carbon source, pH 5.7) in the growth chamber with the default conditions used above. Root inoculation with *P. cucumerina* 0016 and two other isolates (P11, P45) was carried out by harvesting spores from mycelium grown for 8 days on PGA using sterile double-distilled water. Harvested spores were filtered through Miracloth (Merck, 475855-1R), centrifuged for 5 min at 600 × *g* (Eppendorf centrifuge 5810R), and pellets resuspended in 1 ml of sterile double-distilled water. Spores were counted using a haemocytometer and the concentration was adjusted to 2.5 × 10^6^ spores per ml. These spores were inoculated onto the roots of the 3 plant seedlings. After 30 min of incubation, the seedlings were transferred to sterile Magenta boxes containing 40 g of sterile vermiculite with 95 ml of ½ MS media (without carbon source, pH 5.7). After transferring four plants into this ‘vermiculite system’, the boxes were sealed using Micropore by placing an empty magenta box upside down on top. Plants were grown for 21 dpi in the same growth conditions as described above. As a control condition for RNA-seq experiment, sterile Nucleopore track-etched polyester membranes (‘FungusOnMembrane’) were deposited on a ‘vermiculite system’, then 10-μl drops of fungal spores (2.5 × 10^6^ spores per ml of *P. cucumerina* 0016 in sterile double-distilled water) were placed on each one. The membranes were collected and processed as the root samples of our test condition. After 21 dpi, the roots were harvested and flash frozen (if they were for in planta RNA-seq experiment), and SFW was measured for each plant. For analysis, the plant performance index was calculated as the product of SFW and survival rate of the plants^5^. In further correlation analyses, we used log-transformed PPI normalized to mock controls using Hedges’ *g* method. The harvested roots were stored at −80 °C until further use.

### Quantification of fungal load at the roots

Total DNA was isolated from the root samples (one root per tube) using a modified high-throughput version of the FastDNA SPIN kit for soil (MP Biomedicals)^82^. Fungal colonization of these root samples was then measured by qPCR without previous adjustment of DNA concentrations. For each root sample, two reactions were conducted with one pair of primers ITS1F (5’-CTTGGTCATTTAGAGGAAGTAA-3’) and ITS2 (5’-GCTGCGTTCTTCATCGATGC-3’) which target the fungal ITS1 sequence, and another pair of primers specific to plant samples: UBQ10F (5’-TGTTTCCGTTCCTGTTATCT-3’) and UBQ10R (5-ATGTTCAAGCCATCCTTAGA-3) that target the *Ubiquitin10* Arabidopsis gene, nAP256F (5’-CTACCACTGGTCACTTGATC-3’) and nAP257R (5’-CCAGGCATACTTGAATGACC-3’) that target the *EF1-alpha* tomato gene, UBQF (5’-ACCCTCGCCGACTACAACAT-3’) and UBQR (5-CAGTAGTGGCGGTCGAAGTG-3’) that target the *Ubiquitin* barley gene. Each reaction was performed by mixing 5 μl iQ SYBR Green Supermix with 0.3 μl 10 μM forward primer, 0.3 μl 10 μM reverse primer, 3.4 µl DW and 1 μl template DNA. A BioRad CFX Connect Real-Time system was used with the following programme: 3 min of denaturation at 95 °C, followed by 39 cycles of 15 s at 95 °C, 30 s at 60 °C and 30 s at 72 °C. We then calculated a single colonization index for each sample using the following formula: Index = 2−(*C*_q_(ITS1)/*C*_q_(plant reference gene)), where *C*_q_ denotes the quantification cycle, ITS1 denotes the fungal internal transcribed spacer 1 region, and the plant reference gene was *UBQ10* for Arabidopsis thaliana, *EF1-α* for tomato and *Ubiquitin* for barley^5^.

### Confocal microscopy of root colonization by fungi

Roots of Arabidopsis (6 weeks old), tomato (6 weeks old) and barley (4 weeks old) grown for 21 days in mono-association with *P. cucumerina* 0016 were harvested and conserved in 70% ethanol. During the process, the roots were incubated in 20% potassium hydroxide (KOH) at room temperature with orbital shaking at 60 r.p.m. overnight. At the end of the incubation, the KOH was removed and the roots were rinsed twice with 1× phosphate-buffered saline (PBS) at pH 7.4. They were then double stained with 0.1% calcofluor white (Sigma, 18909) and 25 µg ml^−1^ (for Arabidopsis roots) or 50 µg ml^−1^ (for tomato and barley roots) of wheat germ agglutinin conjugated to fluorophore Biotium CF633 (WGA-CF633) buffered at pH 7.4 in PBS. After 30 min of incubation, samples were then washed thrice in PBS and imaged with a Zeiss Confocal LSM880 confocal laser scanning microscope with the associated software ZEN v.2.3 SP1.

### In planta fungal transcriptome analysis

Total RNA was extracted from the roots of Arabidopsis, tomato and barley colonized by *P. cucumerina* 0016 using Qiagen RNeasy Mini kit (74104). PolyA enrichment was carried out on the RNA extracts, then an RNA-seq library was prepared with the NEBNext Ultra II Directional RNA Library Prep Kit for Illumina (New England Biolabs). Sequencing was then performed in single read mode on a HiSeq 3000 system. RNA-seq reads from the agar system were trimmed using Trimmomatic^83^ v.0.38 and the parameters ‘TRAILING:20 AVGQUAL:20 HEADCROP: 10 MINLEN:100’. For RNA-seq reads from the vermiculite system, the trim-galore v.0.6.5 pipeline was used. Subsequently, these reads were employed for the quantification of transcripts in each sample. A unified transcriptome was constructed by concatenating the transcriptomes of plants and *P. cucumerina* (Plecuc1_GeneCatalog_CDS_20171208 from JGI Mycocosm^5,62^) for various hosts. The indexing for Arabidopsis was based on TAIR11 from JGI-Phytozome, on TMCS v.1.2.1 for tomato, and on Hvulgare_462_r1 for barley. The creation of an index for the concatenated transcriptome was accomplished using Salmon (v.1.1.0)^84^. The quantification of transcripts was performed with Salmon quant to determine the read counts for each sample. These datasets were then imported into R utilizing the tximport (v.1.12.3)^85^ Bioconductor package to compute the transcript per million (TPM) and reads per kilobase per million mapped reads (RPKM) values. DESeq2 (v.1.24.0)^86^ was used for quantification of differential expression of genes (DEGs) and log_2_ fold change (log_2_FC) was calculated using ‘FungusOnMembrane’ as control group. log_2_FC values were corrected by shrinkage with the algorithm in the apeglm v.1.6.0 package. Fungal DEGs obtained on infecting different host conditions were used for further analysis. Subsequently, a matrix was created by incorporating the log_10_ (counts per million, CPM) values for individual samples (columns) along with exclusively the fungal DEGs (rows). Computation of log_10_ CPM values was based on the normalized read counts.

### Statistical analysis of DEGs

Using the computed CPM matrix, the Pearson correlation among samples was determined and was used as the basis for multidimensional scaling (MDS) analysis. MDS was carried out utilizing the distance matrix, and pairwise statistical analyses were conducted using Dim1 and Dim2 for each sample group to assess the significance of impact across conditions, employing the ‘ANOVA–TukeyHSD’ function in R. This matrix was also employed for clustering the DEGs usingthe *k*-means clustering algorithm, and the optimum *k* (ranging from 1 to 100) was determined using the Akaike information criterion (AIC) and the Bayesian information criterion (BIC). A heat map was generated using the log_2_FC to demonstrate expression patterns in comparison to the control. DEGs within the groups were visualized through VennDiagram and heat map separately. Gene annotations were assigned by matching the respective gene/transcript/protein IDs to available annotations in JGI Plecuc1 (*Plectosphaerella cucumerina* RP01 (v.1.0)^5^) that includes Gene Ontology (GO), INTERPRO, EffectorP^68^ and CAZyme^87^ terms associated with the fungal genes. For each functional term, gene set enrichment analysis was performed using Fisher’s exact test. The *P* values were adjusted for each condition using the Benjamini–Hochberg method.

### CAZyme network plots targeting predicted cell wall substrates

The CAZyme database was utilized for CAZyme enrichment analysis and visualization. Substrates were categorized manually on the basis of the class of CW (string parsing ‘pectin|cellulose|lignin|arabinan|galactan|glycan|glucan|xylan’ using tidyverse) components, and redundant substrates were grouped together to simplify visualization. A network graph was created using an adjacency matrix consisting of CAZyme families (rows) and possible substrate families (columns) (CAZyDB vCAZyDB.09242021 from the dbCAN2 repository)^33^. A value of 1 was assigned to CAZyme families targeting potential substrates, with the rest being designated as 0. Using this matrix, a network map was constructed, where the edges indicate the number of enzymes (circle nodes) targeting potential substrates (square nodes). *P* values derived from the enrichment analysis of the corresponding CAZyme families were employed to represent the nodes in the network graphs, and the mean log_2_FC of the CAZymes was employed to illustrate the colour of the edges. By integrating the network structure and the significantly enriched CAZymes, the activation of CW components under specific conditions was visualized using the igraph package in R (v.3.6.1 and v.4.3.1).

### Monosaccharide composition and crystalline cellulose measurement

Cellulose content and matrix polysaccharide and monosaccharide composition of the samples were analysed as previously described^88^. In brief, alcohol insoluble residue (AIR) was prepared from freeze-dried root tissues and split into two samples. One half of the samples was treated with a weak acid (4% sulfuric acid) to release matrix polysaccharide-derived sugars, while the other half was treated first with a strong acid (72% sulfuric acid) to swell cellulose. Then the sulfuric acid concentration was diluted to 4% to yield monosaccharides both derived from cellulose and the matrix polymers. Subtraction of the two values allows for the quantification of crystalline cellulose. Monosaccharides of all fractions were quantified using a Professional IC Vario high-performance anion-exchange chromatography system 1068 (Metrohm) equipped with a CarboPac PA20 column (Thermo Fisher) and an amperometric detector (Metrohm). The used sodium hydroxide gradient was specified as previously described^88^. The lignin content of the AIR samples was determined as previously described^89^.

### GH64 occurrences in the fungal kingdom

The CAZYme-family GH64 was procured from the JGI Mycocosm^62^ and from the CAZyme database^87^. These datasets were merged to preserve the taxonomic categorization for the fungal kingdom members (species level) that were present in the CAZyme database based on the portal ID (genomic identifiers). The number of GH64 occurrences was ascertained by cross-referencing the merged dataset. Finally, the FunGuild database was integrated into the concatenated dataset through species or genus identifiers. Collectively, three tables were merged and summarized using a phylogenetic tree of the fungal kingdom (constructed from the ‘taxonomizr’ R package). The count data for the number of GH64 in genomes (from JGI Mycocosm^62^) and the ecological information (FunGuild^90^) were incorporated into the phylogenetic tree utilizing iTOL (v.6.0)^91^. Due to redundancy in the annotation of ecological niches (GUILD category in the FunGuild repository^90^), we classified those annotations that contain the string ‘endophyte’ as endophyte, while others were classified as ‘non-endophyte.’ Statistical analysis was executed employing the pairwise Wilcoxon test with Bonferroni *P* value adjustment; for enrichment analysis within Ascomycota members (at the class level) and within the ecological group, Fisher’s exact test was performed with *P* value adjustment using Bonferroni correction. Similarly, the enrichment of GH64 in genomes of fungi across diverse lifestyles (saprothophs-S, pathogens-P, endophyte-E and all-SPE) was calculated using Fisher’s exact test followed by *P*-value adjustment using Bonferroni correction.

### Structural analysis of GH64

The structure of the GH64 protein from *P. cucumerina* 0016 was predicted with AlphaFold3 after signal peptide removal with SignalP (v.6.0)^67^. The best predicted model was then used as query for the Foldseek^36^ search (online, accessed December 2024), looking for structural similarity with experimentally resolved protein structures registered in the PDB database. Structure alignment between the GH64 enzyme of *P. cucumerina* 0016 and the best hit (SpGlu64A, protein from *Streptomyces* *pratensis*^37^, PDB code 7VTK) was performed using TM-align^92^, as implemented in iCn3d^93^.

### CRISPR/Cas9-mediated genome editing in *P. cucumerina* 0016

#### Design of synthetic sgRNA

The specific sgRNA oligo consisted of a T7 promoter for in vitro transcription, a transcription-starting G nucleotide, a 20-nt target-specific spacer sequence that determines the genomic target, and the constant sgRNA scaffold sequence. The complete oligo sequence was AAGCTAATACGACTCACTATAG(G)NNNNNNNNNNNNNNNNNNNNGTTTTAGAGCTAGAAATAGCAAG, where N represents the target-specific spacer sequence. sgRNAs (Supplementary Data 9) were designed using the sgRNA design tools CRISPick (https://portals.broadinstitute.org/gppx/crispick/public) and CCTop^94^. An sgRNA with a single, unique genomic hit to the target locus was identified using CCTop, and its on-target score was retrieved from CRISPick.

#### Synthesis of sgRNA

For the preparation for gRNA synthesis, specific and constant gRNA oligos were mixed by combining 1 µl of the specific protospacer oligo (100 pmol stock), 1 µl of the constant gRNA oligo (100 pmol stock) and 8 µl of H_2_O, totalling 10 µl. The programme was: 95 °C for 5 min, cooling from 95 °C to 85 °C at 2 °C s^−1^, and further cooling from 85 °C to 25 °C at −0.1 °C s^−1^, with a total time of ~15 min. A T4 DNA mix was prepared to fill in overhangs, consisting of 2.5 µl dNTPs (10 mM stock), 2 µl NEB buffer 2.1 (10× buffer stock), 5 µl H_2_O and 0.5 µl T4 DNA polymerase (NEB, M0203L), totalling 10 µl. This T4 DNA mix was added to the annealed oligos, and the mixture was incubated at 12 °C for 20 min. The product was purified using a PCR column (Macherey-Nagel NucleoSpin Gel and PCR Clean-up kit) and eluted with 30 µl H_2_O. The concentration was measured with a Nanodrop, ensuring it was ~130 ng µl^−1^, and the band size was verified on a 3% agarose gel, expecting a band of ~120 bp. For RNA synthesis, the HiScribe T7 High Yield RNA Synthesis kit (Neb, E2040S) was used, following the protocol for small RNAs.

#### Purification of sgRNA

For sgRNA purification, the RNA Clean & Concentrator 25 kit (Zymo Research, R1017 and R1018) was used. The eluted samples were immediately stored at −80 °C, with a concentration greater than 1 µg µl^−1^. The integrity of the gRNA was checked using 3–4% agarose gel electrophoresis.

#### Design of the donor DNA

Transgenes (donor DNAs) were designed to include a homology arm on the 5’ site of the Cas9 cutting site (60 bp) (Supplementary Data 9), the promoter Pcpc1, the phleomycin-resistance gene *BleoR*^95^, the transcription terminator of tryptophan synthase (TtrpC) and a homology arm on the 3’ site of the Cas9 cutting site (60 bp). This construct was ordered from the Biocat (https://www.biocat.com/gensynthese) in plasmid pUC57.

#### Amplification of donor DNA using PCR

Primers (Supplementary Data 9; P5–P16 used in pairs) were designed to amplify the donor DNA (*Bleo* cassette with the flanking region, as described) from the vector. The amplification was performed using Phusion DNA polymerase (https://www.thermofisher.com/order/catalog/product/de/en/F-530XL). The PCR reactions were set up according to manufacturer protocol. A 1% agarose gel electrophoresis with a 1 kb ladder (GeneRuler, Thermo Fisher) was run to check the integrity of the DNA, ensuring that a single clean band was obtained. Gel purification was performed using the Macherey-Nagel kit.

#### Protoplast isolation

Spores at a concentration of 1.5 × 10^7^ cells per ml were inoculated into 100 ml of PGB medium. The flask was incubated at 25 °C with orbital shaking at 180 r.p.m. for 20 h. Next day, the mycelia were collected by passing the entire culture through a 40 µm filter, and the residues were washed vigorously using 20 ml of 0.7 M NaCl to completely remove the spores from the mycelia^96^. Mycelia (350 mg) were transferred to 10 ml of enzyme solution (20 mg driselase in 10 ml 0.7 M Nacl) and the mixture was incubated in the enzyme solution for 2 h at 28 °C with orbital agitation at 60 r.p.m. After incubation, the solution was filtered (40 µm filter). The filtrate was centrifuged at 250 × *g* for 5 min, with acceleration and deceleration adjusted to 3 in centrifugation (Eppendorf centrifuge 5810R). The pellet was resuspended in 1 ml of STC (sucrose 20% (w/v), TrisHCl (1 M, pH 8) 10 mM, CaCl_2_ (1 M) 50 mM) buffer. The protoplasts were counted using a haemocytometer, and the volume was adjusted to 5 × 10^6^ cells per ml.

#### Polyethylene glycol-mediated transformation

For the polyethylene glycol-mediated (PEG-mediated) transformation, prebound Cas9–gRNA complex (RNP–ribonucleoprotein complex)^97^ was prepared by mixing Cas9 and sgRNA as follows: 0.5 μl 10× NEBuffer r3.1, 1 μl 20 μM EnGen Spy Cas9 HF (NEB, M0667M) and 1.5 μl sgRNA 2 (~1.5 µg, with the desired amount being 20–40 pmol, or 0.8–1.6 μg for 121 nucleotides). This whole mixture was incubated at 25 °C for 30 min and further kept on ice until use. For protoplast transformation, 200 µl of protoplasts were added to a 50 ml Falcon tube and kept on ice. The prebound Cas9–gRNA complex (~20 pmol) and donor DNA containing 2.5 µg of DNA were added to the same tube and gently mixed, and the total volume kept as low as possible. The tube with mixture was incubated on ice for 30 min. Then, 1.5 ml of PEG–STC (6 g of PEG 8000 in 14 ml of STC buffer) was added to the tube, which was rotated horizontally to softly mix the reagent. This was followed by incubation at room temperature for 15 min. Subsequently, 5 ml of CM media (containing 6 g of yeast extract, 6 g casein acid hydrolysate and 10 g sucrose in 1 l H_2_0) was added and mixed well by horizontal rotation. The tube was incubated in the dark at 25 °C for 18 h before being centrifuged at 3,000 × *g* for 5 min. The pelleted fungi were visible on the side of the tube, and the supernatant was removed. Regenerated protoplasts were resuspended together in 100 μl of DW and the entire solution was plated on square plates containing PGA with 200 µg ml^−1^ *Bleo* antibiotic and incubated in a 23 °C incubator. After 2 days, these colonies were transferred to new plates (PGA with *Bleo*), marked with numbers and allowed to grow for 4–5 days before proceeding with the screening of the mutants. For the control, untransformed protoplasts were plated on PGA and used for screening steps to maintain uniformity for the wild-type samples and transformed colonies, especially in the colony PCR step. To screen and to verify the integration of the *BleoR* gene cassette at the right site, we used external primers that anneal to the genomic sequences immediately outside the 5′ and 3′ homology arms (Supplementary Data 9). For colony PCR, the Phire Plant Direct PCR Master Mix was used (Thermo Fisher, F170S).

### In vitro growth assay

To assess the growth rates of mutant *P. cucumerina* 0016 strains relative to the wild type, an in vitro growth assay was conducted. Spores (8 × 10^4^ spores per ml) were then inoculated into 30 ml PGB and incubated at 23 °C with orbital shaking at 180 r.p.m. for 24 h. After incubation, a 1 ml sample was taken to measure the optical density (OD) at 600 nm with a spectrophotometer. The remaining culture was used to collect and recount spores using a haemocytometer. This experiment included 3 biological replicates. In vitro growth dynamics of *P. cucumerina* 0016 WT and *Δgh64* mutant strains in PGB medium were performed in 96-well plates and measured using a spectrophotometer. For WT and *Δgh64* conditions, 180 µl PGB was dispensed per well, whereas 200 µl PGB was dispensed per well for the mock treatment. The outer wells were filled with water to minimize edge effects. Mycelia were collected from PGA and then washed with water to remove residual agar. Further, they were resuspended in water with the concentration adjusted to 2 mg ml^−1^. Later, they were homogenized using a Precellys 24 TissueLyser (Bertin Technologies) at 6,200 r.p.m. for 2 × 30 s with a 15 s interval. Subsequently, 20 µl of mycelial suspension was added to wells containing 180 µl PGB. Each condition was represented by 6 wells per experiment and 3 independent biological experiments were conducted. OD_600_ was measured at 0, 24, 48, 72 and 96 h using a microplate reader (Tecan, Infinite M Nano). For the agar plate assay, 20 µl of mycelial suspension (2 mg ml^−1^) was placed at the centre of PGA plates for each condition, and mycelial growth was recorded after 10 days. For the both in vitro growth assay and the in vitro growth dynamics experiments, the samples were incubated at 23 °C.

### Hydrolysis of laminarihexaose by *Plectosphaerella* strains

*P. cucumerina* 0016 WT and *Δgh64* mutant strains were grown on PGA for 7 days. Spores were collected from the mycelium, counted using a haemocytometer and adjusted to 1.18 × 10^6^ spores per ml. Laminarihexaose was added as the sole carbon source to a final concentration of 0.1 g l^−1^. The suspensions were then incubated at 28 °C. After 30 min, 500 µl was collected, filtered through a Millex syringe filter (0.22 µm, 4 mm) and heated at 100 °C for 5 min to stop hydrolytic activity. Samples were then stored at −20 °C for further analysis. Control conditions consisted of fungal suspensions incubated without LAM6 (mock condition) and the LAM6 sample collected at 0 h before incubation. All steps were performed under sterile conditions, including 3 biological replicates. To analyse released laminari-oligosaccharides, HPAEC–PAD was performed. The analysis was carried out using a Dionex ICS-6000 system operated with Chromeleon 7 software (v.7.3.1). Separation of the oligosaccharides was conducted at 30 °C on a CarboPac PA200 analytical column (3 mm × 250 mm) fixed to a CarboPac PA200 guard column (3 mm × 50 mm). Elution was achieved using a binary gradient consisting of eluent A (100 mM NaOH) and eluent B (1,000 mM NaOAc in 100 mM NaOH) at a constant flow rate of 0.3 ml min^−1^. A volume of 500 µl of diluted sample (3/100) was injected. For qualitative and quantitative analysis, a 4-point external calibration curve was generated using d-glucose from Sigma-Aldrich, and laminaribiose (O-LAM2, β-1,3-d-(Glc)_2_), laminaritriose (O-LAM3, β-1,3-d-(Glc)_3_), laminaritetraose (O-LAM4, β-1,3-d-(Glc)_4_), laminaripentaose (O-LAM5, β-1,3-d-(Glc)_5_), laminarihexaose (O-LAM6, β-1,3-d-(Glc)_6_) from Megazyme (https://www.megazyme.com).

### Experiments with *C. incanum*

Targeted gene replacement was carried out using *Agrobacterium*-mediated transformation which is based on ref. ^44^. After transformation, colonies were grown on Mathur’s agar (3% w/v) plates supplemented with hygromycin (200 μg ml^−1^), cefotaxime (100 μg ml^−1^) and spectinomycin (100 μg ml^−1^). Colony PCR was performed to validate the success of the gene replacement using primers listed in Supplementary Data 9. Agar plugs from fungal cultures streaked on Mathur’s agar plates were transferred to fresh Mathur’s agar plates and incubated at 25 °C for 7 days under continuous light (~10 μmol m^−2^ s^−1^). Arabidopsis or *Nicotiana benthamiana* seeds were surface sterilized with 70% ethanol for 30 s twice, followed by 6% sodium hypochlorite with 0.01% Triton X for 5 min. After being washed 5 times with DW, seeds were sowed on half-strength MS agar (1.3% w/v) plates without sucrose. Spores from Mathur’s plates were collected by flooding the fungal culture plate with 5 ml DW, washed once in DW, then counted by haemocytometry in a light microscope. Spores were diluted to 5,000 spores per ml before inoculating 3 μl of the spore suspension 3 cm beneath seeds. Plates were incubated vertically in a plant growth chamber at 22 °C under fluorescent light (~100 μmol m^−2^ s^−1^) with a 16-h photoperiod at ~40% humidity. Seedlings were harvested at 24 dpi for shoot fresh weight measurement. Roots were sampled, flash frozen in liquid nitrogen and stored at −80 °C until RNA was extracted using the Nucleospin RNA kit (Macherey-Nagel) according to manufacturer instructions. RNA was immediately used for complementary (c)DNA synthesis, then stored at −80° C. For RT–qPCR, cDNA was synthesized from ~100 ng total RNA using the PrimeScript RT Master Mix (TaKaRa) in a volume of 10 µl following manufacturer instructions. RT–qPCR was performed using the THUNDERBIRD Next SYBR qPCR Mix (TOYOBO) with 250 nM primers using the CFX Opus 384 Real-Time PCR System (BIORAD) in a volume of 10 µl for 48 cycles. Primers are listed in Supplementary Data 9.

### Statistics

The R programming environment (R v.4.4.0) was used for data analysis and visualization (https://www.r-project.org/). Normality of the data was assessed with the Shapiro–Wilk test using the stats package (v.4.4.0) and homogeneity of variances with Levene’s test using the car package (v.3.1-2). Two-sided *t*-test and two-sided non-parametric Wilcox rank-sum test were performed using t.test and wilcox.test in the stats package (v.4.4.0) in R. ANOVA–TukeyHSD tests were conducted using the emmeans package (v.1.10.3). Non-parametric Kruskal–Wallis tests were carried out with the stats package (v.4.4.0), and Dunn’s tests were performed using the FSA (v.0.9.5) and rcompanion packages (v.2.4.36). Reported *P* values were Bonferroni adjusted using stats package v.4.4.0.

### Reporting summary

Further information on research design is available in the Nature Portfolio Reporting Summary linked to this article.

## Supplementary information

Supplementary Information Supplementary Figs. 1–13.

Reporting Summary

Peer Review File

Supplementary Dataset 1 Core fungal ASVs detected in *A. thaliana* roots based on two previous studies.

Supplementary Dataset 2 Sequence identity match between zOTUs and cultured isolates.

Supplementary Dataset 3 Taxonomy assignment of cultured fungal isolates.

Supplementary Dataset 4 *Plectosphaerella* isolates used in this study.

Supplementary Dataset 5 Results of PERMANOVA analysis testing for the effect of *Plectosphaerella* and host-of-isolation phylogenetic groups on gene repertoire compositions.

Supplementary Dataset 6 Fungal RNA-seq data.

Supplementary Dataset 7 Detection of the phleomycin cassette in *P. cucumerina* 0016 genome.

Supplementary Dataset 8 Fungal genomes with single-copy GH64.

Supplementary Dataset 9 Primers used in generating/screening mutant lines of *P. cucumerina* and *C. incanum*.

Supplementary Dataset 10 Raw data for Supplementary Figs. 12f and 13.

Supplementary Dataset 11 Raw data of Supplementary Fig. 13.

## Source data

Source Data Figs. 1–6 and Extended Data Figs. 2–10 Ecological data (fungal detection in natural samples). Phylogenetic tree, plant phenotypic data (following fungal inoculation), statistical source data (PCoA coordinates). Plant PPI data and fungal load data. FungalTranscriptome1, FungalTranscriptome2 (Fungal transcriptome expression data matrix, DEseq2 table and Functional annotation). FungalTranscriptome3, FungalTranscriptome4 (Cazyme annotations and Cazyme substrate-enzyme matrix). Genomic data (gene conservation). Plant phenotypic data (following fungal inoculation). PcInoculation–plant phenotypic data (Different *A. thaliana* ecotypes with P68 and P16 infections). Fungal phenotypic data (growth at different temperatures). Carbon utilization by *Plectosphaerella* isolates. Plant SFW, PPI and survival data, together with the corresponding statistical analyses, for plants inoculated with different *P. cucumerina* strains. FungalTranscriptome1. Cell wall composition analysis, mean, s.d. and *P* values. Hydrolysis of laminarihexose data, calibration standards, and statistical data. Inoculation experiment with *Colleteotrichum* sp. in tobacco, statistical data, mean and s.d. values.

## Data Availability

RNASeq raw data are available at the European Nucleotide Archive (ENA) under the study ID PRJEB89666. The raw genomic data of the *Plectosphaerella* isolates have been deposited in the ENA database under the project IDs PRJEB61839 (P143), PRJEB50298 (P340, P421, P455) and PRJEB90544 (all other isolates). See Supplementary Data 4 for individual ENA sample IDs. The 72 *Plectosphaerella* isolates generated in this study are available from the corresponding author upon reasonable request. Source data are provided with this paper.

## Code availability

Scripts and raw data used to generate figures are available in GitHub at https://github.com/fantin-mesny/Scripts_comparison_of_Plectosphaerella_strains (ref. ^98^) for WGS; https://github.com/arpankbasak/Plecuc1_Repo (ref. ^99^) for RNA-seq, GH64 phylogeny and recolonization experiment on Arabidopsis accessions; and https://github.com/RamSevakRK/Genetic-factors-driving-multi-host-infection-in-a-core-member-of-the-root-mycobiota (ref. ^100^) for all other experiments.
