# Supplementary material for: Carbohydrate-active enzymes from a core root mycobiota member enable infection of multiple plant hosts
Source: Nat Microbiol. 2026 Oct 1;11(11):2975–93. doi: 10.1038/s41564-026-02492-3 (PMC13739743; doi:10.1038/s41564-026-02492-3)
Supplement: Supplementary file 1 [file 41564_2026_2492_MOESM1_ESM.pdf]

nature microbiology

Supplementary information https://doi.org/10.1038/s41564-026-02492-3

Carbohydrate-active enzymes from a core root mycobiota member enable infection of multiple plant hosts

In the format provided by the authors and unedited

Supplementary information for

Carbohydrate-active enzymes from a core root mycobiota member enable infection of multiple plant hosts

Ram-Sevak Raja-Kumar[1,*], Fantin Mesny[1,*], Arpan Kumar Basak[1,2,*], Jacy Newfeld[3], Guillaume Chesneau[1], Frederickson Entila[1], Tak Lee[1], Linda Rigerte[1], Stephanie Carvajal Acevedo[1], Bruno Hüttel[1,4], Pedro W. Crous[5,6,7], Jose G. Maciá-Vicente[8], Helen Stewart[9], Matthew Ryan[9], Ahmad M. Fakhoury[10], Soledad Sacristán[11,12], Meriem Aitouguinane[11,12], Isabelle Batisson[13], Stefano Dumontet[14], Wade H. Elmer[15], Jana Henzelyová[16], Joanna S. Kruszewska[17], Jessica M. Nelson[18], Cara M. Santelli[19], Markus Pauly[20], Antonio Molina[11,12], Kei Hiruma[3], Stéphane Hacquard[1,21#]

[1]Max Planck Institute for Plant Breeding Research, 50829 Cologne, Germany. [2]Malopolska Centre of Biotechnology, Jagiellonian University, Krakow, 30-387 Poland. [3]Department of Life Sciences, Multidisciplinary Sciences, Graduate School of Arts and Sciences, The University of Tokyo, 3-8-1, Komaba, Meguro-ku, Tokyo 153-8902, Japan. [4]Max Planck Genome Centre Cologne, Max Planck Institute for Plant Breeding Research, 50829 Cologne, Germany. [5]Westerdijk Fungal Biodiversity Institute, P.O. Box 85167, 3508 AD Utrecht, The Netherlands. [6]Microbiology, Department of Biology, Faculty of Science, Utrecht University, Padualaan 8, 3584 CT Utrecht, The Netherlands. [7]Department of Genetics, Biochemistry and Microbiology, Forestry and Agricultural Biotechnology Institute (FABI), University of Pretoria, Pretoria, 0002, South Africa. [8]Marine Sciences and Applied Biology, University of Alicante, PO Box 99, 03083 Alicante, Spain. [9]CABI, Silwood Park, Buckhurst Rd, Ascot SL5 7PY, United Kingdom. [10]School of Agricultural Sciences, Southern Illinois University, Carbondale, IL 62901, USA. [11]Centro de Biotecnología y Genómica de Plantas, Universidad Politécnica de Madrid (UPM)-Instituto Nacional de Investigación y Tecnología Agraria y Alimentaria (INIA/CSIC), Campus Montegancedo-UPM, 28223-Pozuelo de Alarcón (Madrid), Spain. [12]Departamento de Biotecnología-Biología Vegetal, Escuela Técnica Superior de Ingeniería Agronómica, Alimentaria y de Biosistemas, Universidad Politécnica de Madrid, 28040-Madrid, Spain. [13]Université Clermont-Auvergne, CNRS, Laboratoire Microorganismes: Génome et Environnement, F-63000 Clermont-Ferrand, France. [14]Department of Sciences and Technologies, University of Napoli 'Parthenope', Centro Direzionale, Isola C4, 80143 Napoli, Italy. [15]Department of Plant Pathology and Ecology, The Connecticut Agricultural Experiment Station, New Haven, Connecticut 06504, USA. [16]Department of Genetics, Institute of Biology and Ecology, Faculty of Science, Pavol Jozef Šafárik University in Košice, Mánesova 23, 04154, Košice, Slovakia. [17]Institute of Biochemistry and Biophysics, Polish Academy of Sciences, Warsaw, Poland. [18]Natural History Museum, University of Oslo, 0562 Oslo, Norway. [19]Department of Earth and Environmental Sciences, University of Minnesota Twin Cities, Minneapolis, MN, USA. [20]Institute of Plant Cell Biology and Biotechnology, Heinrich Heine University Düsseldorf, Düsseldorf, Germany. [21]Cluster of Excellence on Plant Sciences (CEPLAS), Max Planck Institute for Plant Breeding Research, 50829 Cologne, Germany.

* These authors contributed equally
[#]Corresponding author: S. Hacquard (hacquard@mpipz.mpg.de).

This PDF file includes the following Supplementary Information:
Supplementary Figures 1 to 13

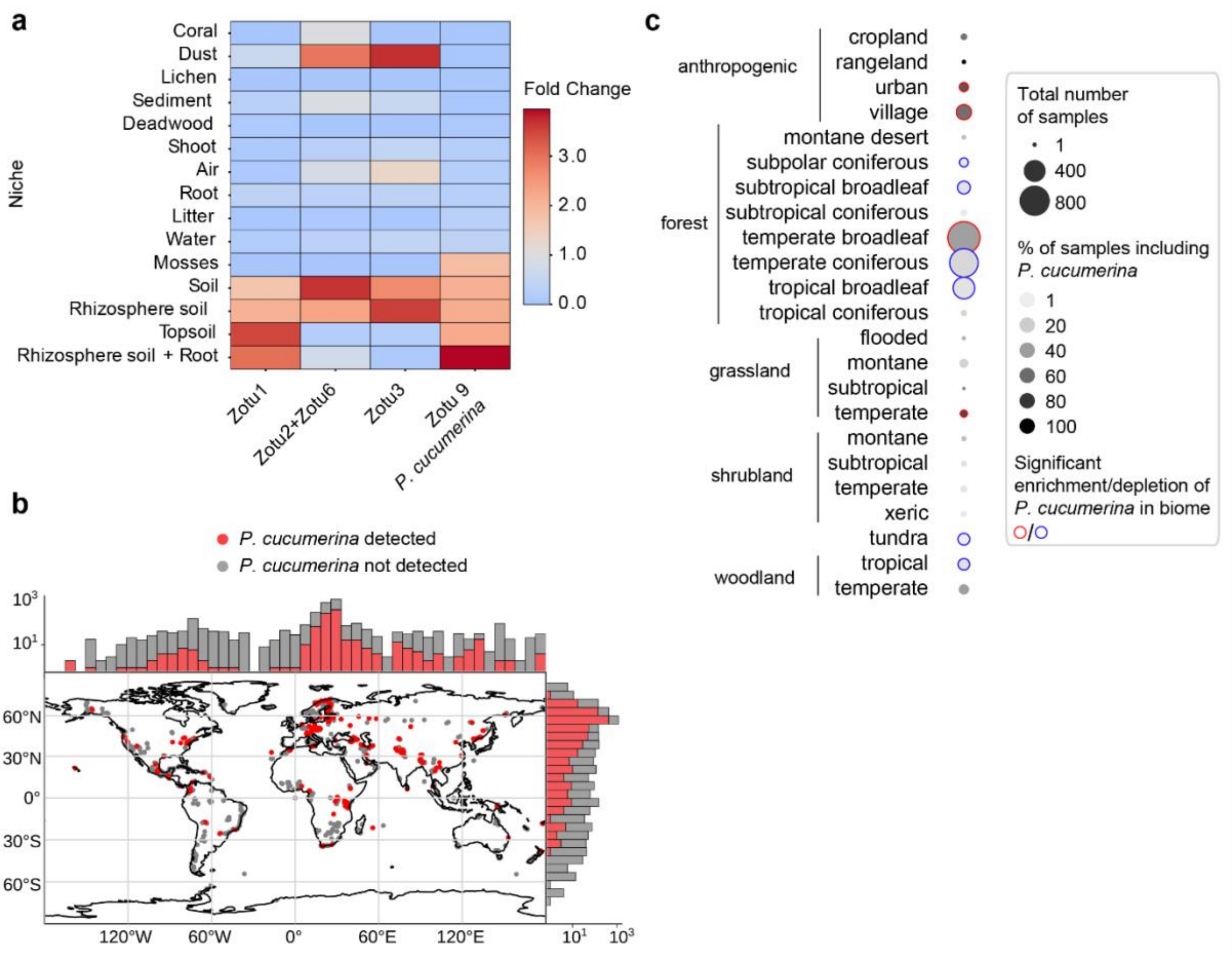

Supplementary Fig. 1: *P. cucumerina* is a ubiquitous soil-borne fungus **a,** Heatmap illustrating, for each core mycobiome Zotu-associated species, fold-change values corresponding to the proportion of GlobalFungi samples from a specific niche where the species was detected, relative to the proportion of samples from all other niches in which the species was detected. Each Zotu was linked to a single species hypothesis in GlobalFungi (Zotu1: SH0736260.10FU; Zotu2 and Zotu6: SH0730983.10FU; Zotu3: SH0730975.10FU; Zotu9: SH1002406.10FU). **b,** Detection of *P. cucumerina* (presence of curated ITS1 sequences[1]) in soil samples originating from all continents (n = 3,194 samples based on Global Soil Mycobiome dataset[2]). **c,** Soil biome enrichment/depletion in samples containing *P. cucumerina*, as tested by Fisher's exact test (see Methods) using the Global Soil Mycobiome dataset and the metadata associated to soil samples.

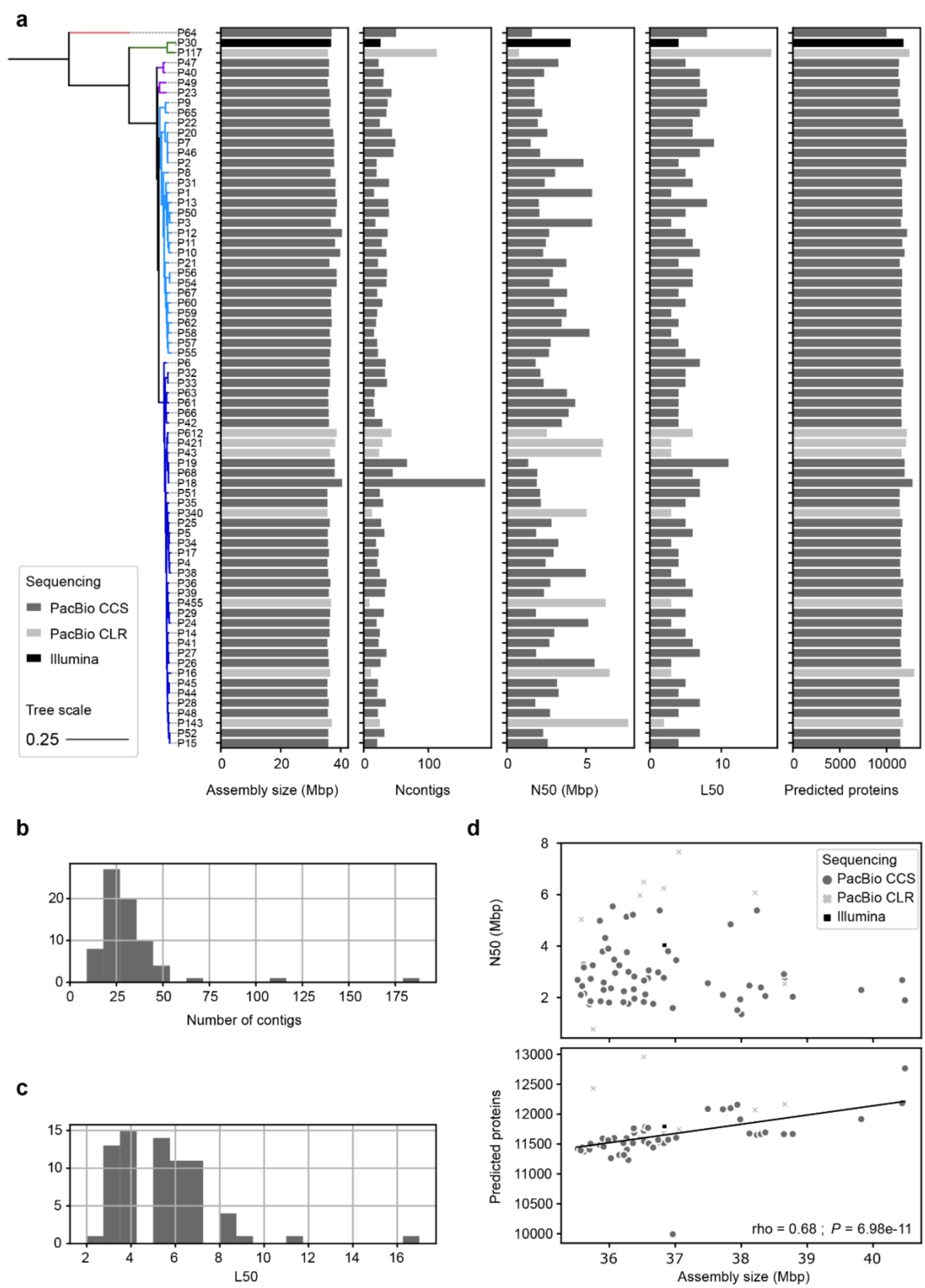

Supplementary Fig. 2: Assembly size and quality for 72 *Plectosphaerella* genomes. a, For each genome in our genomic dataset, the following values are plotted: assembly size, number of contigs in the assembly, N50 and L50 values, number of predicted genes in the assembly. These values are represented along the *Plectosphaerella* collection phylogeny reconstructed with IQ-TREE (See Methods). Note that isolates P68 and P16 correspond respectively to *P. cucumerina* BMM (PcBMM) and *P. cucumerina* 0016 (see this work). **b,**

Distribution of the number of contigs in the genomic data set (n = 72, one value per genome assembly). **c,** Distribution of the L50 values in the genomic data set (n = 72). **d,** N50 values (top) and numbers of predicted genes (bottom), plotted in function of assembly size. The number of predicted genes is significantly correlated to the assembly size according to Spearman's rank correlation.

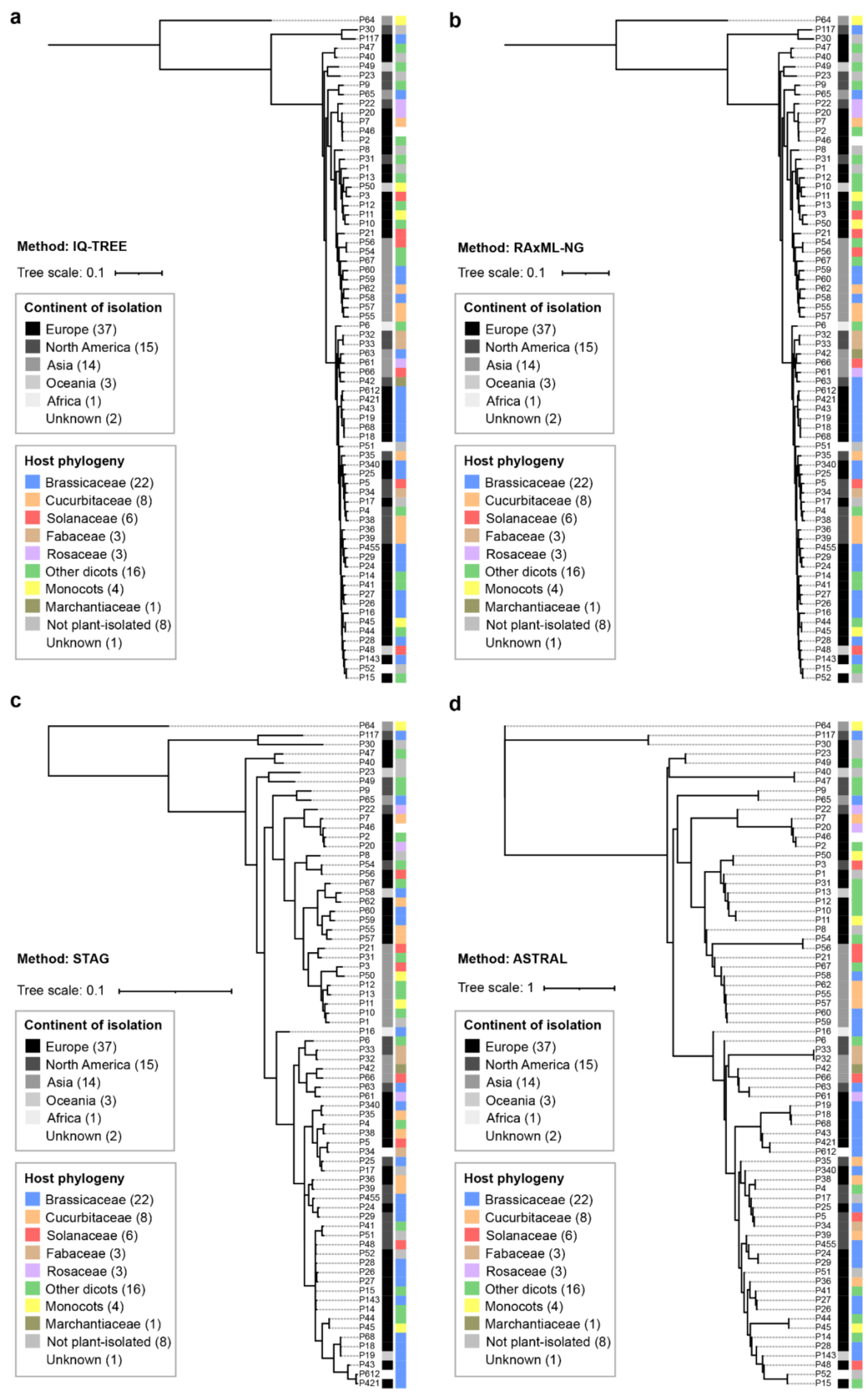
a
Method: IQ-TREE
Tree scale: 0.1
Continent of isolation
Europe (37)
North America (15)
Asia (14)
Oceania (3)
Africa (1)
Unknown (2)
Host phylogeny
Brassicaceae (22)
Cucurbitaceae (8)
Solanaceae (6)
Fabaceae (3)
Rosaceae (3)
Other dicots (16)
Monocots (4)
Marchantiaceae (1)
Not plant-isolated (8)
Unknown (1)
b
Method: RAxML-NG
Tree scale: 0.1
Continent of isolation
Europe (37)
North America (15)
Asia (14)
Oceania (3)
Africa (1)
Unknown (2)
Host phylogeny
Brassicaceae (22)
Cucurbitaceae (8)
Solanaceae (6)
Fabaceae (3)
Rosaceae (3)
Other dicots (16)
Monocots (4)
Marchantiaceae (1)
Not plant-isolated (8)
Unknown (1)
c
Method: STAG
Tree scale: 0.1
Continent of isolation
Europe (37)
North America (15)
Asia (14)
Oceania (3)
Africa (1)
Unknown (2)
Host phylogeny
Brassicaceae (22)
Cucurbitaceae (8)
Solanaceae (6)
Fabaceae (3)
Rosaceae (3)
Other dicots (16)
Monocots (4)
Marchantiaceae (1)
Not plant-isolated (8)
Unknown (1)
d
Method: ASTRAL
Tree scale: 1
Continent of isolation
Europe (37)
North America (15)
Asia (14)
Oceania (3)
Africa (1)
Unknown (2)
Host phylogeny
Brassicaceae (22)
Cucurbitaceae (8)
Solanaceae (6)
Fabaceae (3)
Rosaceae (3)
Other dicots (16)
Monocots (4)
Marchantiaceae (1)
Not plant-isolated (8)
Unknown (1)

Supplementary Fig. 3: Phylogenomic trees of the 72 *Plectosphaerella* strains, reconstructed with four different methods. **a.** Phylogenetic tree reconstructed with IQ-TREE[3] (model *JTT+F+I+G4*, as defined by ModelFinder[4]) from the trimmed concatenated alignments of 5,466 single copy orthologs identified by orthology prediction with OrthoFinder[5]. **b.** Phylogenetic tree reconstructed with RAxML-NG[6] (model *JTT+F+I+G4*, as defined by ModelFinder) from the trimmed concatenated alignments of 5,466 single copy orthologs identified by orthology prediction with OrthoFinder. **c.** Coalescent phylogenetic tree reconstructed from total gene family trees generated by OrthoFinder, with built-in method STAG[7]. **d.** Coalescent phylogenetic tree reconstructed with ASTRAL[8] from the OrthoFinder-reconstructed gene family trees of 5,466 single copy orthologs. See Methods for further details.

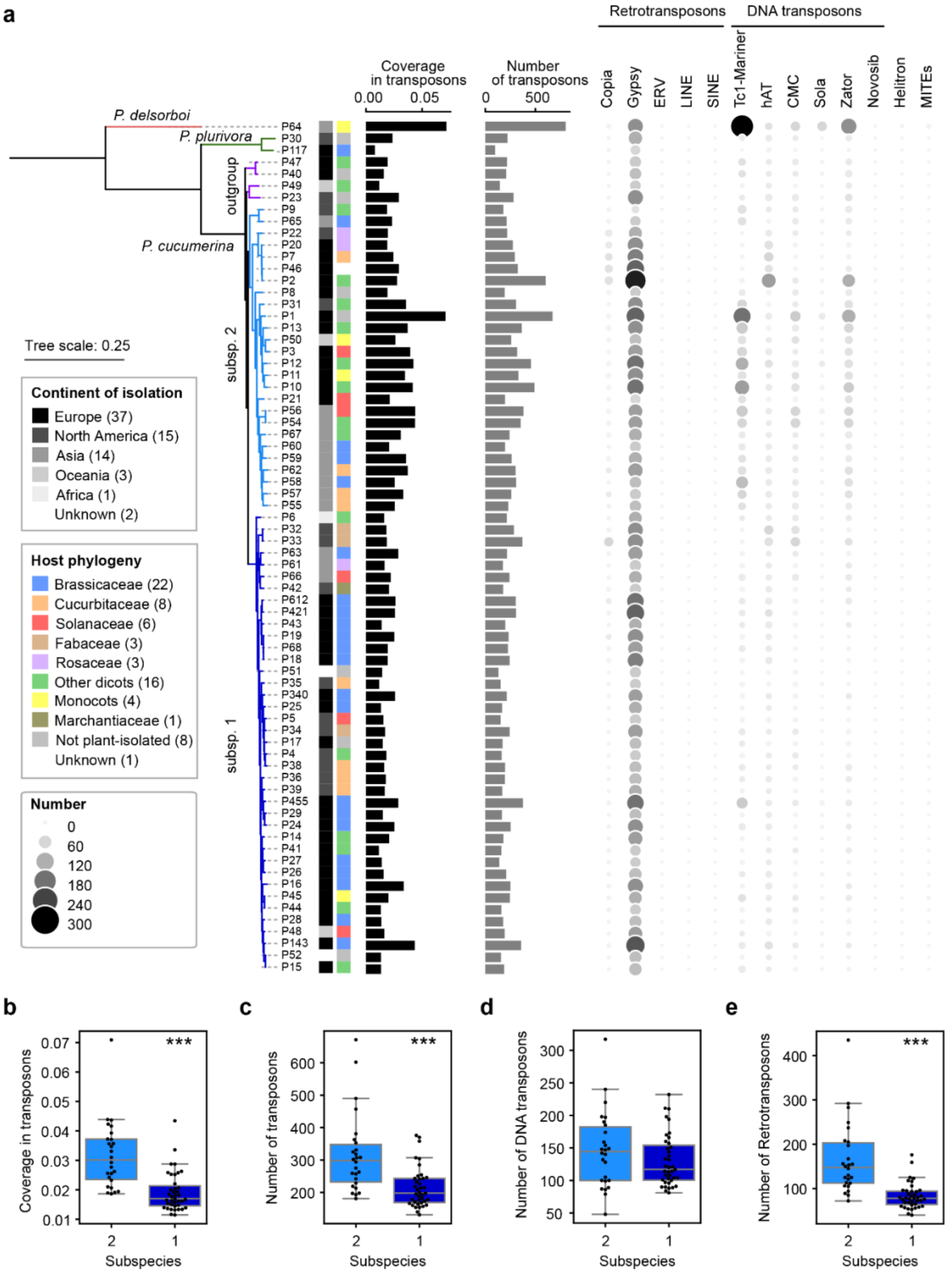

Supplementary Fig. 4: Transposon annotation of the 72 *Plectosphaerella* genome assemblies. a, Results of transposon annotation with the pipeline TransposonUltimate[9]. Along the *Plectosphaerella* collection phylogeny, barplots show the percentage of each genome covered by transposons ("coverage") and the total numbers of transposons annotated. On the right, a bubble plot depicts the number of transposons annotated per family. **b,** Boxplot displaying the difference of *P. cucumerina* genomes in subspecies 1 and 2 in coverage in transposons. Mann-Whitney test revealed a significant difference (statistic = 134.0, *P* =

6.10x10^{-7}). **c,** Boxplot displaying the difference of *P. cucumerina* genomes in subspecies 1 and 2 in transposon number. Mann-Whitney test revealed a significant difference (statistic = 194.5, P = 2.94x10^{-5}). **d,** Boxplot displaying the difference of *P. cucumerina* genomes in subspecies 1 and 2 in DNA transposon number. Mann-Whitney test revealed no significant difference (statistic = 437.0, P = 0.35). **e,** Boxplot displaying the difference of *P. cucumerina* genomes in subspecies 1 and 2 in retrotransposon number. Mann-Whitney test revealed no significant difference (statistic = 108.0, P = 9.46x10^{-8}).

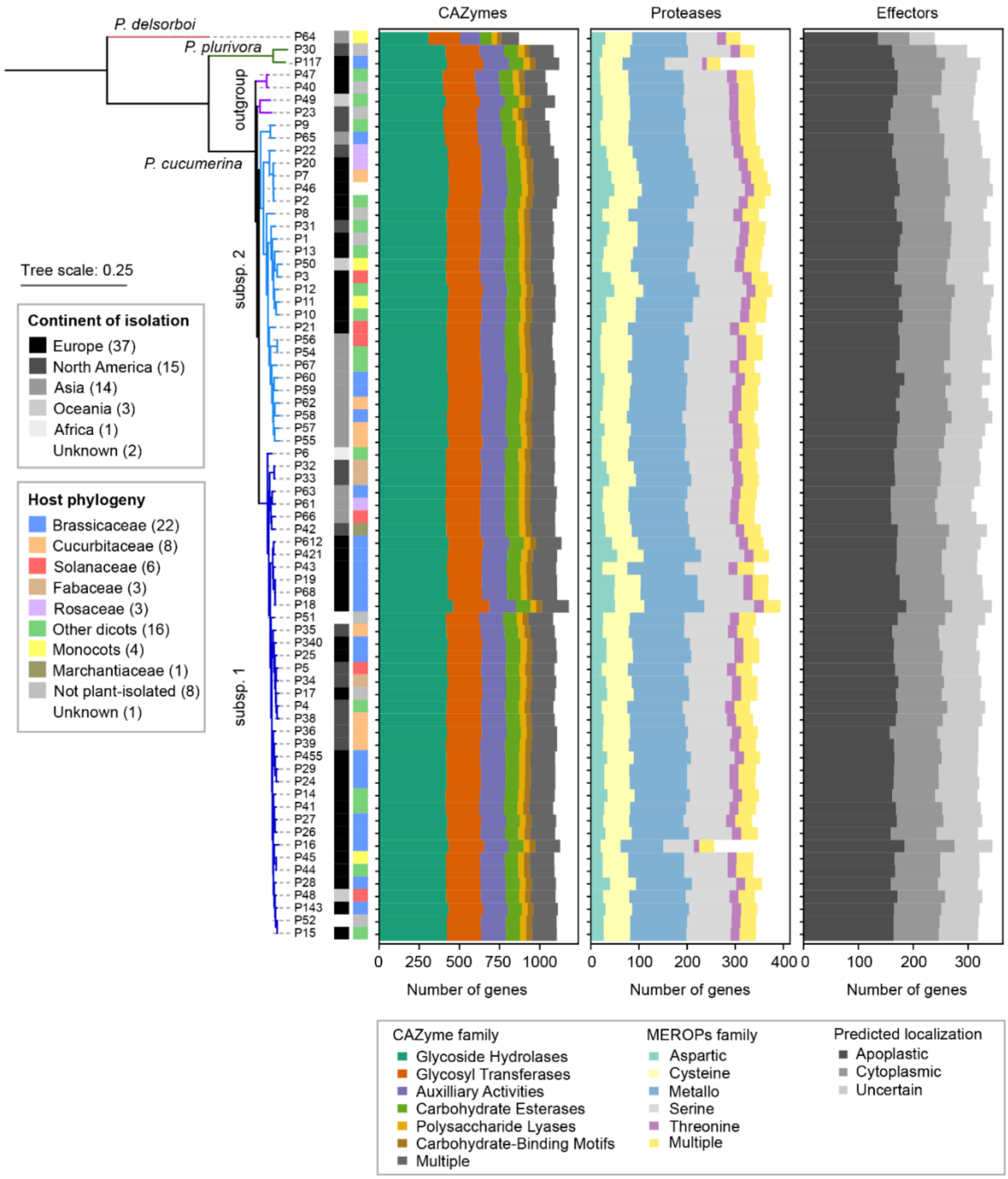

Supplementary Fig. 5: Genome annotation of the 72 *Plectosphaerella* isolates in CAZyme-, protease- and effector-encoding genes. Number of CAZyme- (left), protease- (center) and effector- (right) encoding genes annotated in each of the 72 *Plectosphaerella* genomes, using methods dbcan[10], emapper[11] and EffectorP[12], respectively. Different colours divide the barplots to highlight different functional families.

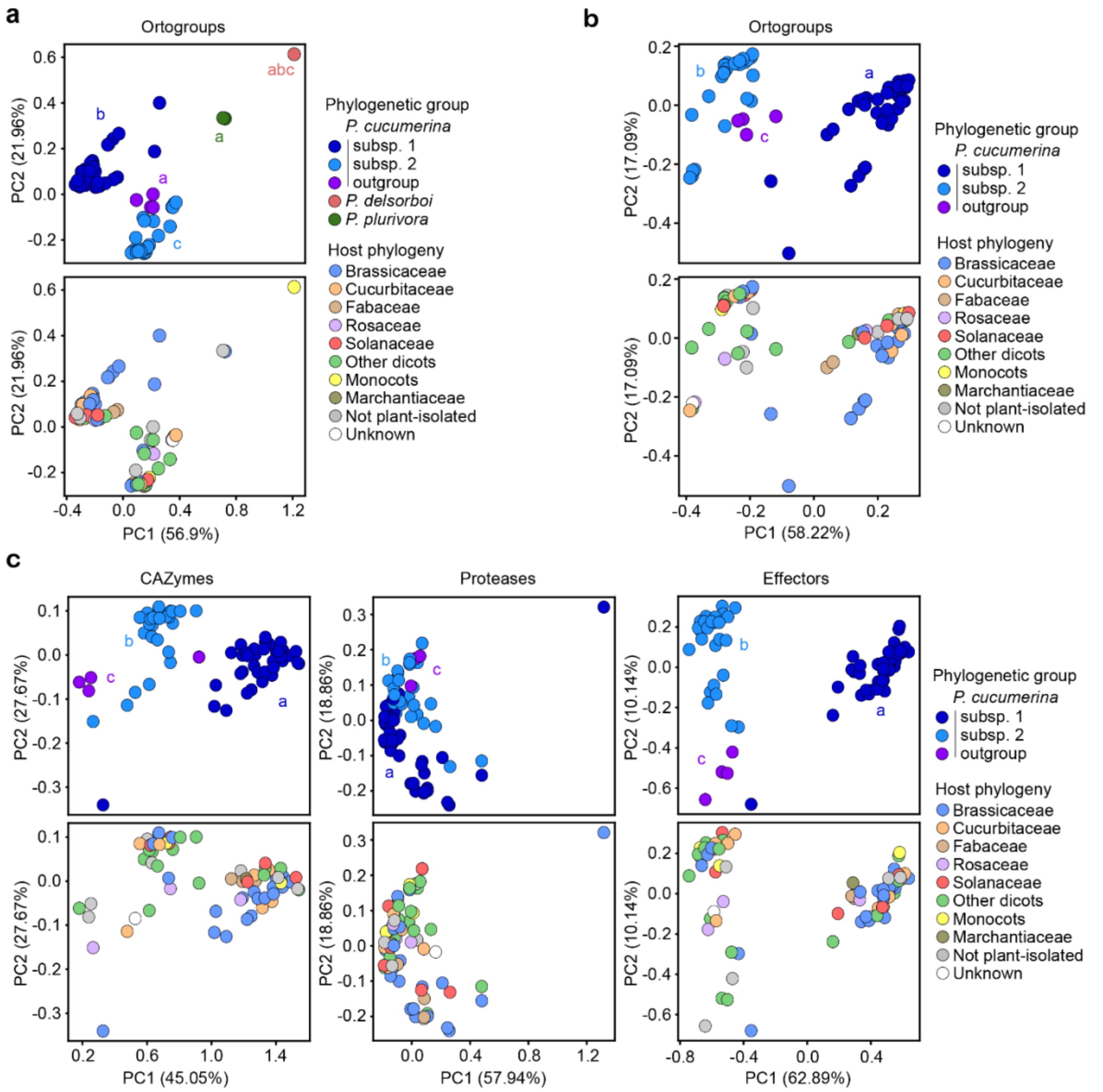

Supplementary Fig. 6: Effect of phylogeny and the host of isolation on inter-strain differences in gene family repertoire. a,b, Principal Coordinate Analysis profiling inter-strain differences in total gene family repertoires (n = 15,269 orthogroups, OrthoFinder; Jaccard distances on orthogroup copy numbers). This PCoA is coloured according to strain phylogenetic group (top) or host phylogeny (bottom). It was performed on total *Plectosphaerella* genomes (3 species, n = 72, panel **a**) and on *P. cucumerina* genomes only (n = 69, panel **b**). **c,** Principal Coordinate Analyses (Jaccard distances calculated on family copy numbers) profiling inter-strain differences in genomic repertoires of CAZymes, proteases and candidate effectors across 69 *P. cucumerina* isolates. The PCoA plots are colour-coded according to *P. cucumerina* phylogenetic group (top) or family names of the hosts of isolation (bottom). R^2 and *P* resulting from PERMANOVA tests assessing the effect of these two categories on orthogroup compositions and the three genomic repertoires are listed in **Supplementary Table 5**. Letters on PCoA plots indicate significant differences between groups according to post-hoc pairwise permutation MANOVAs (adj. $P < 0.05$).

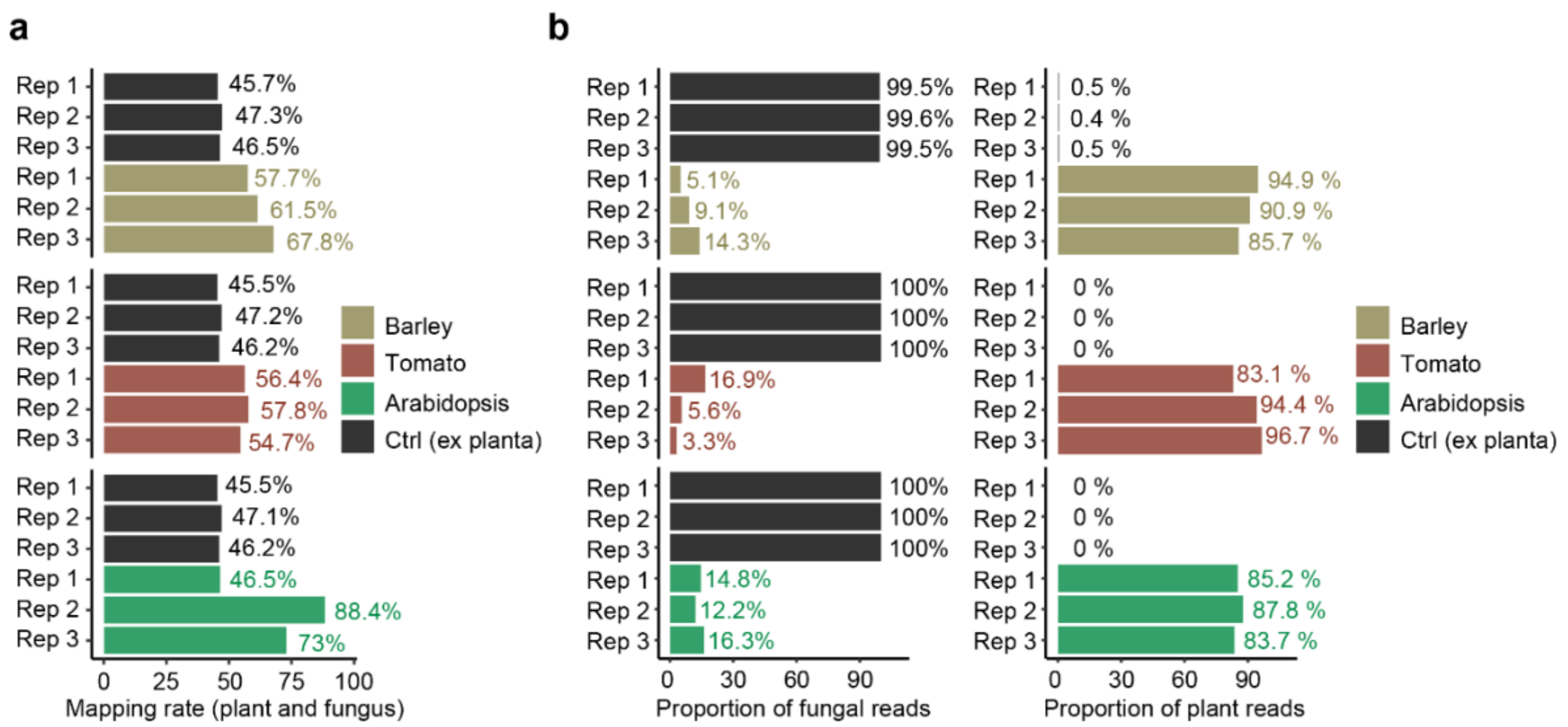

Supplementary Fig. 7: Mapping rates of plant and fungal RNAseq reads against corresponding reference genomes. a, The bar chart depicts for each sample the proportion of sequencing reads (i.e., mapping rate) that aligned against the concatenated reference transcriptome of the plant host (either *A. thaliana* Col-0 - TAIR11[13], Micro-Tom - TMCSv1.2.1[14], barley - Hvulgare_462_r1[15] and *P. cucumerina* 0016 utilizing Salmon[16]. **b**, The proportion of fungal reads (left) and plant reads (right) mapped against the corresponding reference transcriptomes. The colours represent the mapping rate of fungal and host reads in roots of barley (khaki), tomato (deep red) and Arabidopsis (green) and of fungal reads in the absence of the host (Ctrl, black).

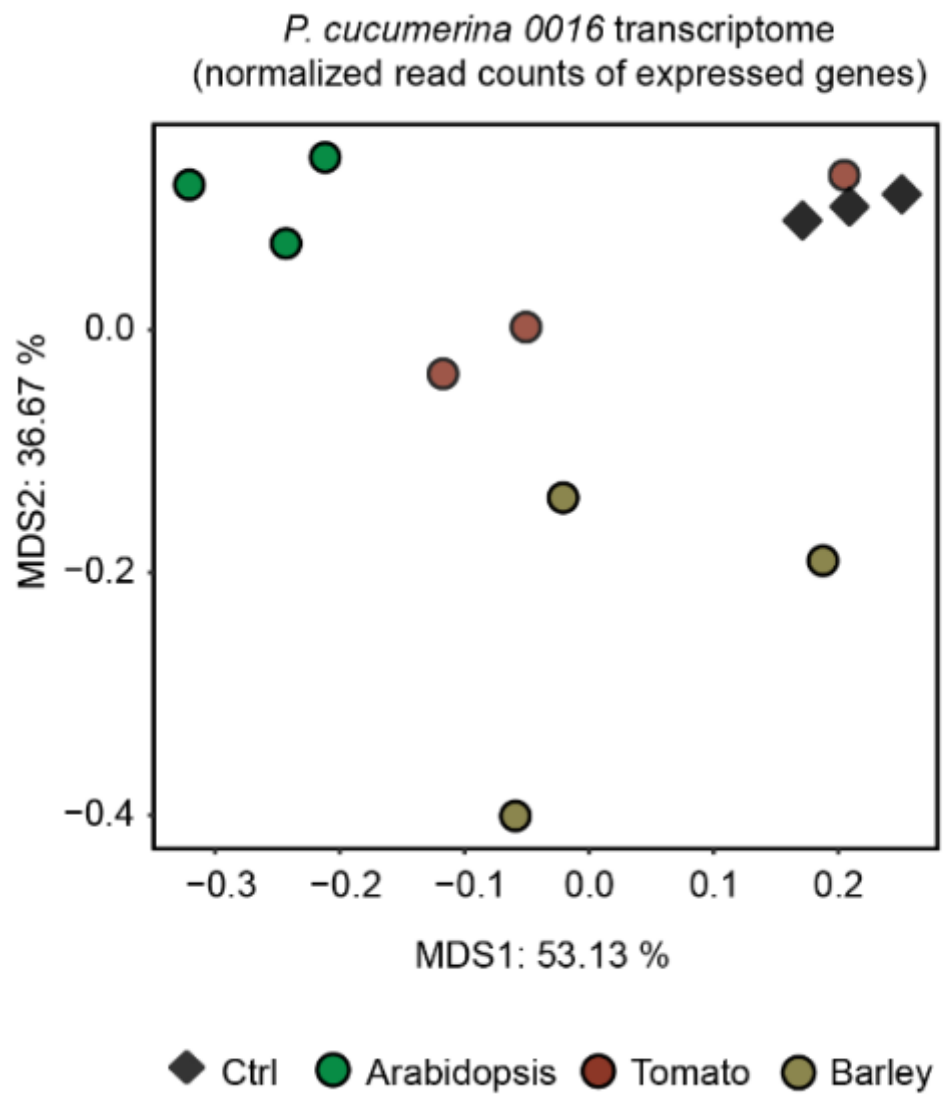

Supplementary Fig. 8. Multidimensional Scaling (MDS) plot of Pearson distances computed on normalized read counts of expressed fungal genes. Fungal transcriptome in roots of Arabidopsis (Green), tomato (Red), and barley (Khaki), as well as in control membrane (Ctrl, *ex planta*, Black). Data points indicate three biological replicates for each condition.

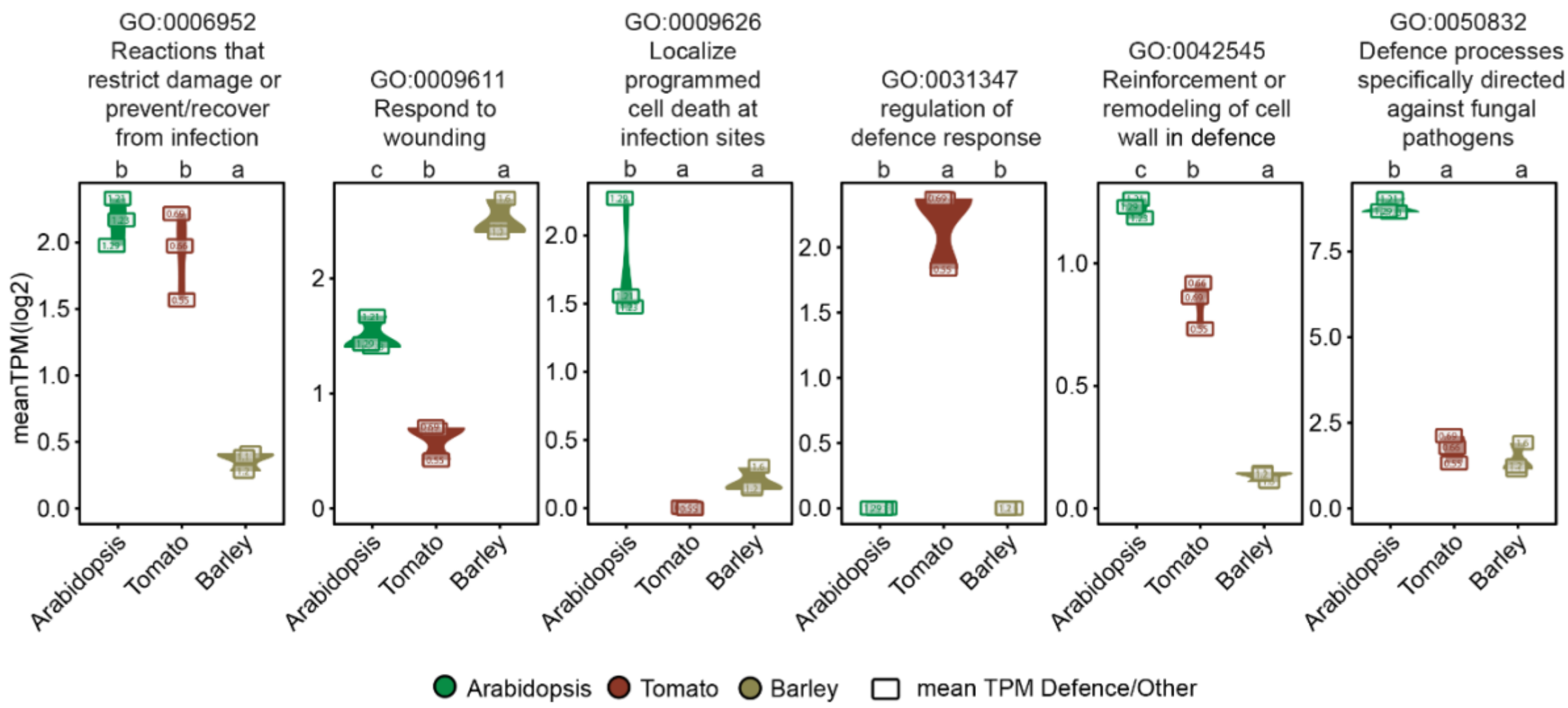

Supplementary Fig. 9. Host immune responses activated in roots in response to *P. cucumerina* 21 days post inoculation. The violin-plot indicates the distribution of mean TPM (log_2 scale, y-axis) from plant reads grouped for gene-ontology (GO) terms. The x-axes represent different plants (Arabidopsis, Tomato and Barley) indicated by colours. Each panel corresponds to GO terms that are associated with plant defence. The ratio of the mean TPM for plant defence to other GO-terms are represented in rectangles as each data points. The ratios of each datapoints are indicated within the rectangles. A value >1 indicates predominant expression of transcripts within the corresponding GO-term.

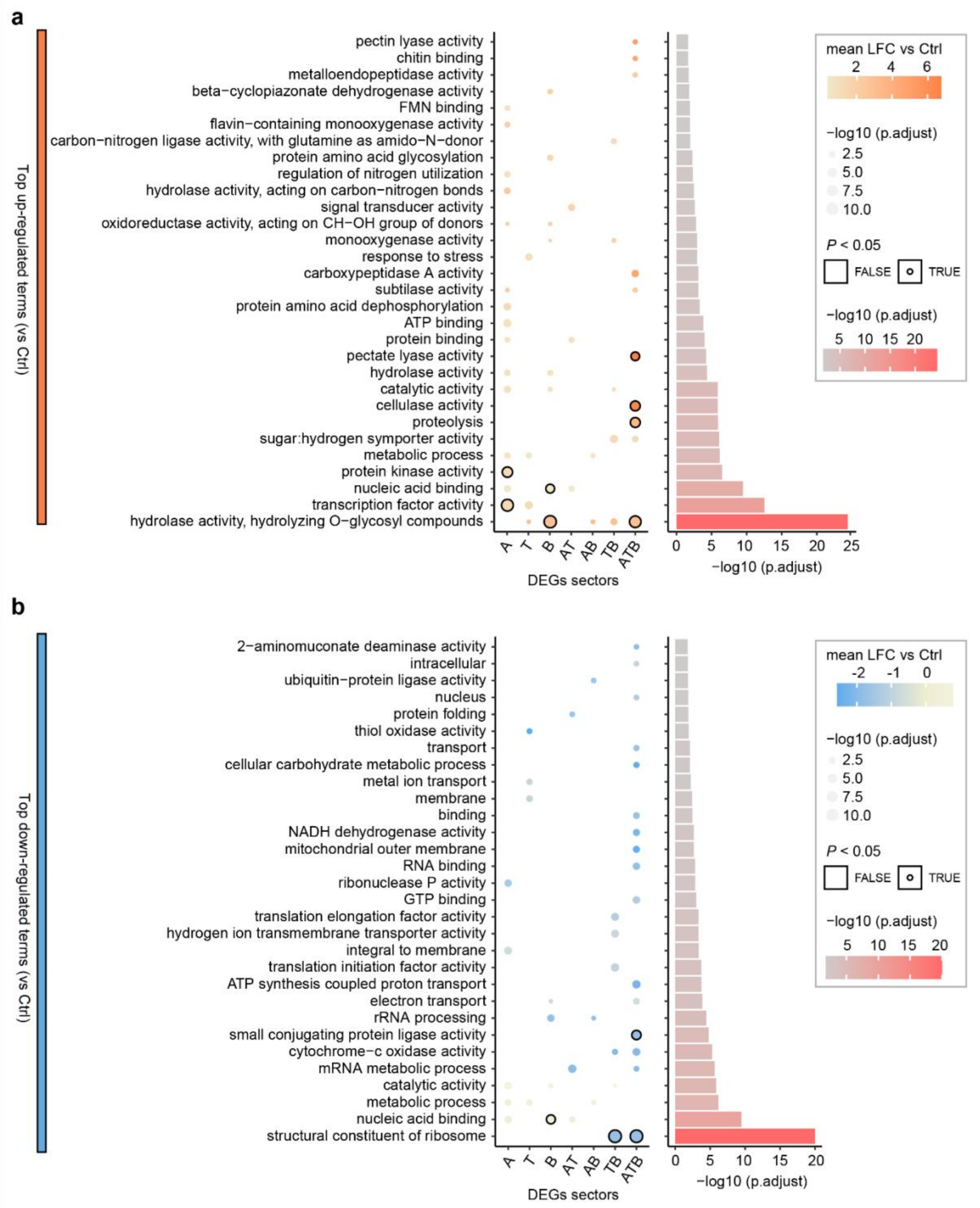

Supplementary Fig. 10: Fungal GO terms significantly regulated during root colonization. a,b, The heatmap illustrates the average $\log_2$ fold-change values for the categories (x-axis), marked circles signify $P \leq 0.05$ and the size of the circle indicates $-\log_{10}$ *P*-adjusted values (FDR, Fisher's Exact Test). The corresponding barplot shows the cumulative $-\log_{10}$ *adj. P* values (x-axis and colours grey-red) for the 30 most common GO-terms in y-axes. GO terms that are up-regulated are represented in orange (**a**) and those that are down-regulated are represented in blue (**b**).

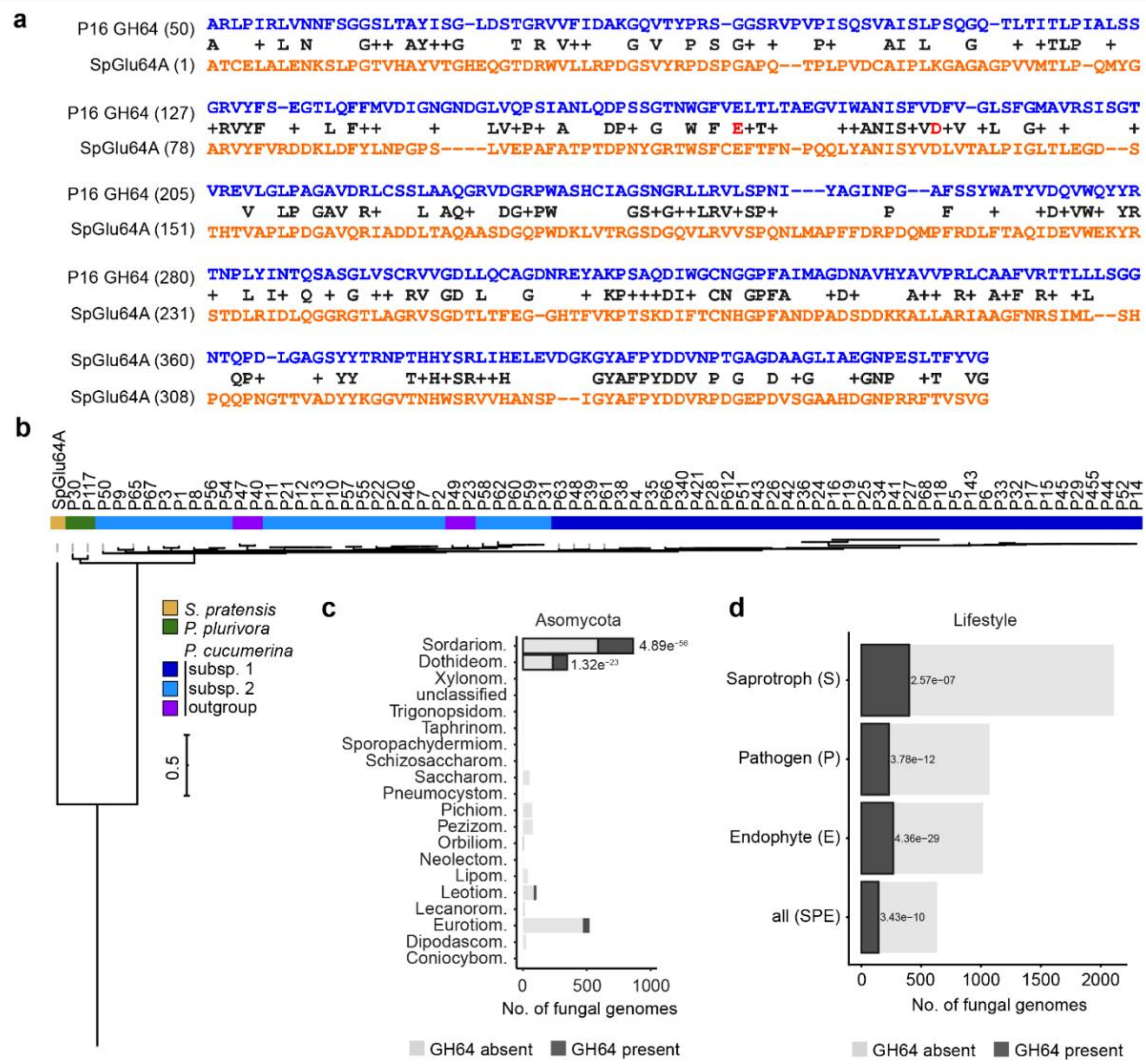

Supplementary Fig. 11: **Function and evolutionary conservation of GH64 in the fungal kingdom**. **a,** Structure-aware sequence alignment of *P. cucumerina* 0016 GH64 (in blue) with SpGlu64A (in orange) shows conservation of catalytic residues (in red), despite overall low sequence similarity (sequence identity=34.3%). **b,** Maximum-likelihood phylogenetic tree (model JTT+R2 of iqtree[3]) of the orthologs of *P. cucumerina* 0016 GH64 in the *Plectosphaerella* genome collection, together with SpGlu64A. Labels refer to the strain encoding the protein, while colour strips highlight the strain phylogenetic group. Note: except for *P. delsorboi* which does not encode any GH64 enzyme, a single GH64-encoding gene was annotated in every *Plectosphaerella* genome. **c,d,** Stacked bar plot depicting the presence/absence of GH64-encoding genes in 2,534 fungal genomes (x-axes) across distinct classes of Ascomycota members (**c**) and fungal lifestyles (**d**) (y-axes). Highlighted in dark grey are those where *GH64* family genes are encoded in the genome, and those with thick black borders are enriched within the group (Fisher's exact test followed by *P* value adjustment using Bonferroni correction).

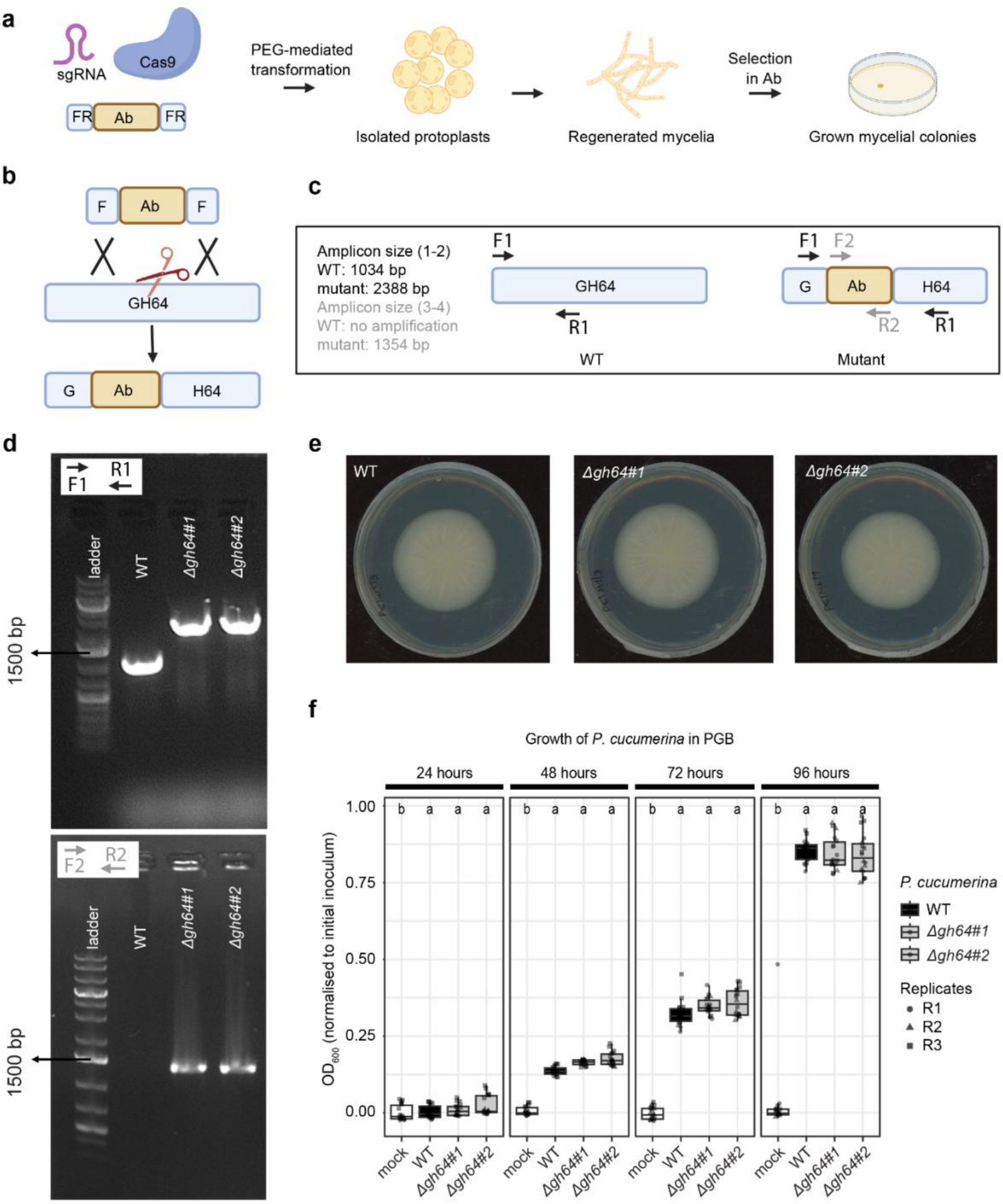

Supplementary Fig. 12: Protoplasts, regenerated Protoplasts and *P. cucumerina* 0016 mutants validation: a, Schematic diagram illustrating PEG-mediated transformation and CRISPR/Cas9- mediated knockout method. (PEG-mediated transformation method: 1. Isolation of protoplasts, 2. transformation of Donor DNA and Cas9-sgRNA complex into the protoplasts. 3. Regeneration of protoplasts. 4. Selection of potential colonies in media containing Phleomycin.) **b,** The CRISPR/Cas9 system enhances HR-mediated integration by introducing a double-stranded break in the target DNA, allowing for effective integration with short homology flanks of 60bp. **c**, Schematic representation of the *gh64* gene region in WT and in mutant fungi. Primer pairs used in panel d are depicted. **d**, Targeted disruption of the GH64 gene. Top: Agarose gel electrophoresis results using primer pair F1, R1. The wild type shows a band at 1034 bp, while mutants display a band shift to 2388 bp due to the insertion of the antibiotic resistance sequence. Lane 1: Ladder; Lane 2: Wild type (WT) control; Lane 3, 4:

Mutant 1, 2; Bottom: Amplification of the phleomycin antibiotic resistance cassette using primer pair F2, R2. The gel schema is identical to the top panel, confirming the presence of the phleomycin cassette in the mutant lines. Uncropped images are provided at the end of the document. **e,** *In vitro* growth of *P. cucumerina* 0016 WT *versus Δgh64* mutant strains in PGA plate. **f**, Dynamics of *in vitro* growth of *P. cucumerina* 0016 WT *versus Δgh64* mutant strains in PGB medium. Three independent biological replicates (n=18) are depicted (circles, triangles, squares). Multiple pairwise comparisons of plant performance between Mock, WT and mutants treated were performed using two-sided Kruskal-Wallis-Dunn Test, adj. with Bonferroni correction ($P< 0.05$). The exact *P* values are provided in Source Data file 1. Boxplots display the median (center line), lower and upper quartiles (box bounds), and whiskers extending to the most extreme data points within 1.5 × the IQR. Points beyond the whiskers are shown individually as outliers.

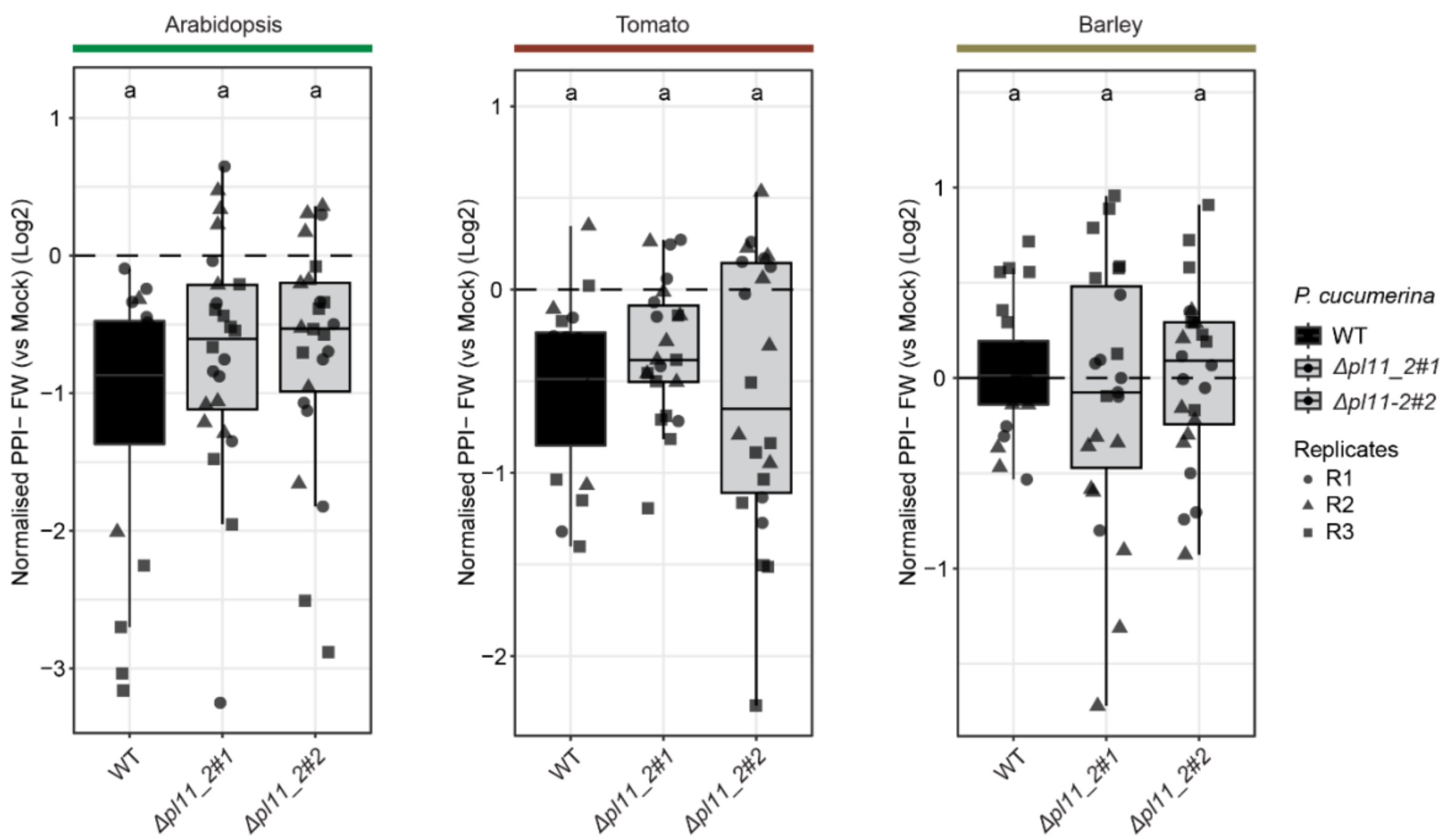

Supplementary Fig. 13: Phenotype assessment of *P. cucumerina 0016 Δpl11_2* mutant strains. Plant Performance Indices (PPI) of Arabidopsis, tomato, and barley colonized by *P. cucumerina* 0016 WT and mutant strains (*Δpl11_2#1*, *Δpl11_2#2*) were normalized to mock-treated plants and log2 transformed. PPI values were calculated by multiplying the germination rate by the shoot fresh weight of the plants. Pre-grown seedlings (2-week-old Arabidopsis and tomato and a week-old barley) were inoculated with *P. cucumerina* 0016 and harvested 21 days post fungal inoculation. Three independent biological replicates are depicted (circles, triangles, squares),and the values of n (WT/*Δpl11_2#1*/*Δpl11_2#2*) are Arabidopsis: 20/24/24, tomato: 20/22/22, and barley 24/24/24 respectively. Multiple pairwise comparisons of plant performance between mock-treated and *P. cucumerina* 0016 treated plants were performed using two-sided Kruskal-Wallis-Dunn Test, adj. with Bonferroni correction (*P*>0.05). Boxplots display the median (center line), lower and upper quartiles (box bounds), and whiskers extending to the most extreme data points within 1.5 × the IQR. Points beyond the whiskers are shown individually as outliers.

References

1. Giraldo A, Crous PW. Inside Plectosphaerellaceae. *Stud Mycol* **92**, 227-286 (2019).

2. Tedersoo L*, et al.* The Global Soil Mycobiome consortium dataset for boosting fungal diversity research. *Fungal Divers* **111**, 573-588 (2021).

3. Minh BQ, *et al.* IQ-TREE 2: New Models and Efficient Methods for Phylogenetic Inference in the Genomic Era. *Mol Biol Evol* **37**, 1530-1534 (2020).

4. Kalyaanamoorthy S, Minh BQ, Wong TKF, von Haeseler A, Jermiin LS. ModelFinder: fast model selection for accurate phylogenetic estimates. *Nat Methods* **14**, 587-589 (2017).

5. Emms DM, Kelly S. OrthoFinder: phylogenetic orthology inference for comparative genomics. *Genome Biol* **20**, 238 (2019).

6. Kozlov AM, Darriba D, Flouri T, Morel B, Stamatakis A. RAxML-NG: a fast, scalable and user-friendly tool for maximum likelihood phylogenetic inference. *Bioinformatics* **35**, 4453-4455 (2019).

7. Emms DM, & Kelly, S. . STAG: Species Tree Inference from All Genes. . *bioRxiv, doi: https://doiorg/101101/267914* (2018).

8. Zhang C, Sayyari E, Mirarab S. ASTRAL-III: Increased Scalability and Impacts of Contracting Low Support Branches. *Lect N Bioinformat* **10562**, 53-75 (2017).

9. Riehl K, Riccio C, Miska EA, Hemberg M. TransposonUltimate: software for transposon classification, annotation and detection. *Nucleic Acids Res*, (2022).

10. Zheng JF, Ge QW, Yan YC, Zhang XP, Huang L, Yin YB. dbCAN3: automated carbohydrate-active enzyme and substrate annotation. *Nucleic Acids Res* **51**, W115-W121 (2023).

11. Cantalapiedra CP, Hernández-Plaza A, Letunic I, Bork P, Huerta-Cepas J. eggNOG-mapper v2: Functional Annotation, Orthology Assignments, and Domain Prediction at the Metagenomic Scale. *Mol Biol Evol* **38**, 5825-5829 (2021).

12. Sperschneider J, Dodds PN. EffectorP 3.0: Prediction of Apoplastic and Cytoplasmic Effectors in Fungi and Oomycetes. *Mol Plant Microbe Interact* **35**, 146-156 (2022).

13. Cheng CY, Krishnakumar V, Chan AP, Thibaud-Nissen F, Schobel S, Town CD. Araport11: a complete reannotation of the Arabidopsis thaliana reference genome. *Plant J* **89**, 789-804 (2017).

14. Aoki K*, et al.* Large-scale analysis of full-length cDNAs from the tomato (Solanum lycopersicum) cultivar Micro-Tom, a reference system for the Solanaceae genomics. *Bmc Genomics* **11**, 210 (2010).

15. Beier S*, et al.* Construction of a map-based reference genome sequence for barley, Hordeum vulgare L. *Sci Data* **4**, 170044 (2017).

16. Patro R, Duggal G, Love MI, Irizarry RA, Kingsford C. Salmon provides fast and bias-aware quantification of transcript expression. *Nat Methods* **14**, 417-419 (2017).

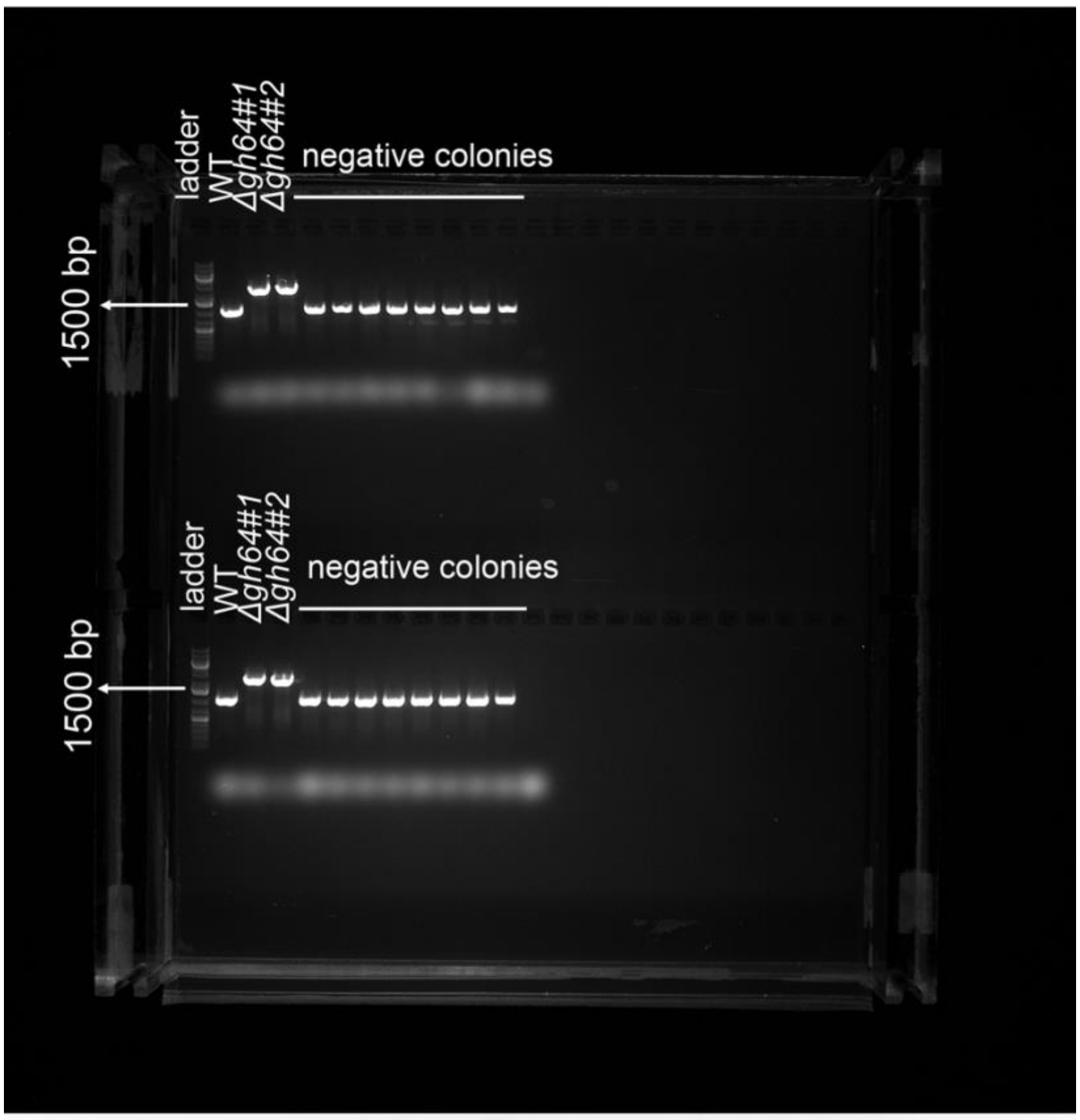

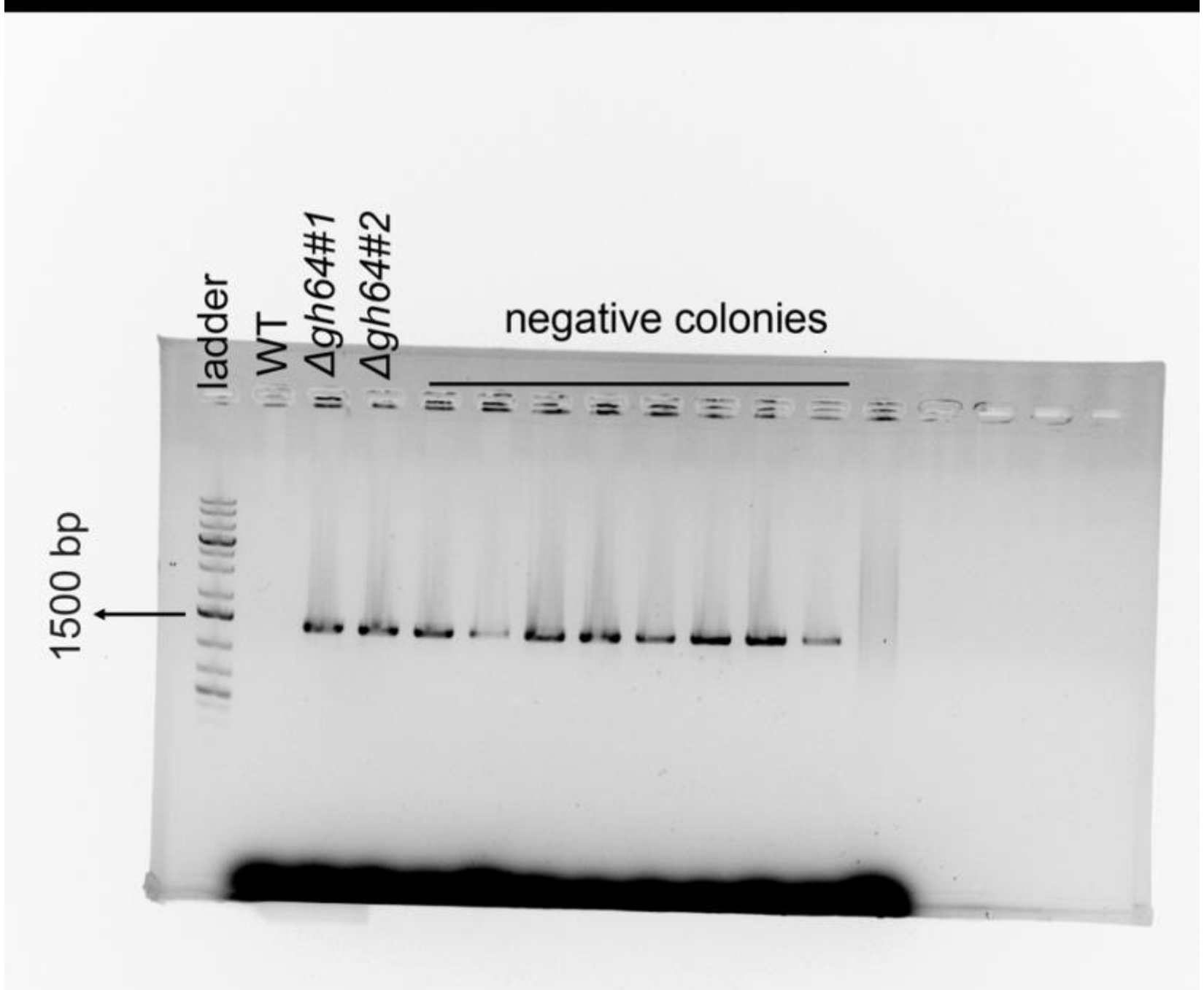

Uncropped images corresponding to Supplementary Figure 12d
