# Supplementary material for: Carbohydrate-active enzymes from a core root mycobiota member enable infection of multiple plant hosts
Source: Nat Microbiol. 2026 Oct 1;11(11):2975–93. doi: 10.1038/s41564-026-02492-3 (PMC13739743; doi:10.1038/s41564-026-02492-3)
Supplement: Supplementary file 2 [file 41564_2026_2492_MOESM2_ESM.pdf]

nature portfolio

Corresponding author(s): Stephane Hacquard

Last updated by author(s): Aug 5, 2026

Reporting Summary

Nature Portfolio wishes to improve the reproducibility of the work that we publish. This form provides structure for consistency and transparency in reporting. For further information on Nature Portfolio policies, see our Editorial Policies and the Editorial Policy Checklist.

Statistics

For all statistical analyses, confirm that the following items are present in the figure legend, table legend, main text, or Methods section.

n/a	Confirmed	
☐	☒	The exact sample size (*n*) for each experimental group/condition, given as a discrete number and unit of measurement
☐	☒	A statement on whether measurements were taken from distinct samples or whether the same sample was measured repeatedly
☐	☒	The statistical test(s) used AND whether they are one- or two-sided<br>*Only common tests should be described solely by name; describe more complex techniques in the Methods section.*
☒	☐	A description of all covariates tested
☐	☒	A description of any assumptions or corrections, such as tests of normality and adjustment for multiple comparisons
☐	☒	A full description of the statistical parameters including central tendency (e.g. means) or other basic estimates (e.g. regression coefficient) AND variation (e.g. standard deviation) or associated estimates of uncertainty (e.g. confidence intervals)
☐	☒	For null hypothesis testing, the test statistic (e.g. *F*, *t*, *r*) with confidence intervals, effect sizes, degrees of freedom and *P* value noted<br>*Give P values as exact values whenever suitable.*
☒	☐	For Bayesian analysis, information on the choice of priors and Markov chain Monte Carlo settings
☐	☒	For hierarchical and complex designs, identification of the appropriate level for tests and full reporting of outcomes
☐	☒	Estimates of effect sizes (e.g. Cohen's *d*, Pearson's *r*), indicating how they were calculated

Our web collection on statistics for biologists contains articles on many of the points above.

Software and code

Policy information about availability of computer code

Data collection	Softwares used: For collection of Shoot Fresh Weight- SPDC Data Collection V2.03, for qPCR- Bio-Rad CFX Manager 3.1, For gel electrophoresis documentation- BioVision Quantum CX5 Edge, For Biolog Plate Experiment- OmniLog® instrument (Biolog Inc.), For Confocal Microscopy- ZEN v2.3 SP1. For the Hydrolysis of Laminarihexaose by Plectosphaerella strains: Dionex ICS-6000 system operated with Chromeleon 7 software (version 7.3.1). qRT-PCR: CFX Opus 384 Real-Time PCR System (BIORAD). For OD 600 measurement: microplate reader (Tecan, Infinite M Nano). The structure of the GH64 protein from P. cucumerina 0016 was predicted with AlphaFold3, after signal peptide removal with SignalP v6.092. The best predicted model was then used as query for Foldseek. For the alignment of sequences, multialin and TM-align webtools were used.
Data analysis	For the graphs and statistical analysis, the R programming environment (R version 4.4.0) was used for data analysis and visualization (https://www.r-project.org/). The normality of the data was assessed with the Shapiro–Wilk test using stats package (V4.4.0) and homogeneity of variances with Levene's test using car package (V3.1-2). The two-sided T-tests and two-sided non-parametric Wilcox rank-sum test were performed using t.test and wilcox.test from the stats package (V4.4.0) in R. ANOVA-TukeyHSD tests were conducted using emmeans package V1.10.3). Non-parametric Kruskal-Wallis tests were carried out with stats package (V4.4.0), and Dunn's tests were performed using FSA (V0.9.5) and rcompanion packages (V2.4.36). Reported P values were Bonferroni adjusted using stats package (V4.4.0). Figures were assembled using Adobe illustrator. Confocal microscopy images were processed using Fiji software.<br>Scripts and raw data used to generate figures are available at https://github.com/fantin-mesny Scripts_comparison_of_Plectosphaerella_strains for WGS, https://github.com/arpankbasak/Plecuc1_Repo. For RNAseq, and https://github.com/RamSevakRK/Genetic-factors-driving-multi-host-infection-in-a-core-member-of-the-root-mycobiota for all the other experiments.

For manuscripts utilizing custom algorithms or software that are central to the research but not yet described in published literature, software must be made available to editors and reviewers. We strongly encourage code deposition in a community repository (e.g. GitHub). See the Nature Portfolio guidelines for submitting code & software for further information.

Data

Policy information about availability of data

All manuscripts must include a data availability statement. This statement should provide the following information, where applicable:

- Accession codes, unique identifiers, or web links for publicly available datasets
- A description of any restrictions on data availability
- For clinical datasets or third party data, please ensure that the statement adheres to our policy

RNASeq raw data are available at the European Nucleotide Archive (ENA) under the study ID PRJEB89666. The raw genomic data of the Plectosphaerella isolates have been deposited in the ENA database under the project IDs PRJEB61839 (P143), PRJEB50298 (P340, P421, P455) and PRJEB90544 (all other isolates). See Supplementary Table 3 for individual ENA sample IDs. Scripts and raw data used to generate figures are available at https://github.com/fantin-mesny/Scripts_comparison_of_Plectosphaerella_strains for WGS, https://github.com/arpankbasak/Plecuc1_Repo. For RNAseq, GH64 phylogeny, and recolonization experiment on Arabidopsis accessions, and https://github.com/RamSevakRK/Genetic-factors-driving-multi-host-infection-in-a-core-member-of-the-root-mycobiota for all the other experiments.

Research involving human participants, their data, or biological material

Policy information about studies with human participants or human data. See also policy information about sex, gender (identity/presentation), and sexual orientation and race, ethnicity and racism.

Reporting on sex and gender	NA
Reporting on race, ethnicity, or other socially relevant groupings	NA
Population characteristics	NA
Recruitment	NA
Ethics oversight	NA

Note that full information on the approval of the study protocol must also be provided in the manuscript.

Field-specific reporting

Please select the one below that is the best fit for your research. If you are not sure, read the appropriate sections before making your selection.

☒ Life sciences ☐ Behavioural & social sciences ☐ Ecological, evolutionary & environmental sciences

For a reference copy of the document with all sections, see nature.com/documents/nr-reporting-summary-flat.pdf

Life sciences study design

All studies must disclose on these points even when the disclosure is negative.

Sample size	No statistical methods were used to pre-determine sample sizes. Previous experiments using the similar gnotobiotic system were used as reference to determine the sample size (Mesny et al., 2021. Nature Communication). For each figure, the sample size is indicated as 'n' in the respective figure legend and the statistical tests were selected based on the sample size and experimental design.
Data exclusions	No data excluded
Replication	All the experiments were performed independently with atleast 3 biological replicates, to measure the robustness of the biological findings. All replications were successful.
Randomization	During the experiment, the position of the magenta boxes in the growth chamber were randomized every week. At sowing, seeds were allocated by plate to pre-defined groups (fungus-inoculated or mock) and plates were labeled accordingly. Plate positions in the growth chamber were re-randomized twice per week during the 1–2 week pre-treatment period to minimize positional effects. After ~2 weeks, seedlings were inoculated according to the pre-assigned label, with identical handling and timing for both groups. Following inoculation, pots/boxes were re-randomized within the chamber twice per week for the remainder of the experiment.
Blinding	The experiments were performed, harvested and processed by multiple researchers. Each condition was harvested by every researcher. The samples from all the conditions were analyzed by single researcher.

Reporting for specific materials, systems and methods

We require information from authors about some types of materials, experimental systems and methods used in many studies. Here, indicate whether each material, system or method listed is relevant to your study. If you are not sure if a list item applies to your research, read the appropriate section before selecting a response.

Materials & experimental systems

n/a	Involved in the study
☒	☐ Antibodies
☒	☐ Eukaryotic cell lines
☒	☐ Palaeontology and archaeology
☒	☐ Animals and other organisms
☒	☐ Clinical data
☒	☐ Dual use research of concern
☐	☒ Plants

Methods

n/a	Involved in the study
☒	☐ ChIP-seq
☒	☐ Flow cytometry
☒	☐ MRI-based neuroimaging

Plants

Seed stocks	Arabidopsis thaliana (accessions: Van-0, Se-0, Bd-0, Col-0, Ct-1, Mh-0, Ws-2, Ull2-3, Kin-0, and RRS-7), Tomato (Solanum lycopersicum cv. Micro-Tom), Barley (Hordeum vulgare)
Novel plant genotypes	We used Arabidopsis Col-0 (Arabidopsis thaliana Columbia-0), Tomato (Solanum lycopersicum cv. Micro-Tom), Barley (Hordeum vulgare) as the model plants in our study. All are them are wild types. We have not generated any novel plant genotypes in this study.
Authentication	No additional genotypic authentication were performed.
