# Supplementary material for: Carbohydrate-active enzymes from a core root mycobiota member enable infection of multiple plant hosts
Source: Nat Microbiol. 2026 Oct 1;11(11):2975–93. doi: 10.1038/s41564-026-02492-3 (PMC13739743; doi:10.1038/s41564-026-02492-3)
Supplement: Supplementary file 3 [file 41564_2026_2492_MOESM3_ESM.pdf]

nature portfolio

Peer Review File

Carbohydrate-active enzymes from a core root mycobiota member enable infection of multiple plant hosts

Corresponding Author: Dr Stéphane Hacquard

Version 0:

Decision Letter:

12th February 2026

*Please ensure you delete the link to your author homepage in this e-mail if you wish to forward it to your co-authors.

Dear Dr Hacquard,

Thank you for your patience while your manuscript "Genetic factors driving multi-host infection in a core member of the root mycobiota" was under peer-review at Nature Microbiology. It has now been seen by 3 referees, whose expertise and comments you will find at the end of this email. Although they find your work of some potential interest, they have raised a number of concerns that will need to be addressed before we can consider publication of the work in Nature Microbiology.

In particular, Reviewers #2 and #3 have recommended additional experimental validation work, which we believe would further strengthen the findings presented here, in addition to this there are several areas which require further analyses, clarification or discussion, particularly we are keen to understand the ecological relevance and impact of this work addressed as suggested by Reviewer #3. Should you be able to conduct further experimental work to address these criticisms, we would be happy to look at a revised manuscript.

We are committed to providing a fair and constructive peer-review process. Please do not hesitate to contact us if there are specific requests from the reviewers that you believe are technically impossible or unlikely to yield a meaningful outcome.

We strongly support public availability of data. Please place the data used in your paper into a public data repository, if one exists, or alternatively, present the data as Source Data or Supplementary Information. If data can only be shared on request, please explain why in your Data Availability Statement, and also in the correspondence with your editor. For some data types, deposition in a public repository is mandatory - more information on our data deposition policies and available repositories can be found at https://www.nature.com/nature-research/editorial-policies/reporting-standards#availability-of-data.

Please include a data availability statement as a separate section after Methods but before references, under the heading "Data Availability". This section should inform readers about the availability of the data used to support the conclusions of your study. This information includes accession codes to public repositories (data banks for protein, DNA or RNA sequences, microarray, proteomics data etc…), references to source data published alongside the paper, unique identifiers such as URLs to data repository entries, or data set DOIs, and any other statement about data availability. At a minimum, you should include the following statement: "The data that support the findings of this study are available from the corresponding author upon request", mentioning any restrictions on availability. If DOIs are provided, we also strongly encourage including these in the Reference list (authors, title, publisher (repository name), identifier, year). For more guidance on how to write this section please see: http://www.nature.com/authors/policies/data/data-availability-statements-data-citations.pdf

If revising your manuscript:

* Include a "Response to referees" document detailing, point-by-point, how you addressed each referee comment. If no action was taken to address a point, you must provide a compelling argument. This response will be sent back to the referees along with the revised manuscript.

* If you have not done so already we suggest that you begin to revise your manuscript so that it conforms to our Article format instructions at http://www.nature.com/nmicrobiol/info/final-submission. Refer also to any guidelines provided in this letter.

* Include a revised version of any required reporting checklist. It will be available to referees (and, potentially, statisticians) to aid in their evaluation if the manuscript goes back for peer review. A revised checklist is essential for re-review of the paper.

When submitting the revised version of your manuscript, please pay close attention to our href="https://www.nature.com/nature-portfolio/editorial-policies/image-integrity">Digital Image Integrity Guidelines. and to the following points below:

-- that unprocessed scans are clearly labelled and match the gels and western blots presented in figures.
-- that control panels for gels and western blots are appropriately described as loading on sample processing controls
-- all images in the paper are checked for duplication of panels and for splicing of gel lanes.

EXTENDED DATA FIGURES

When re-submitting your manuscript, please ensure that any supplementary figures and tables that are crucial to the manuscript's conclusions are converted into Extended Data figures and tables to increase visibility of these data. Extended Data figures and tables are online-only (present in the online PDF and full-text HTML versions of the paper), peer-reviewed display items that provide essential background to the article but are not included in the main article due to space constraints. A maximum of ten Extended Data display items (figures and tables) is permitted.

Finally, please ensure that you retain unprocessed data and metadata files after publication, ideally archiving data in perpetuity, as these may be requested during the peer review and production process or after publication if any issues arise.

Please use the link below to submit a revised paper:

Link Redacted

Note: This url links to your confidential homepage and associated information about manuscripts you may have submitted or be reviewing for us. If you wish to forward this e-mail to co-authors, please delete this link to your homepage first.

Nature Microbiology is committed to improving transparency in authorship. As part of our efforts in this direction, we are now requesting that all authors identified as 'corresponding author' on published papers create and link their Open Researcher and Contributor Identifier (ORCID) with their account on the Manuscript Tracking System (MTS), prior to acceptance. This applies to primary research papers only. ORCID helps the scientific community achieve unambiguous attribution of all scholarly contributions. You can create and link your ORCID from the home page of the MTS by clicking on 'Modify my Springer Nature account'. For more information please visit please visit www.springernature.com/orcid.

If you wish to submit a suitably revised manuscript we would hope to receive it within 6 months. If you cannot send it within this time, please let us know. We will be happy to consider your revision, even if a similar study has been accepted for publication at Nature Microbiology or published elsewhere (up to a maximum of 6 months).

In the meantime we hope that you find our referees' comments helpful.

Yours sincerely,

Neem J. Patel, PhD
Associate Editor
Nature Microbiology

****
Reviewer Expertise:

Referee #1: plant-fungal interactions
Referee #2: plant-fungal interactions, genomics
Referee #3: plant root - microbiome interactions and communication

Reviewer Comments:

Reviewer #1 (Remarks to the Author):

The authors aimed to identify the genetic factors that determine how a core fungal member of Arabidiopsis' mycobiota colonizes and interacts with multiple plant hosts (i.e., Arabidopsis, tomato, and barley). A combination of whole genome sequencing, transcriptomics, inoculation-based assays, functional genetics, and microscopy were used to identify a candidate glucanase-encoding gene (GH64) as a driver of fungal root colonization and multi-host plant disease. The authors further demonstrate that this gene diverged 110 mya, illustrating that GH64 functions as a root colonizing determinant for P. cucumerina in their two tested dicot host plants (not barley) but enables pathogenic functions for the root pathogen C. incanum on Arabidopsis but not tobacco.

Overall, the authors present a compelling case for the genetic basis of GH64 across several plant and fungal species. The use of multiple hosts coupled with the initial pass of showing that P. cucumerina is a core root mycobiota member and then expanding these co-occurrences to other plant hosts is particularly informative, compelling, and appreciated. (Though I'll add that the authors could strengthen this global assessment by connecting the environmental and/or edaphic factors that may foster P. cucumerina competitive ability and pathogenic potential – under the assumption that these data are available. This I think would

help gain insights into how P. cucumerina would potentially impact host plants in different contexts such as anticipated climatic scenarios. I however do not think this is a necessary addition to the manuscript, only a point for future consideration.) Nevertheless, I find this study to be interesting and likely to be of broad interest to readers across fields. Below I have highlighted specific comments to improve the manuscript's clarity and scope.

Line 169: List F statistic, DF, etc. when reporting PERMANOVA results.

Line 173: I think the message here should be a bit more conservative. Assessing phenotypic differentiation of P. cucumerina strains in this fashion may not be as indicative of their lifestyles as expressed by the authors. 1) The gnotobiotic conditions largely ignore multipartite interactions, and 2) Biolog plates ignore the problem of 'what happens when a given strain is provided with multiple resources at once and at differing concentrations?' I generally agree with the author's findings here, but some care should be taken to acknowledge the artificial nature of these assays. Statements like those mentioned in Line 333 are good examples of giving the text balance and careful consideration.

Line 316: The logic of why C. incanum was used is not readily apparent to me. To enhance the readability of this section transition, I'd suggest spelling this out for the reader a bit more clearly.

Line 324: Overall, I think that the discussion section would benefit from some acknowledgements to the limitations of the study. Microbial complexity, diversity, etc. can alter interactions. How for example would the author's observations change in ecologically meaningful ways if both field derived soils w/ live and sterile conditions were implemented? I nevertheless acknowledge the immense amount of work that the authors conducted, but I do think that some attempts to better balance ecological realism and mechanistic insights would provide some helpful context and ways forward for readers.

Line 355: PCWDEs or lack thereof also play a role in mycorrhizal symbioses. Though Arabidopsis is notably non-mycorrhizal, some natural extensions or acknowledgement here could deepen the eco-evolutionary messages the authors present.

Line 368: Some pause should be given to the fact this only applies to two dicots.

Line 376: Why dispensable for tobacco root infection?

Line 603: Was the vermiculite sterilized? If not, what microbes might be there?

Line 684: The FungusOnMembrane control sounds like it would be even more artificial than the experimental group? Clarity and some additional rationale would be helpful here. How can you be sure that the signal between in planta and ex planta is truly indicative of host plant identity and not some unintended experimental bias?

Line 1424: Why fresh weight and not dry weight? And is this PPI standard practice? I do not think that fresh weight x survivability alone captures host plant performance. How were shoot height, root length, R:S ratio, silique number, seed quantity + quality impacted? Biological systems are essentially a long list of tradeoffs. Careful consideration should be taken when estimating plant performance.

Reviewer #2 (Remarks to the Author):

This manuscript reanalyzed two major metagenomics datasets published in 2018 (Cell) and 2020 (Nat. Ecol Evol). The authors very nicely characterize the structure and content of fungal taxa detected in diverse soil and root samples. I expect that the manuscript will add value to the plant-microbe interaction field. However, several major flaws dimmed the enthusiasm for my recommendation.

First: the authors identified 5 Zotus that are plotted according to their prevalence and mean log-transformed relative abundance (RA) in roots across all samples (line 126-128 and Fig 1a): Zotu2 (Fusarium sp.), Zotu9 (Plectosphaerella sp.), Zotu1 (Dactylonectria sp.), Zotu6 (Fusarium sp.) and Zotu3 (Fusarium sp.) that have occurrence values of 1, 0.98, 0.92, 0.87 and 0.87, respectively. However, the authors dived into Zotu9 without paying attention to other 4 Zotus.

Suggestion 1: at least perform a similar analysis for Zotu2 with the occurrence value of 1.
Suggestion 2: make it very clear in the title, abstract and the text that this study focuses on "one of the core members"

Second: using the P. cucumerina 0016 for the rest of the paper is biased. The strain 0016 was isolated from Arabidopsis, the results presented here show the host adaptation as illustrated in Fig. 3.

Suggestion 1: It will be important to conduct in planta experiments using strains isolated from the same host. You can not expect to see true interactions using strains isolated from dicots to a monocot host. We know that host-specific adaptation is real! Since this study has generated genome assemblies for 72 isolates, why not identify a strain isolated from a monocot host.
Suggestion 2: RNAseq of the corresponding interactions will be more revealing.

One minor suggestion:
Fig 3 images are hard to tell which cell layers the fungal hyphae have reached. Maybe a cross section can be more revealing

Reviewer #3 (Remarks to the Author):

This review relates to the manuscript entitled "Genetic factors driving multi-host infection in a core member of the root mycobiota" by Raja-Kumar et al.

The manuscript describes the identification of Plectosphaerella cucumerina as a core member of the Arabidopsis root microbiome. It is first observed in a set of samples from Europe that were part of previous studies and through analysis of global data identified in diverse plant-associated samples worldwide. Next, high-quality genome sequences for a set of purified cultures from this species were obtained and used to study host preference and Arabidopsis pathogenicity. No specific correlations between genetic factors and host preference, pathogenicity or carbon utilization profiles were identified. Most isolates were pathogenic on Arabidopsis, and for two strains specifically it was shown that pathogenicity is particularly prevalent on dicot hosts although the species is observed equally prevalent on monocot. This was further corroborated by effective fungal colonization on a monocot host, but in the absence of symptomology. Transcriptome profiling indicates diverse activation of cell-wall degrading enzymes and some commonalities during colonization of similar hosts. Specifically, a GH64-encoding gene was activated on all three hosts, and targeted mutagenesis shows that this gene is required for full pathogenicity on dicot hosts. Colonization levels were dramatically reduces on dicot hosts suggesting it is required for pathogen proliferation. On monocot hosts, contrastingly, such decrease in fungal proliferation was not observed.

Overall, it was a joy to read this manuscript. It was easy to read and easy to follow the data presentation that is clean and clear. I have only a few comments with regards to the data analysis and the manuscript.

- The authors focused on zero-radius OTUs and refer to the methods for explanation. I didn't find this explanation.
- It is not sufficiently clear why the authors focus on Plectosphaerella except for the fact that they have perhaps the largest number of representative isolates in the collection. The diverse Fusaria zOTUs seem equally interesting from the current text.
- In general I am missing context on the focal species. What do we know about Plectosphaerella pathogenicity, host preference.
- I am missing discussion of the ecological benefit of endophytic colonization in the context of fungal load on dicots and monocots. It seems that although there is limited/no endophytic colonization on the monocot hosts, fungal proliferation is equally "good" if not better. In the presence of a non-diseased plant this seems rather beneficial. How do the authors look at this?
- For visualization purpose, perhaps the authors could generate PCA (not PCoA, I presume the actual genes/CAZYs are used, so not PCoA as the legend suggest?) plots like the ones in figure 2 in the absence of some of the outlier species (e.g., P. delsorboi). It would allow to see maybe a bit more subspecies separation/structure in cucumerina.
- Plant gene expression is explicitly ignored in this study (allowing for focus), but it prevents deeper interpretation of the monocot versus dicot colonization which is a key component of this study.

****

Version 1:

Decision Letter:

Our ref: NMICROBIOL-25114187A

10th July 2026

Dear Dr. Hacquard,

Thank you for submitting your revised manuscript "Genetic factors driving multi-host infection in a core member of the root mycobiota" (NMICROBIOL-25114187A). It has now been seen by the original referees and their comments are below. The reviewers find that the paper has improved in revision, and therefore we'll be happy in principle to publish it in Nature Microbiology, pending minor revisions to satisfy the referees' final requests and to comply with our editorial and formatting guidelines.

If the current version of your manuscript is in a PDF format, please email us a copy of the file in an editable format (Microsoft Word or LaTex)-- we can not proceed with PDFs at this stage.

We are now performing detailed checks on your paper and will send you a checklist detailing our editorial and formatting requirements in a couple of weeks. Please do not upload the final materials and make any revisions until you receive this additional information from us.

Thank you again for your interest in Nature Microbiology Please do not hesitate to contact me if you have any questions.

Sincerely,

Neem J. Patel, PhD

Associate Editor
Nature Microbiology

Reviewer #2 (Remarks to the Author):

Thank you for the careful revision. I appreciate your thoroughly revised manuscript.

Reviewer #3 (Remarks to the Author):

The authors have addressed my feedback appropriately. I have no critical remaining comments, except to say that I agree with the authors that the P. cucumerina-specific PCoA plots can stay in supplementary (fig S6). Small comment there, the panel legends (a,b) write Ortogroup rather than Orthogroup. I couldn't find this back, but do the authors have an hypothesis about the "outlier" P. cucumerina strain from subsp1 group that in the case of effectors groups with the outgroup, and for cazymes and proteases sets itself apart?

INTERNAL USE ONLY AIP modules required: ["Life Science"]

Version 2:

Decision Letter:

18th August 2026

Dear Dr Hacquard,

I am pleased to accept your Article "Carbohydrate-active enzymes from a core root mycobiota member enable infection of multiple plant hosts" for publication in Nature Microbiology. Thank you for having chosen to submit your work to us and many congratulations.

Over the next few weeks, your paper will be copyedited to ensure that it conforms to Nature Microbiology style. We look particularly carefully at the titles of all papers to ensure that they are relatively brief and understandable.

Once your paper is typeset, you will receive an email with a link to choose the appropriate publishing options for your paper and our Author Services team will be in touch regarding any additional information that may be required. Once your paper has been scheduled for online publication, the Nature press office will be in touch to confirm the details.

You may wish to make your media relations office aware of your accepted publication, in case they consider it appropriate to organize some internal or external publicity. Once your paper has been scheduled you will receive an email confirming the publication details. This is normally 3-4 working days in advance of publication. If you need additional notice of the date and time of publication, please let the production team know when you receive the proof of your article to ensure there is sufficient time to coordinate. Further information on our embargo policies can be found here:
https://www.nature.com/authors/policies/embargo.html

After the grant of rights is completed, you will receive a link to your electronic proof via email with a request to make any corrections within 48 hours. If, when you receive your proof, you cannot meet this deadline, please inform us at rjsproduction@springernature.com immediately. You will not receive your proofs until the publishing agreement has been received through our system

Due to the importance of these deadlines, we ask you please us know now whether you will be difficult to contact over the next month. If this is the case, we ask you provide us with the contact information (email, phone and fax) of someone who will be able to check the proofs on your behalf, and who will be available to address any last-minute problems.

Acceptance of your manuscript is conditional on all authors' agreement with our publication policies (see https://www.nature.com/nmicrobiol/editorial-policies). In particular your manuscript must not be published elsewhere.

Authors may need to take specific actions to achieve compliance with funder and institutional open access mandates. If your research is supported by a funder that requires immediate open access (e.g. according to Plan S principles or the NIH public access policy) then you should select the gold OA route, and we will direct you to the compliant route where possible. Because authors warrant under our subscription licensing terms that they haven’t committed to licensing any version of their article under a licence inconsistent with the terms of our agreement – including the applicable embargo period – publication under the subscription model isn’t suitable for authors whose funders require no embargo.

If you have any questions about our publishing options, costs, Open Access requirements, or our legal forms, please contact ASJournals@springernature.com

An online order form for reprints of your paper is available at https://www.nature.com/reprints/author-reprints.html. All co-authors, authors' institutions and authors' funding agencies can order reprints using the form appropriate to their geographical region.

We welcome the submission of potential cover material (including a short caption of around 40 words) related to your manuscript; suggestions should be sent to Nature Microbiology as electronic files (the image should be 300 dpi at 210 x 297 mm in either TIFF or JPEG format). Please note that such pictures should be selected more for their aesthetic appeal than for their scientific content, and that colour images work better than black and white or grayscale images. Please do not try to design a cover with the Nature Microbiology logo etc., and please do not submit composites of images related to your work. I am sure you will understand that we cannot make any promise as to whether any of your suggestions might be selected for the cover of the journal.

You can now use a single sign-on for all your accounts, view the status of all your manuscript submissions and reviews, access usage statistics for your published articles and download a record of your refereeing activity for the Nature journals.

To assist our authors in disseminating their research to the broader community, our SharedIt initiative provides you with a unique shareable link that will allow anyone (with or without a subscription) to read the published article. Recipients of the link with a subscription will also be able to download and print the PDF.

As soon as your article is published, you will receive an automated email with your shareable link.

With kind regards,

Neem J. Patel, PhD
Associate Editor
Nature Microbiology

P.S. Click on the following link if you would like to recommend Nature Microbiology to your librarian http://www.nature.com/subscriptions/recommend.html#forms

** Visit the Springer Nature Editorial and Publishing website at www.springernature.com/editorial-and-publishing-jobs for more information about our career opportunities. If you have any questions please click here.**

Open Access This Peer Review File is licensed under a Creative Commons Attribution 4.0 International License, which permits use, sharing, adaptation, distribution and reproduction in any medium or format, as long as you give appropriate credit to the

original author(s) and the source, provide a link to the Creative Commons license, and indicate if changes were made.
In cases where reviewers are anonymous, credit should be given to 'Anonymous Referee' and the source.
The images or other third party material in this Peer Review File are included in the article's Creative Commons license, unless indicated otherwise in a credit line to the material. If material is not included in the article's Creative Commons license and your intended use is not permitted by statutory regulation or exceeds the permitted use, you will need to obtain permission directly from the copyright holder.
To view a copy of this license, visit https://creativecommons.org/licenses/by/4.0/
